# Preparation of a Series of Supported Nonsymmetrical PNP‐Pincer Ligands and the Application in Ester Hydrogenation

**DOI:** 10.1002/chem.201903379

**Published:** 2019-11-04

**Authors:** Robert Konrath, Anke Spannenberg, Paul C. J. Kamer

**Affiliations:** ^1^ School of Chemistry University of St Andrews North Haugh St Andrews Fife KY16 9ST UK; ^2^ Leibniz-Institut für Katalyse e. V. (LIKAT) an der Universität Rostock Albert-Einstein Strasse 29a 18059 Rostock Germany

**Keywords:** catalyst immobilization, heterogeneous catalysis, hydrogenation, pincer ligands, solid-phase synthesis

## Abstract

In contrast to their symmetrical analogues, nonsymmetrical PNP‐type ligand motifs have been less investigated despite the modular pincer structure. However, the introduction of mixed phosphorus donor moieties provides access to a larger variety of PNP ligands. Herein, a facile solid‐phase synthesis approach towards a diverse PNP‐pincer ligand library of 14 members is reported. Contrary to often challenging workup procedures in solution‐phase, only simple workup steps are required. The corresponding supported ruthenium‐PNP catalysts are screened in ester hydrogenation. Usually, industrially applied heterogeneous catalysts require harsh conditions in this reaction (250–350 °C at 100–200 bar) often leading to reduced selectivities. Heterogenized reusable Ru‐PNP catalysts are capable of reducing esters and lactones selectively under mild conditions.

## Graphical abstract

**Tune the pincer**: A solid‐phase synthesis approach is described to prepare a diverse library of nonsymmetrical PNP pincer ligands. The heterogenized library is applied in ruthenium‐catalyzed hydrogenation of esters and lactones under mild conditions. Catalyst recovery and recyclability are facilitated by covalent attachment to solid supports.

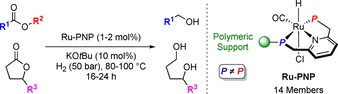

## Introduction

**Figure 1:**
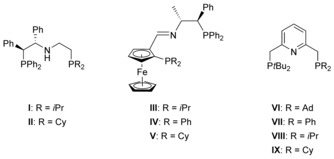
Representative examples of nonsymmetrical PNP pincer ligands.

Terdentate pincer‐type ligands have attracted tremendous attention for applications in a broad range of catalytic reactions since the pioneering work of Shaw and van Koten in the 1970s.^1^ Given that the modular nature of pincer ligands allows for efficient fine‐tuning of the electronic and steric properties,^2^ symmetrical PNP pincer ligands, which feature a central N‐donor and two identical phosphorus moieties, have been studied extensively in the last two decades. Although nonsymmetrical PNP ligands give access to a significantly increased number of potential ligand structures with unique stereo‐electronic properties, reports remain fairly limited.^3^ This can be attributed to the often more challenging synthesis and troublesome purification procedures required for nonsymmetrical pincers opposed to simplified twofold‐substitution protocols for ligands with *C*_2*v*_ symmetry. In case of representative chiral PNP pincers **I**–**V**, ligand desymmetrization was achieved through additional substituents in the aliphatic backbone as well as through mixed phosphorus‐donor moieties (Figure 1).^4^ To the best of our knowledge, ligands **VI**–**IX** reported by Kinoshita et al. remain the sole examples of nonsymmetrical pyridine‐based PNP ligands which differ in the nature of the phosphines.^5^ Structures **VI**–**IX**, composed of a P(*t*Bu)_2_ group and a second P‐donor bearing alkyl and aryl substituents, were prepared by successive deprotonation and mono‐substitution of 2,6‐lutidine using various chlorophosphines.

Regardless of the advances in rational design of high‐performance ligands, synthetic approaches through trial‐and‐error remain the most common methodologies for catalyst optimization. There is, however, still a lack of efficient combinatorial methods enabling the synthesis and screening of large ligand libraries, especially for phosphorus‐based multidentate ligands.^6^

Although modular approaches towards symmetrical pyridine‐based PNP pincer ligands have been explored by Kirchner and co‐workers,^7^ facile synthetic protocols towards large combinatorial ligand libraries of nonsymmetrical PNP‐type ligands remain elusive.

Solid‐phase synthesis (SPS), originating from well‐established polypeptide synthesis, offers an attractive alternative tool towards ligand libraries.^8^ The main advantage of SPS over traditional solution‐phase approaches is the ease of purification, often requiring only a simple filtration step and allowing for the use of a large excess of reactants.^9^ Systematic variation of substituents bound to the phosphine moieties enables the preparation of a large combinatorial PNP ligand library through SPS. This facilitates the finetuning of ligand properties for catalyst optimization.

Moreover, catalyst immobilization on insoluble supports combines the advantages of both worlds, that is, high activity, selectivity and tunability of homogeneous catalysts and the recoverability and recyclability of heterogeneous catalysts.^10^ In particular, the recycling of these expensive and often toxic transition metals and ligands can be truly simplified.

Notwithstanding the wide applicability of PNP pincer‐based catalysts, approaches towards immobilization strategies remain fairly limited. Goni et al. reported on a Ru‐PONOP‐type catalyst supported on a silica poly(allylamine) composite through a two‐step Mannich reaction yielding two regioisomers covalently bound to the solid in both *ortho*‐ and *meta*‐position of the central pyridine ring.^11^ Similarly, a phosphine oxide PNP ligand was anchored onto mesoporous silica through a Cu‐catalyzed click reaction by Lo et al.^12^ Upon reduction to the free supported phosphine, the corresponding Ir‐PNP catalyst was applied in CO_2_ hydrogenation. Wang et al. employed a “knitting” strategy by anchoring a solution‐phase Ru‐PNP catalyst covalently to the structure of a porous organic polymer for application in dehydrogenation of formic acid.^13^ A supported ionic‐liquid phase (SILP) strategy was chosen by the group of Kirchner for the immobilization of a Fe‐PNP catalyst in ionic liquids deposited on both silica^14^ and polymer‐based spherical activated carbon.^15^ However, in all cases a single premade PNP ligand or complex is immobilized missing the opportunity for efficient ligand modification. This calls for a more versatile and combinatorial methodology that allows for the facile synthesis of a diverse PNP‐ligand library.

**Figure 2:**
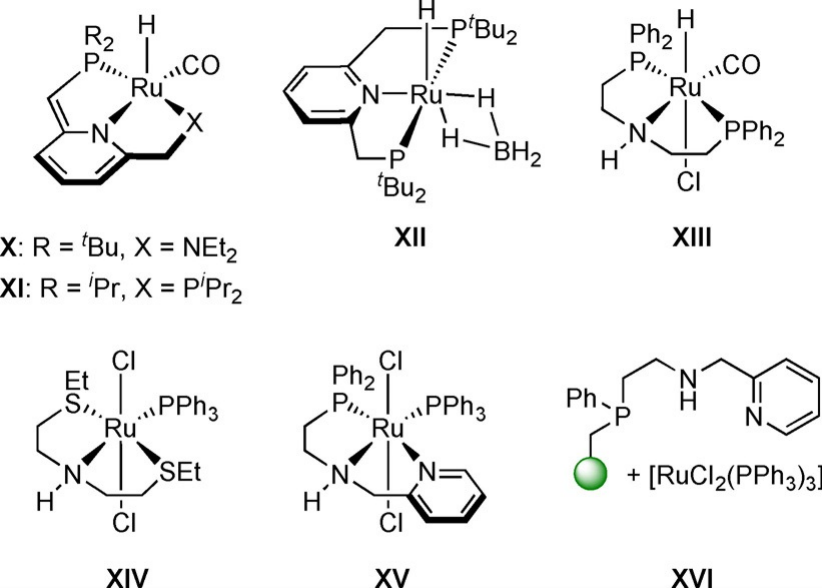
Representative examples of pincer‐based ruthenium catalysts used in ester hydrogenation.

Pincer ligands have contributed tremendously to environmentally benign, homogeneously catalyzed reductions employing molecular hydrogen as an atom‐economical reducing agent.1d–1f, 16 Particularly, challenging hydrogenations of carboxylic acids and their ester derivatives represent crucial transformations in organic synthesis for both laboratory scale as well as bulk and fine‐chemical industry.^17^ Common synthetic methods often rely on the use of stoichiometric amounts of metal hydrides such as LiAlH_4_ and NaBH_4_,^18^ which is accompanied by the hazard in handling as well as the generation of large amounts of inorganic waste.^19^ In industrial applications, heterogeneous catalysts require harsh reaction conditions (250–350 °C at 100–200 bar) often leading to side‐product formation and limited functional‐group tolerance.^20^ Consequently, there has been a strong drive from both academia and industry to develop molecularly well‐defined homogeneous catalysts for selective catalytic hydrogenations under milder conditions (see representative examples in Figure 2).

Since Milstein's seminal work on the non‐innocent pyridine‐based PNN ligand in Ru‐catalyzed ester hydrogenation (**X**),^21^ a plethora of pincer‐type catalysts has been developed. In contrast to their nonsymmetrical PNN analogue, Ru‐PNP catalysts (**XI** and **XII**) employing symmetrical PNP ligands exhibited significantly less activity in this transformation.^21^, ^22^ This was associated with the lack of hemilability of one of the side arms due to two equally strong electron‐donating phosphorus moieties present in both ligands. As an alternative to the pyridine backbone, aliphatic PN(H)P ligands employed in catalysts such as Ru‐MACHO (**XIII**) but also base‐metal catalysts1e–1h have demonstrated excellent performances in the reduction of esters.^23^ Inspired by the highly active Ru‐SNS (**XIV**) and Ru‐PNN (**XV**) ester hydrogenation catalysts developed by Gusev and co‐workers,^24^ we recently reported on the first reusable resin‐bound Ru‐PNN system (**XVI**) applicable in this reaction under very mild conditions (25 °C, 50 bar).^25^

In this work, we demonstrate the first synthesis of a supported combinatorial library of nonsymmetrical pyridine‐based PNP ligands by using a facile solid‐phase synthesis approach. Moreover, the application of the corresponding heterogeneous Ru‐PNP catalysts in the hydrogenation of various lactones, mono‐, and diesters is reported.

## Results and Discussion

**Scheme 1:**
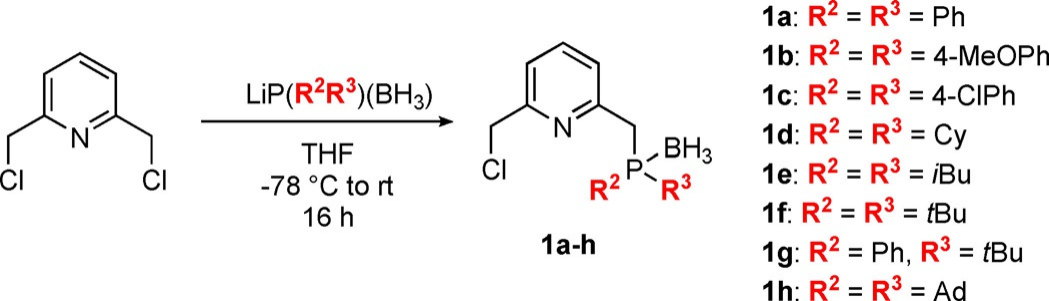
Synthesis of PN building blocks **1 a**–**h**.

The PN building blocks **1 a**–**h** were prepared by adapting a procedure reported by Gargir et al. (Scheme 1).^26^ 2,6‐Bis(chloromethyl)pyridine was treated with 1.0 equivalent of freshly prepared lithium boranyl phosphanides bearing combinations of substituents R^2^ and R^3^ attached to the phosphorus moiety. A series of both aromatic‐ (Ph, 4‐MeOPh, 4‐ClPh, **1 a**–**c**) and alkyl‐based substituents (Cy, *i*Bu, *t*Bu, Ad, **1 d**–**f** and **1 h**) were employed as well as a phosphine–borane with mixed substituents (Ph and *t*Bu, **1 g**).

The systematic variation of R^2^ and R^3^ enables an efficient tuning of the steric and electronic properties of the phosphorus donor atom. Due to the presence of mono‐ and di‐substituted product in the reaction mixture, only low to moderate yields of the desired mono‐substituted phosphine–boranes were obtained. Alternatively, a mixture of mono‐ and disubstituted products was used in the next step because only the desired mono‐substituted PN fragment reacts with the supported reactants whereas the unreacted disubstituted byproduct present in the supernatant solution can easily be filtered off.

**Scheme 2:**
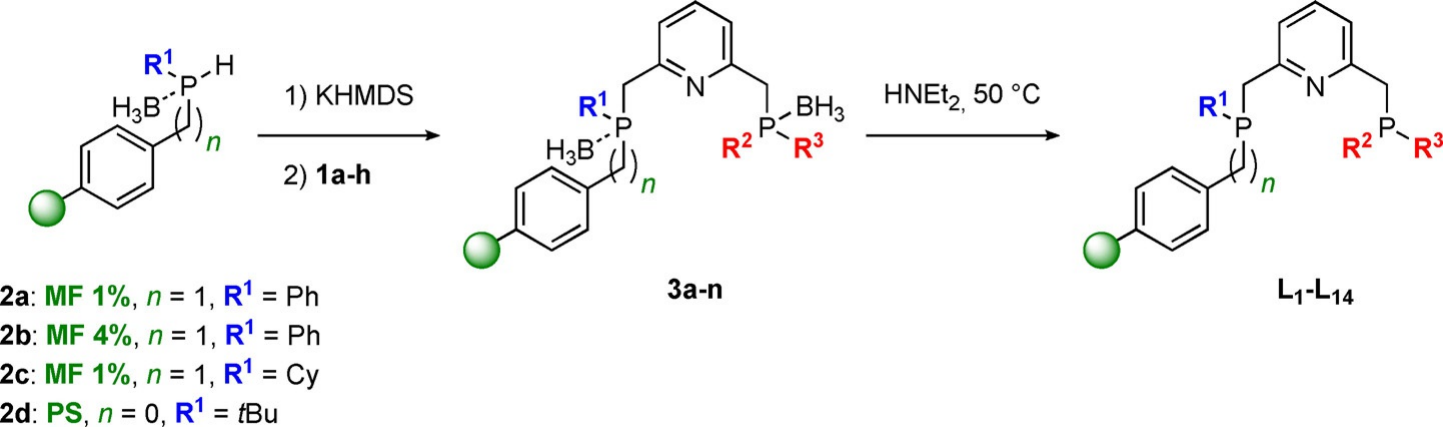
Solid‐phase synthetic approach towards supported pyridine‐based PNP‐type pincer ligands **L_1_**–**L_14_**.

**Figure 3:**
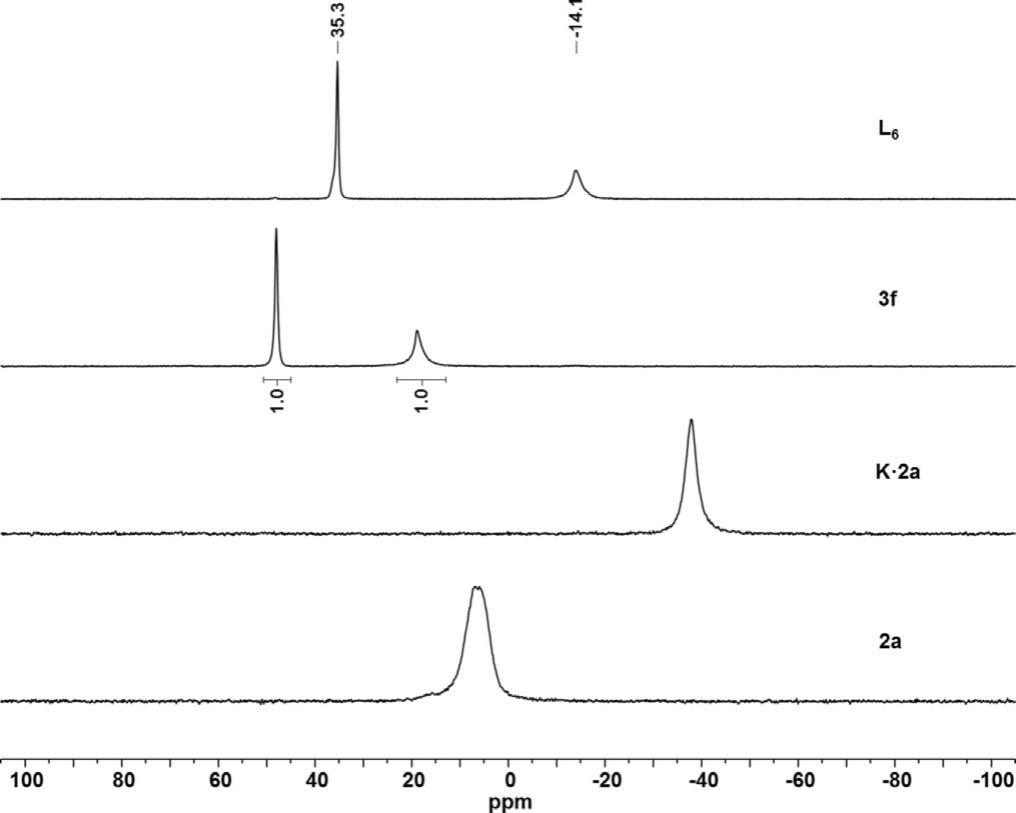
Solid‐phase synthesis of supported PNP pincer ligand **L_6_** monitored by ^31^P NMR.

Next, the secondary phosphine–boranes **2 a**–**d** immobilized on Merrifield resin cross‐linked with 1 % divinylbenzene (DVB, MF 1 %, *n*=1, **2 a** and **2 c**), Merrifield resin cross‐linked with 4 % DVB (MF 4 %, *n*=1, **2 b**) and polystyrene (PS, *n*=0, **2 d**) were prepared as previously reported by our group.^27^ Treatment of **2 a**–**d** with an excess of potassium bis(trimethylsilyl)amide (KHMDS) yielded the deprotonated BH_3_‐protected resin‐bound potassium phosphides **K⋅2 a**–**d** as yellow‐orange resins after one hour (Scheme 2, step 1). Subsequent reaction of **K⋅2 a**–**d** with a slight excess of **1 a**–**h** (1.1 equiv) gave access to the air‐stable immobilized borane‐protected PNP ligands **3 a**–**n** (Scheme 2, step 2). The incorporation of the PN fragment was monitored by gel‐phase ^31^P NMR showing both the quantitative consumption of the potassium phosphide and the appearance of a second resonance in a 1:1 ratio (see Figure 3 for representative synthesis of **3 f**). Although the signal of the first phosphine–borane in close proximity to the support appears very broad, the remote phosphorus moiety shows a significantly sharper signal due to enhanced solution‐like behavior.

After removal of the borane groups by treatment with a large excess of diethylamine at 50 °C, the resin‐bound PNP‐pincer ligands **L_1_**–**L_14_** were obtained. In the presence of more bulky ‐P*t*Bu_2_ and ‐PAd_2_ (Ad=adamantyl) groups, several replacements with fresh diethylamine as well as longer reaction times were required. Quantitative deprotection of both phosphine moieties was readily monitored by ^31^P NMR, indicated by a significant highfield shift of all corresponding phosphorus signals. The representative synthesis of **L_6_** monitored by gel‐phase ^31^P NMR is depicted in Figure 3.

All resin‐bound PNP ligands were synthesized in high yields and purity. Only simple filtration and washing steps were required for purification demonstrating the power of the solid‐phase synthesis approach. Finally, the actual phosphorus loading was determined by elemental analysis.

**Figure 4:**
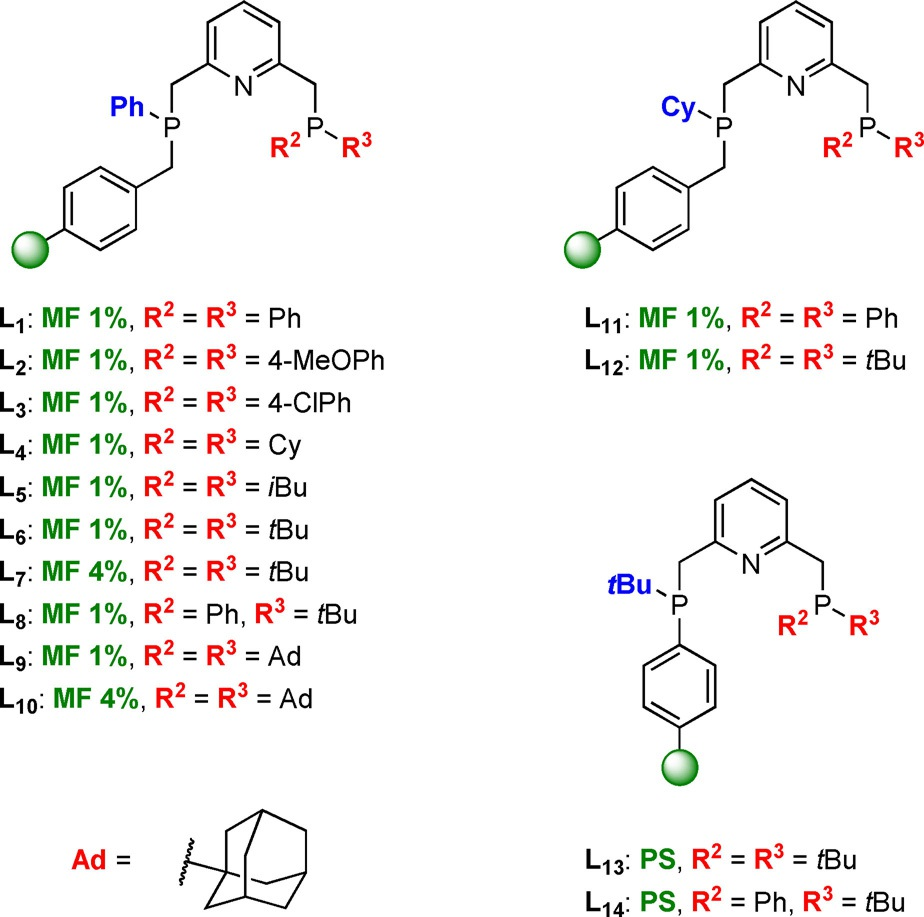
Complete library of supported PNP pincer ligands **L_1_**–**L_14_**.

Through systematic variation of the phosphine substituents R^1^, R^2^, and R^3^ as well as by employing three different types of polymeric supports, a combinatorial library of 14 different supported PNP pincer ligands was efficiently accessed through a solid‐phase synthetic approach (Figure 4). In contrast to structurally similar homogeneous analogues, ligands **L_1_**–**L_13_** represent nonsymmetrical ligands which have been rarely investigated in solution‐phase. However, the combination of two phosphorus moieties exhibiting different electronic and steric properties offers great potential for efficient catalyst tuning. Among all library members, only the nonsymmetrical solution‐phase analogues of **L_6_** and **L_7_** as well as the nearly symmetrical ligand **L_14_** have been reported previously.^5^, ^28^ The ^31^P NMR spectra for both reported examples are well in line with those obtained for their heterogenized equivalents.

**Scheme 3:**
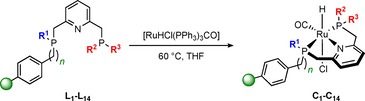
Solid‐phase synthesis of resin‐bound Ru‐PNP complexes **C_1_**–**C_14_**.

In analogy to the synthesis in monophasic systems, the resin‐bound ligands were reacted with the ruthenium precursor [RuHCl(PPh_3_)_3_CO] at 60 °C in THF to afford the corresponding resin‐bound Ru‐PNP complexes **C_1_**–**C_14_** (Scheme 3). The progress of the reaction was monitored by ^31^P NMR indicating full displacement of PPh_3_ together with the quantitative disappearance of the free PNP ligand signals.

**Figure 5:**
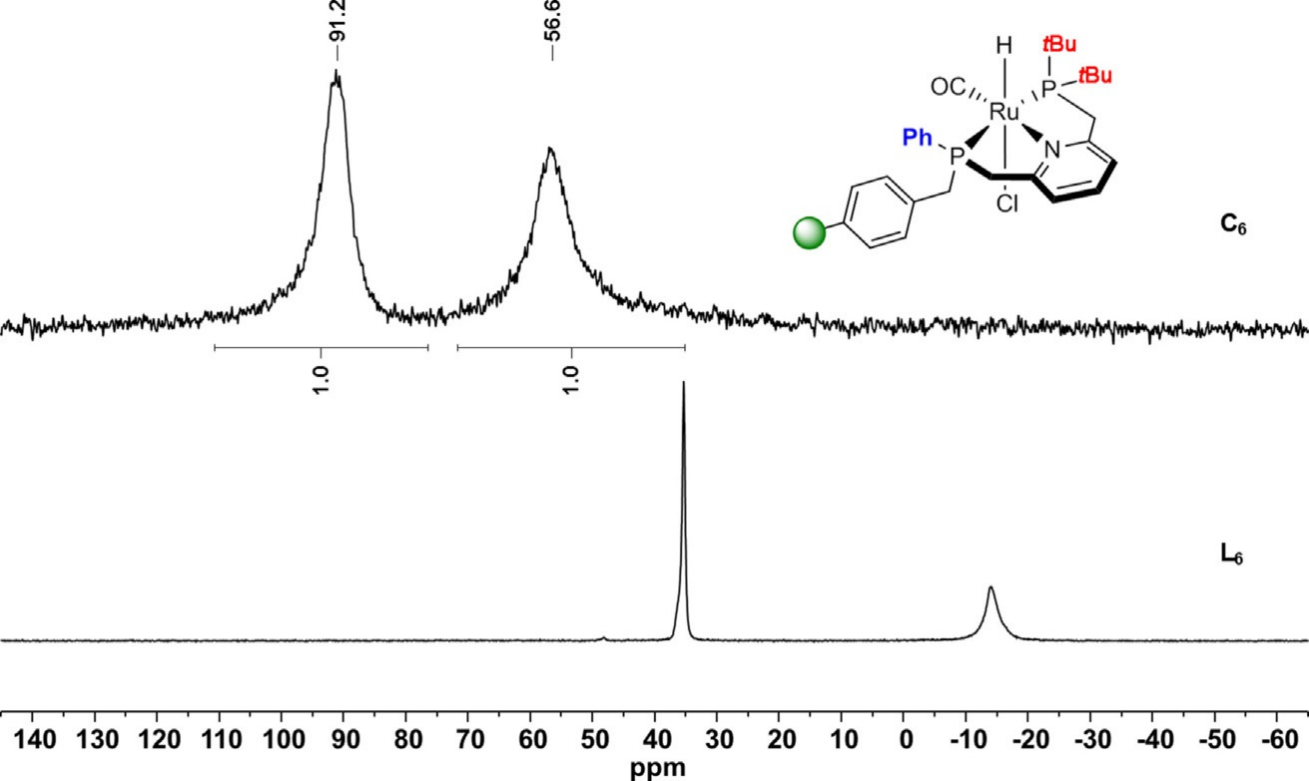
Solid‐phase synthesis of supported Ru‐PNP complex **C_6_** monitored by ^31^P NMR.

The gel‐phase ^31^P NMR spectra of complexes **C_4_**, **C_6_**, **C_7_**, and **C_9_**–**C_12_** reveal two new broad resonances occurring in a 1:1 ratio, which correspond to both phosphine moieties coordinated to the ruthenium center. Due to the lack of solvent‐dependent swelling properties of **C_7_** and **C_10_** immobilized on the higher cross‐linked MF 4 % resin, the signals appear significantly broadened compared with complexes immobilized on supports with 1 % cross‐linking. The representative synthesis of **C_6_** monitored by ^31^P NMR is depicted in Figure 5.

**Figure 6:**
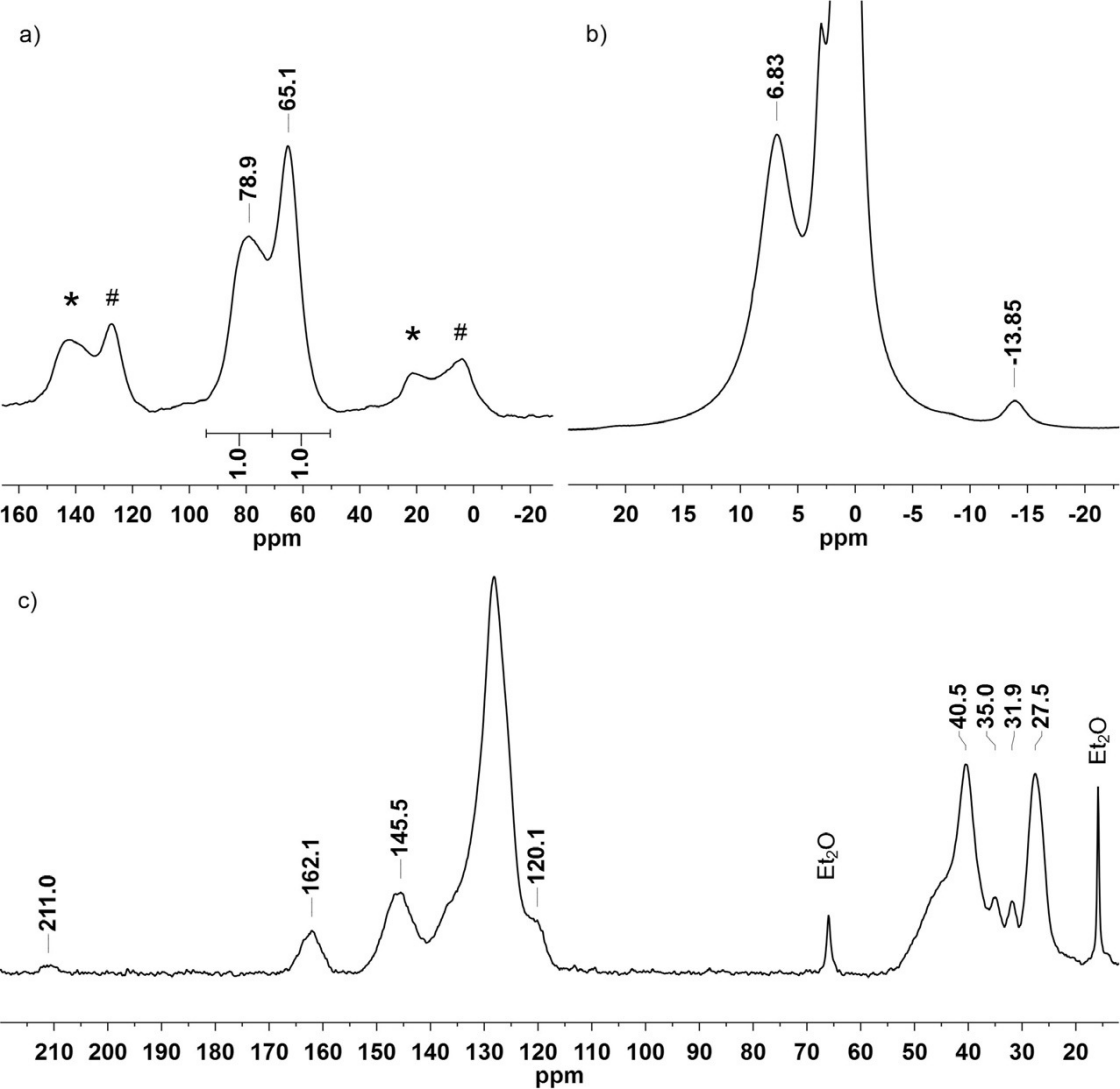
a) ^31^P MAS NMR, b) ^1^H MAS NMR and c) ^13^C CP/MAS NMR spectrum of **C_14_**. Rotational sidebands are denoted by asterisks (*) and (#).

**Figure 7:**
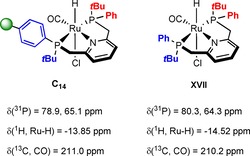
Selected solid‐state (left) and solution‐phase NMR signals (right) of supported Ru‐PNP complex **C_14_** and homogeneous analogue **XVII**.^28^

The signal of the ‐P*t*Bu_2_ group is shifted from *δ*=35.5 in **L_6_** to *δ*=91.2 ppm, whereas the resonance of the resin‐bound ‐PPh moiety arises at *δ*=56.5 in **C_6_** in contrast to *δ*=−13.9 ppm in the free ligand. The phosphine resonances in **C_1_**–**C_3_** and **C_5_** overlap leading to a single broad peak whereas the gel‐phase ^31^P NMR spectra for **C_8_** and **C_13_** reveal three broad signals. The latter can be attributed to the presence of a racemic ‐P(Ph*t*Bu) group in both complexes leading to a difference of up to Δ11–15 ppm between the corresponding signals of the stereoisomers. Due to significant peak broadening in the gel‐phase NMR of **C_14_**, the immobilized complex was analyzed using solid‐state NMR techniques. The ^31^P MAS NMR spectrum shows two signals appearing in a ratio of 1:1 at *δ*=78.9 and *δ*=65.1 ppm corresponding to the two chemically different phosphorus donor atoms (Figure 6 a). The chemical shifts of **C_14_** are in line with those obtained for the homogeneous Ru‐PNP counterpart **XVII** reported by Arenas et al. (Figure 7).^28^

Unfortunately, due to significant peak broadening commonly observed for polymer‐bound complexes, coupling constants could not be determined in solid‐state and gel‐phase NMR.^13^, 27a, 29 Among the broad signals belonging to the aromatic and aliphatic protons of the polymeric backbone, the hydride ligand of **C_14_** was observed at a distinct shift of −13.85 ppm in the ^1^H MAS NMR (Figure 6 b).

In the ^13^C CP/MAS spectrum the CO peak appears at *δ*=211.0 ppm. Characteristic pyridine signals at *δ*=162.1, 145.5, and 120.1 ppm are overlapped by the aromatic signals belonging to the ligand phenyl group as well as to the support (Figure 6 c). Resonances of *t*Bu are observed at *δ*=35.0, 31.9, and 27.5 ppm. The signals corresponding to the methylene side‐arms can be expected at 40.5 ppm but are overlapping with the peaks of the support.

**Scheme 4:**
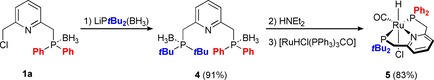
Synthesis of homogeneous Ru‐PNP complex **5**.

To gain additional evidence of the molecular structure of supported complex **C_6_**, the homogeneous Ru‐P^Ph^NP^*t*Bu^ analogue **5** was prepared. Two different phosphorus donor moieties bearing both Ph and *t*Bu substituents are present in the nonsymmetrical PNP pincer ligand. These were introduced by reacting **1 a** with 1.0 equivalent of borane protected lithium di‐*tert*‐butylphosphide leading to the nonsymmetrical borane‐protected PNP ligand **4** in 91 % yield (Scheme 4, step 1).

Removal of the borane groups by treatment with an excess of diethylamine followed by complexation using [RuHCl(PPh_3_)_3_CO] in THF at 60 °C led to **5** in 83 % yield (Scheme 4, steps 2 and 3).

**Figure 8:**
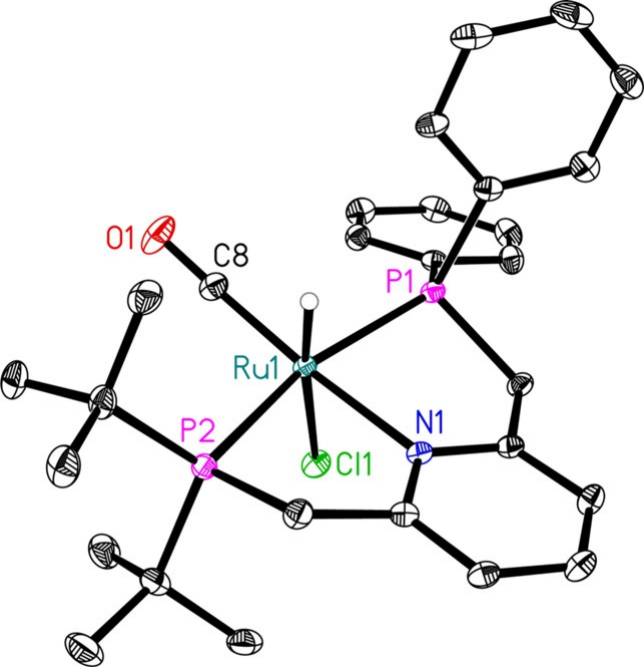
ORTEP representation of **5**. Only one molecule of the asymmetric unit is depicted. Displacement ellipsoids correspond to 30 % probability. C‐bound hydrogen atoms are omitted for clarity. Selected bond lengths (Å) and angles (°) (values of the second molecule of the asymmetric unit are given in square brackets): P1−Ru1=2.3094(6) [2.3175(6)], P2−Ru1=2.3357(6) [2.3494(6)], N1−Ru1=2.1631(16) [2.1514(16)], Cl1−Ru1=2.5183(6) [2.5371(6)], C8−Ru1=1.830(2) [1.826(2)], C8−O1=1.153(3) [1.160(3)]; N1‐Ru1‐P1=80.86(5) [80.41(5)], N1‐Ru1‐P2=81.75(5) [82.14(5)], N1‐Ru1‐C8=172.60(8) [171.45(8)], N1‐Ru1‐Cl1=89.17(4) [87.16(4)], P1‐Ru1‐P2=161.86(2) [160.82(2)].

Single crystals suitable for X‐ray crystallography were obtained by slow diffusion of *n*‐pentane into a solution of **5** in CH_2_Cl_2_. As shown in Figure 8, the complex exhibits a distorted octahedral geometry around the Ru^II^ center with *trans*‐coordination of the CO ligand to the nitrogen atom of pyridine and the hydride *trans* to the chloride. Hence, a meridional coordination geometry of the PNP ligand around the metal center is obtained as reported for symmetrical pyridine‐based [RuHCl(PNP)CO] complexes.^30^

**Figure 9:**
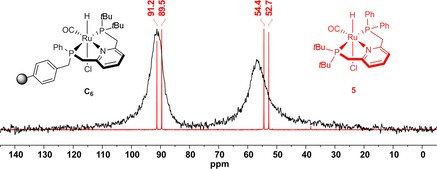
^31^P{^1^H} NMR spectra of supported complex **C_6_** (black) and **5** (red).

The hydride ligand exhibits a doublet of doublets in the ^1^H NMR spectrum at *δ*=−14.51 ppm (*J*_HP_=17.1 and 20.5 Hz) as found in similar Ru‐complexes.^28^, ^31^ The diastereotopic protons of the ‐PPh_2_ methylene arm show signals at *δ*=4.89 ppm (dd, *J*_PH_=9.5, *J*_HH_=16.0 Hz) and at *δ*=4.12 ppm (ddd, *J*_HH_=2.6, *J*_PH_=12.1, *J*_HH_=16.0 Hz). A multiplet at *δ*=3.73–3.66 ppm and a doublet of doublets at *δ*=3.37 ppm (*J*_HH_=8.3, *J*_PH_=16.6 Hz) were observed for both methylene protons belonging to the ‐P*t*Bu_2_ methylene linker. In the ^13^C NMR, the CO ligand appears as a triplet (*J*_PC_=12.2 Hz) at *δ*=208.9 ppm (see the Supporting Information for details). Finally, the ^31^P{^1^H} NMR spectrum of **5** shows two doublets corresponding to the ‐P*t*Bu_2_ (*δ*=90.4 ppm, *J*_PP_=266.6 Hz) and the ‐PPh_2_ entity (*δ*=53.6 ppm, *J*_PP_=266.6 Hz) bound to the central Ru atom (Figure 9, red spectrum). These results compare well to the ^31^P NMR resonances at *δ*=91.2 and 56.5 ppm obtained for the correlating supported Ru‐complex **C_6_** differing only in the methylene linker to the MF support (Figure 9, black spectrum). The CO stretching band in the FT‐IR spectrum of **5** was observed at 1887 cm^−1^.

Subsequently, the supported combinatorial Ru‐PNP library (**C_1_**–**C_14_**) was screened in the hydrogenation of methyl benzoate (**S_1_**). The catalytic reactions were performed under optimized conditions over 16 hours in THF at 80 °C and 50 bar H_2_ pressure. Further reaction conditions are listed in Table S1 (see the Supporting Information). For supported catalyst **C_1_**, bearing phenyl groups on both phosphine moieties of the PNP ligand, 84 % conversion and 92 % selectivity towards the desired benzyl alcohol (BzOH) were obtained (Table 1, entry 1). By changing to more electron‐donating 4‐MeOPh groups bound to the remote phosphine in **C_2_**, an increase in catalyst activity (97 %) and selectivity (99 %) was observed compared with **C_1_** (Table 1, entry 2). Electron‐withdrawing 4‐ClPh groups in **C_3_** led to a reduced activity of 69 % conversion and more transesterification to benzyl benzoate (BzBz, Table 1, entry 3). When changing to unsymmetrical ligands carrying aromatic substituents on the resin‐bound phosphorus‐donor and alkyl substituents on the remote phosphine, moderate activities were obtained for **C_4_** and **C_5_** (Table 1, entries 4 and 5). With increasing steric demand in case of strong electron‐donating *t*Bu groups (**C_6_**) or even more bulky adamantyl groups (**C_9_**), excellent conversions were reached with full selectivity towards BzOH (Table 1, entries 6 and 10). Opposed to the high activity and selectivity at room temperature for the reported resin‐bound Ru‐PNN system (**XVI**),^25^ a reduced temperature of 60 °C resulted in lower conversion of 82 % in case of **C_6_** (Table 1, entry 7). When applying the equivalent catalysts **C_7_** and **C_10_** immobilized on the higher cross‐linked resin MF 4 %, reduced performances (64–65 % conversion, 83–84 % selectivity) compared with **C_6_** and **C_9_** immobilized on MF 1 % were found (Table 1, entries 8 and 11 vs. 6 and 10). This can be attributed to the lack of solvent dependent gel‐like behavior of the higher cross‐linked polymer and the consequently reduced accessibility of the catalytically active sites within the support.

**Table 1:** Ru‐catalyzed hydrogenation of **S_1_** using supported catalysts **C_1_**–**C_14_**.^[a]^

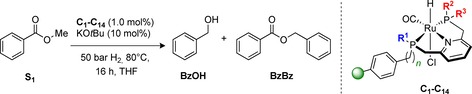						
Entry	Cat.	Substituents			Conversion [%]^[b]^	Selectivity [%]^[c]^
		**R^1^**	**R^2^**	**R^3^**		
1	**C_1_**	Ph	Ph	Ph	84	92
2	**C_2_**	Ph	4‐MeOPh	4‐MeOPh	97	99
3	**C_3_**	Ph	4‐ClPh	4‐ClPh	69	84
4	**C_4_**	Ph	Cy	Cy	61	83
5	**C_5_**	Ph	*i*Bu	*i*Bu	58	86
6	**C_6_**	Ph	*t*Bu	*t*Bu	98	99
7^[d]^	**C_6_**	Ph	*t*Bu	*t*Bu	82	94
8	**C_7_**	Ph	*t*Bu	*t*Bu	64	84
9	**C_8_**	Ph	Ph	*t*Bu	89	96
10	**C_9_**	Ph	Ad	Ad	>99	>99
11	**C_10_**	Ph	Ad	Ad	65	83
12	**C_11_**	Cy	Ph	Ph	72	88
13	**C_12_**	Cy	*t*Bu	*t*Bu	94	98
14	**C_13_**	*t*Bu	*t*Bu	*t*Bu	80	90
15	**C_14_**	*t*Bu	Ph	*t*Bu	70	86
16	**5**	Ph	*t*Bu	*t*Bu	78	94

[a] General conditions: substrate (0.5 mmol), [Ru] (1.0 mol %), KOtBu (10 mol %), THF (1 mL), 80 °C, H_2_ (50 bar), 16 h. [b] Conversion of **S_1_** determined by GC using dodecane as internal standard. [c] Selectivity towards BzOH. [d] 60 °C.

Supported catalyst **C_8_** bearing both a Ph and *t*Bu substituent on the remote phosphine led to 89 % conversion and 96 % selectivity (Table 1, entry 9). Hence, catalytic activity for catalysts with R^1^=Ph rises with gradual increase of steric bulk and electron‐donating properties in the series **C_1_**<**C_8_**<**C_6_**. Replacing R^1^=Ph by a Cy group on the resin‐bound phosphorus donor led to slightly reduced performances for **C_11_** and **C_12_** compared with the corresponding complexes **C_1_** and **C_6_** (Table 1, entries 12 and 13). Catalysts **C_13_** and **C_14_** supported on PS‐resin with R^1^=*t*Bu gave even less activity than their phenyl analogues **C_6_** and **C_8_** (Table 1, entries 14 and 15). Surprisingly, when the nonsymmetrical solution‐phase complex **5** was applied under the same conditions, only 78 % conversion was reached compared with 98 % of its heterogenized counterpart **C_6_** (Table 1, entries 6 and 16). This indicates that the support does not exert a detrimental effect on the performance contrary to reports for many known immobilized catalysts.

**Figure 10:**
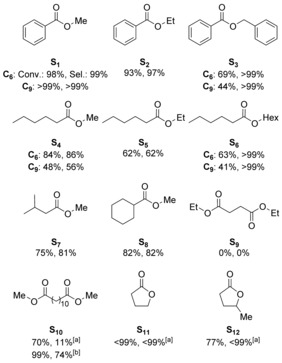
Substrate scope for ester hydrogenation using supported complexes **C_6_** and **C_9_** (conversion and selectivity indicated below structures). For conditions see Table 1, [a] Substrate (0.25 mmol), **C_6_** (1.0 mol %), KO*t*Bu (10 mol %), THF (1 mL), 80 °C, H_2_ (50 bar), 24 h, [b] **C_6_** (2 mol %), 100 °C.

Subsequently, the substrate scope was determined employing the best‐performing supported catalysts **C_6_** and **C_9_** in the hydrogenation of monoesters **S_1_**–**S_8_**, diesters **S_9_**–**S_10_**, and lactones **S_11_**–**S_12_** (Figure 10). Although the aromatic ester ethyl benzoate (**S_2_**) was hydrogenated with slightly reduced conversion and selectivity compared with **S_1_**, benzyl benzoate (**S_3_**) proved to be more challenging (69 % conversion).

When employing catalyst **C_9_**, even less activity (44 %) was observed towards the formation of BzOH. Linear alkyl esters gave up to 84 % conversion and 86 % selectivity to the primary alcohol in case of methyl hexanoate (**S_4_**) whereas ethyl hexanoate (**S_5_**) and hexyl hexanoate (**S_6_**) also proved to be more challenging substrates. Again, better performances were achieved when using **C_6_** instead of **C_9_**. Branched alkyl esters, such as methyl isovalerate (**S_7_**) and methyl cyclohexanoate (**S_8_**), were converted more readily than their linear analogues with 84 % conversion and 86 % selectivity for **S_8_**. As reported for the supported Ru‐PNN catalyst **XVI**, no conversion was observed for diethyl succinate (**S_9_**).^25^ This could be attributed to a chelating coordination of the short‐chain diester **S_9_** to the catalyst. When extending the carbon chain length by using dodecanedioate (**S_10_**), 70 % of the diester was converted after 24 h yielding the monohydrogenated product as the main product whereas only 11 % of the diol was formed. At 2.0 mol % catalyst loading and 100 °C, excellent conversion of **S_10_** was obtained with 74 % selectivity towards the desired 1,12‐dodecanediol. Finally, γ‐butyrolactone (**S_11_**) and biomass‐derived γ‐valerolactone (**S_12_**) were selectively converted into the corresponding diols underlining the versatility of the heterogenized Ru‐PNP system.

Finally, the recovery and recyclability of the supported Ru‐PNP catalyst **C_6_** was investigated in the hydrogenation of **S_1_** (Table 2). It was decided to shorten the reaction time from 16 to 2 h to assess any effect on the catalyst performance as a consequence of catalyst deactivation. After each cycle, the supernatant solution was filtered off followed by addition of fresh substrate and base stock solution to start a new catalytic run. The results show that the heterogenized catalyst was successfully recovered and reused up to at least 4 times. In run 2 and 3, a small decrease in activity of 4 % together with a slight drop in selectivity was observed compared with run 1. After run 5, the catalyst reached 33 % conversion and 68 % selectivity indicating some catalyst decomposition. This could be attributed to deterioration of the polymeric support due to mechanical stirring leading to finely ground particles the supernatant solution. The loss of activity cannot be explained by Ru leaching, because the Ru content of the supernatant was below the detection limit of 5 ppm after tenfold dilution. This indicates that no more than 10 % of the Ru content could be lost by leaching, less than the loss of activity during the recycle experiments. However, these preliminary results demonstrate the potential for recovery and recycling of supported Ru‐PNP catalysts only requiring simple filtrations. Given that continuous flow processes in fixed‐bed reactors offer the opportunity to recycle under constant conditions without disruption of the catalytic system, these immobilized catalysts represent highly suitable candidates for application under flow conditions.29b

**Table 2:** Batch recycling using **C_6_** in the hydrogenation of **S_1_**.

Entry	Conversion [%]^[b]^	Selectivity [%]^[c]^
1	44	77
2	40	75
3	40	74
4	36	70
5	33	68

[a] Conditions: substrate (0.5 mmol), **C_6_** (1.0 mol %), KO^*t*^Bu (10 mol %), THF (1 mL), 100 °C, H_2_ (50 bar), 2 h. [b] Conversion of **S_1_** determined by GC using dodecane as internal standard. [c] Selectivity towards BzOH.

## Conclusions

We developed the first facile access to a combinatorial library of nonsymmetrical resin‐bound PNP pincer‐type ligands by employing a solid‐phase synthesis approach. Systematic variation of phosphine substituents combined with the use of three different types of polymeric supports led to a library with 14 members (**L_1_**–**L_14_**). The supported ligands were obtained in high purity only requiring minimal purification steps opposed to typically arduous synthetic protocols for solution‐phase analogues. The immobilized nonsymmetrical PNP ligands offer higher potential for efficient fine‐tuning of stereo‐electronic ligand properties compared with *C*_2*v*_ symmetrical ligands. The corresponding resin‐bound Ru‐PNP complexes **C_1_**–**C_14_** were successfully screened in the hydrogenation of methyl benzoate (**S_1_**) under mild conditions. Minor changes within the structure of the phosphine substituents had a substantial impact on catalyst performances underlining the necessity of catalyst screening. A broad range of monoesters and long‐chain diester **S_10_** were hydrogenated to the desired alcohols under mild conditions. Lactones, such as bioderived γ‐valerolactone (**S_12_**), could be readily converted with high selectivities towards the corresponding diols. Preliminary recycling experiments indicated facile recovery and reusability of the supported catalyst.

## Experimental Section

### General procedure for the synthesis of 1 a–g

To a solution of secondary phosphine‐borane adduct (1.0 equiv) in dry THF at −78 °C, *n*BuLi (2.5 m in hexanes, 1.0 equiv) or *sec*‐BuLi (1.4 m in cyclohexane, 1.0 equiv) in case of (adamantyl)_2_PH⋅BH_3_ was added dropwise. The solution was stirred for 30 min at −78 °C and subsequently warmed to room temperature and was left for an additional amount of time until full conversion was achieved according to ^31^P NMR. 2,6‐Bis(chloromethyl)pyridine (1.0 equiv) was dissolved in dry THF and cooled to −78 °C. Next, the freshly prepared lithium boranyl phosphanide solution (0.28 m, 1.0 equiv) in THF was added slowly. The mixture was warmed up to room temperature overnight leading to a pale‐yellow solution. The solvent was removed under vacuum and the yellow residue was dissolved in CH_2_Cl_2_. The organic phase was washed with water and brine and subsequently dried over MgSO_4_. After filtration, the solvent was removed under reduced pressure. The residue was purified through flash chromatography (9:1 hexanes/ethyl acetate) or as stated otherwise yielding a white solid.

### General procedure for the synthesis of resin‐bound PNP‐pincer ligands L_1_–L_14_

Step 1: A resin‐bound phosphine‐borane (**2 a**, 1.40 g, 1.57 mmol, 1.0 equiv), (**2 b**, 0.22 g, 0.24 mmol, 1.0 equiv), (**2 c**, 0.25 g, 0.28 mmol, 1.0 equiv), or (**2 d**, 0.12 g, 0.22 mmol, 1.0 equiv) was swollen in THF (20 mL). After addition of KHMDS (20 % in THF, 10 equiv) under gentle stirring to avoid mechanical abrasion of the resin, the orange resin was allowed to react for 2 hours at room temperature. The supernatant was removed and the resin was washed three times with THF (15 mL) followed by three times with Et_2_O (15 mL). Without further purification, the BH_3_‐protected resin‐bound potassium phosphides were used in the next step.

Step 2: A previously synthesized BH_3_‐protected resin‐bound potassium phosphide (**K⋅2 a**, 1.57 mmol, 1.0 equiv), (**K⋅2 b**, 0.24 mmol, 1.0 equiv), (**K⋅2 c**, 0.28 mmol, 1.0 equiv), or (**K⋅2 d**, 0.22 mmol, 1.0 equiv) was swollen in THF (10 mL) and cooled to −78 °C. A 2‐(chloromethyl)‐6‐(phosphinomethyl)pyridine‐borane (**1 a**–**h**,1.1 equiv) was azeotropically dried with toluene (3×5 mL), dissolved in THF (10 mL) and added to the resin at −78 °C under gentle stirring to avoid mechanical abrasion. The mixture was left with occasional stirring and allowed to warm up to room temperature overnight. The reaction was monitored using gel‐phase ^31^P NMR and was allowed to react until full conversion was observed. Next, the supernatant was removed and the resin was washed three times with THF (10 mL) followed by three times with Et_2_O (10 mL) and dried in vacuo yielding a pale yellow resin‐bound PNP borane adduct (**3 a**–**n**).

Step 3: A resin‐bound PNP borane adduct **3 a**–**n** synthesized in the last step was swollen in 10 mL of diethyl amine and heated to 50 °C overnight with occasional stirring to avoid mechanical abrasion of the resin. The reaction was monitored using gel‐phase ^31^P NMR and was allowed to react until full conversion was observed. Next, the mixture was cooled to room temperature and the supernatant was removed. The resin was washed with three portions of THF (10 mL) followed by three portions of Et_2_O (10 mL) and dried in vacuo yielding a pale yellow resin‐bound PNP pincer ligand (**L_1_**–**L_14_**).

### General procedure for the synthesis of resin‐bound complexes C_1_–C_14_

A previously synthesized resin‐bound PNP pincer ligand (**L_1_**–**L_14_**, ≈80–170 mg, 1.0 equiv) and [Ru(HCl(PPh_3_)_3_CO] (1.1 equiv) were weighed into a Schlenk tube. The mixture was suspended in THF (10 mL) and heated to 60 °C under gentle stirring. The reaction mixture was left at 60 °C with occasional stirring to avoid mechanical abrasion of the resin and the progress of the reaction was monitored by gel‐phase ^31^P NMR. Once full complexation of the resin‐bound PNP ligand was observed, the mixture was cooled to room temperature and the supernatant was removed. The resin‐bound complex was washed with three portions of THF (10 mL), three portions of CH_2_Cl_2_ (10 mL) followed by three portions of Et_2_O (10 mL). After drying in vacuo, a yellow to brown resin‐bound Ru‐PNP complex (**C_1_**–**C_14_**) was obtained.

### General procedure for Ru‐catalyzed ester hydrogenation

The hydrogenation experiments were performed in a stainless steel autoclave charged with an insert suitable for up to 12 reaction vessels (2 mL) including Teflon mini stir bars. Inside a glove box, a reaction vessel was charged with a resin‐bound Ru‐PNP complex **C_1_**–**C_14_** (≈7 mg, 5.0 μmol, 1.0 mol %). To the reaction vessel 0.5 mL of a stock solution of KO*t*Bu (10 mol %) in THF was added and the mixture was stirred for 5 minutes. Next, 0.5 mL of the substrates **S_1_**–**S_12_** (0.5 mmol) and the internal standard dodecane (50 mol %) dissolved in THF were added. Subsequently, the autoclave was purged three times with 10 bar of argon gas and the insert loaded with reaction vessels was transferred into the autoclave. Next, the autoclave was purged three times with 10 bar of H_2_ and then pressurized (30–50 bar) and heated to the desired temperature (40–100 °C). The reaction mixtures were gently stirred at 450 rpm for 16–24 hours. The autoclave was cooled to room temperature, depressurized and the conversion was determined by GC‐FID.

## Conflict of interest

The authors declare no conflict of interest.

## Supporting information

As a service to our authors and readers, this journal provides supporting information supplied by the authors. Such materials are peer reviewed and may be re‐organized for online delivery, but are not copy‐edited or typeset. Technical support issues arising from supporting information (other than missing files) should be addressed to the authors.

Supplementary
