# Supplementary material for: Preparation of a Series of Supported Nonsymmetrical PNP‐Pincer Ligands and the Application in Ester Hydrogenation
Source: Chemistry. 2019 Nov 4;25(67):15341–50. doi: 10.1002/chem.201903379 (PMC6916561; doi:10.1002/chem.201903379)
Supplement: Supplementary file 1 — Supplementary [file CHEM-25-15341-s001.pdf]

CHEMISTRY
A European Journal

Supporting Information

Preparation of a Series of Supported Nonsymmetrical PNP-Pincer Ligands and the Application in Ester Hydrogenation

Robert Konrath,[a, b] Anke Spannenberg,[b] and Paul C. J. Kamer*[b]

chem_201903379_sm_miscellaneous_information.pdf

Supporting Information

Table of Contents

General Experimental 2
General Procedure for the Synthesis of **1a-g** 3
General Procedure for the Synthesis of Resin-Bound PNP-Pincer Ligands **L_1-L_{14}** 9
General Procedure for the Synthesis of Resin-Bound complexes **C_1-C_{14}** 13
Synthesis of PNP-Borane Adduct **4** 16
Synthesis of homogeneous complex **5** 17
General Procedure for Ru-catalyzed Ester Hydrogenation 18
General Procedure for Batch Recycling Experiments 20
NMR Spectra 20
IR Spectra 53
Representative GC-Traces of Ester Hydrogenation Experiments 55
X-Ray Crystallographic Data of **5** 60
References 61

General Experimental

All reactions and manipulations were carried out using standard Schlenk techniques under inert atmosphere of purified argon or in an MBraun glovebox unless stated otherwise. All glassware was dried prior to use to remove traces of water. All chemicals were obtained from commercial suppliers and were used as received unless otherwise stated. Diethyl ether and THF were distilled from sodium/benzophenone and toluene was distilled from sodium. Distilled THF used in catalytic reactions was additionally dried over 3 Å molecular sieves for a minimum of 72 h. CH_2Cl_2 and diethylamine were distilled from calcium hydride. C_6D_6 was thoroughly degassed with Argon and stored over 4 Å molecular sieves. Novabiochem™ Merrifield resin (100-200 mesh, 1.23 mmol·g^{-1}, 1% cross-linked) was obtained from EMD Millipore. ParaMax Merrifield resin (100-200 mesh, 1.2 mmol·g^{-1}, 4% cross-linked) was obtained from Advanced Chemtech. Supported secondary phosphines **2a-d** were synthesized according to literature.[1] The secondary phosphine-boranes $^{i}Bu_2PH(BH_3)$, $Ad_2PH(BH_3)$ and $Ph^{t}BuPH(BH_3)$ were synthesized starting from their corresponding secondary phosphines.[2] The syntheses of $(4\text{-MeOPh})_2PH(BH_3)$ and $(4\text{-ClPh})_2PH(BH_3)$ were adapted from literature procedure.[3]

NMR spectroscopic analysis was conducted using a Bruker FOURIER 300, an AVANCE II 400 or an AVANCE III 500. ^{1}H, ^{31}P and ^{13}C NMR experiments were recorded using standard NMR techniques and the chemical shifts (δ) are reported relative to the solvent peak. Gel-phase ^{31}P NMR spectra of all resins were recorded unlocked and without additional shimming in dry THF as a solvent unless mentioned otherwise. Chemical shifts are reported relative to 85% H_3PO_4 in water. Solid-state NMR spectra were acquired using Bruker Avance III spectrometers equipped with a 9.4 T widebore superconducting magnet operating at Larmor frequencies of 400.1 MHz for ^{1}H, 161.9 MHz for ^{31}P and 100.6 MHz for ^{13}C. Samples were packed in 4.0 mm ZrO_2 rotors and rotated at MAS rates of 14 kHz (^{1}H), 12.5 kHz (^{13}C) and 10 kHz (^{31}P). ^{13}C spectra were acquired using cross-polarization (CP), with a contact pulse (ramped for ^{1}H) between 1 and 5 ms (^{13}C) duration. Multiplicities are provided using the following abbreviations: s = singlet, d = doublet, t = triplet, m = multiplet and br = broad and the couplings (*J*) are reported in Hz. NMR spectra were processed using TopSpin 3.2 or MestReNova 11.0. IR spectra were recorded on a Shimadzu IRAffinity-1S spectrometer as KBr disks or on a Bruker Alpha FT-IR spectrometer as solids. Elemental analyses were measured by Mikroanalytisches Laboratorium Kolbe in Oberhausen, Germany. GC measurements were performed on a Thermo Trace GC ultra, see further experimental details for columns and conditions. ICP-OES analyses were measured using a Varian 715-ES.

General Procedure for the Synthesis of 1a-g

To a solution of secondary phosphine-borane adduct (1.0 equiv.) in dry THF at -78 °C, *n*-BuLi (2.5 M in hexanes, 1.0 equiv.) or *sec*-BuLi (1.4 M in cyclohexane, 1.0 equiv.) in case of (adamantyl)$_2$PH·BH$_3$ was added dropwise. The solution was stirred for 30 min at -78 °C and subsequently warmed to room temperature and was left for an additional amount of time until full conversion was achieved according to ^{31}P NMR. 2,6-bis(chloromethyl)pyridine (1.0 equiv.) was dissolved in dry THF and cooled to -78 °C. Next, the freshly prepared lithium boranyl phosphanide solution (0.28 M, 1.0 equiv.) in THF was added slowly. The mixture was warmed up to room temperature overnight leading to a pale yellow solution. The solvent was removed under vacuum and the yellow residue was dissolved in CH_2Cl_2. The organic phase was washed with water and brine and subsequently dried over $MgSO_4$. After filtration, the solvent was removed under reduced pressure. The residue was purified *via* flash chromatography (9:1 Hexanes : EtOAc) or as stated otherwise yielding a white solid.

*2-(Chloromethyl)-6-((di-phenylphosphino)-methyl)pyridine-borane Adduct (**1a**)*

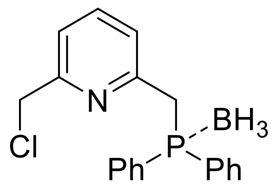

1a

The phosphine-borane adduct **1a** was obtained from 2,6-bis(chloromethyl)pyridine (2.0 g, 11.4 mmol, 1.0 equiv.) and lithium boranyl-diphenyl-phosphanide (1.0 equiv.) as a white solid after flash chromatography. Yield: 1.40 g (36%). ^{1}H NMR (300 MHz, $CDCl_3$): δ = 7.76-7.69 (m, 4H, PPh), 7.58 (t, 1H, J_{HH} = 7.7 Hz, pyridine-H), 7.50-7.37 (m, 6H, PPh), 7.26-7.20 (m, 2H, pyridine-H), 4.46 (s, 2H, CH_2Cl), 3.86 (d, 2H, J_{PH} = 12.0 Hz, CH_2P), 1.01 (br, 3H, BH_3) ppm, ^{13}C NMR (101 MHz, $CDCl_3$): δ = 155.8 (s, pyridine-*C*-CH_2Cl), 153.1 (d, J_{PC} = 4.5 Hz, pyridine-*C*-CH_2P), 137.1 (s, pyridine-CH), 132.8 (d, J_{PC} = 9.3 Hz, 4xAr-CH), 131.3 (d, J_{PC} = 2.1 Hz, 2xAr-CH), 128.6 (d, J_{PC} = 55.3 Hz, 2xAr-C-P), 128.6 (d, J_{PC} = 10.1 Hz, 4xAr-CH), 124.4 (d, J_{PC} = 3.3 Hz, pyridine-CH), 120.8 (d, J_{PC} = 2.1 Hz, pyridine-CH), 46.5 (s, CH_2Cl), 36.5 (d, J_{PC} = 31.5 Hz, CH_2P) ppm. ^{31}P NMR (162 MHz, $CDCl_3$): δ = 18.1 (m) ppm; IR (solid): $\tilde{\nu}$ = 3054 (w), 2401 (w), 2367 (m, B–H), 1584 (m), 1456 (m), 1433 (m), 1105 (m), 1061 (m), 821 (w), 740 (s, P-C), 690 (s), 596 (m), 532 (m), 493 (m), 464 (m), 418 (m) cm^{-1}; ESI-HRMS (m/z, pos): Calculated for $C_{19}H_{20}BClNP$-H: 338.1037 [*M*-H]$^+$; found: 338.1042;

Elemental analysis calcd (%) for [$C_{19}H_{20}BClNP$]: C 67.20, H 5.94, N 4.12; found: C 67.73, H 5.93, N 4.02.

2-(Chloromethyl)-6-(bis(4-methoxy)-phenylphosphino)-methyl)pyridine-borane Adduct ***1b***

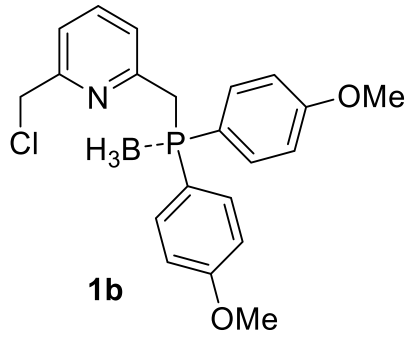

1b

The phosphine-borane adduct **1b** was obtained from 2,6-bis(chloromethyl)pyridine (0.24 g, 1.4 mmol, 1.0 equiv.) and lithium boranyl-bis(4-methoxy)phenyl-phosphanide (1.0 equiv.) as a white solid after flash chromatography. Yield: 0.30 g (53%). ^{1}H NMR (400 MHz, $CDCl_3$): δ = 7.65-7.58 (m, 4H, Ar-H), 7.55 (t, 1H, J_{HH} = 7.7 Hz, pyridine-H), 7.24-7.22 (m, 1H, pyridine-H) 7.18-7.16 (m, 1H, pyridine-H), 6.93-6.90 (m, 4H, Ar-H), 4.46 (s, 2H, CH_2Cl), 3.82 (s, 6H, OCH_3), 3.76 (d, 2H, J_{PH} = 11.9 Hz, CH_2P), 1.06 (br, 3H, BH_3) ppm. ^{13}C NMR (101 MHz, $CDCl_3$): δ = 161.9 (d, J_{PC} = 2.3 Hz 2x*C*-OCH_3), 155.7 (s, pyridine-*C*-CH_2Cl), 153.5 (d, J_{PC} = 4.7 Hz, pyridine-*C*-CH_2P), 137.0 (s, pyridine-CH), 134.4 (d, J_{PC} = 10.5 Hz, 4xAr-CH), 124.4 (d, J_{PC} = 3.2 Hz, pyridine-CH), 120.8 (d, J_{PC} = 2.0 Hz, pyridine-CH), 119.5 (d, J_{PC} = 60.3 Hz, 2xAr-C-P), 114.2 (d, J_{PC} = 11.0 Hz, 4xAr-CH), 55.3 (s, 2xOCH_3), 46.6 (s, CH_2Cl), 37.1 (d, J_{PC} = 32.0 Hz, CH_2P) ppm. ^{31}P NMR (162 MHz, $CDCl_3$): δ = 15.6 (m) ppm; IR (solid): $\tilde{\nu}$ = 2962 (w), 2903 (m), 2841 (w), 2382 (m, B–H), 2345 (w), 1593 (m), 1569 (m), 1500 (m), 1452 (m), 1410 (w), 1293 (m), 1250 (s), 1180 (m), 1109 (m), 1064 (m), 1022 (m), 819 (s), 807 (s), 765 (m), 740 (m), 689 (w), 619 (m), 587 (m), 525 (s) cm^{-1}; ESI-HRMS (m/z, pos): Calculated for $C_{21}H_{24}BClNO_2P$-H: 398.1248 [*M*-H]$^+$; found: 398.1252; Elemental analysis calcd (%) for [$C_{21}H_{24}BClNO_2P$]: C 63.11, H 6.05, N 3.50; found: C 63.59, H 6.08, N 3.37.

2-(Chloromethyl)-6-(bis(4-chloro)-phenylphosphino)-methyl)pyridine-borane Adduct ***1c***

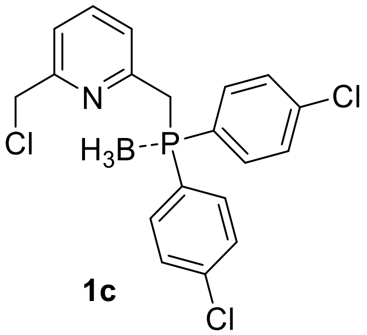

1c

The phosphine-borane adduct **1c** was obtained from 2,6-bis(chloromethyl)pyridine (0.24 g, 1.4 mmol, 1.0 equiv.) and lithium boranyl-bis(4-chloro)phenyl-phosphanide (1.0 equiv.) as an off-white solid after flash chromatography (CH_2Cl_2). Yield: 65.3 mg (16%). ^1H NMR (300 MHz, $CDCl_3$): δ = 7.67-7.61(m, 4H, 4xAr-H), 7.58 (t, 1H, J_{HH} = 7.2 Hz, pyridine-H), 7.42-7.38 (m, 4H, Ar-H) 7.26-7.23 (m, 1H, pyridine-H), 7.19-7.15 (m, 1H, pyridine-H), 4.45 (s, 2H, CH_2Cl), 3.80 (d, 2H, J_{PH} = 11.8 Hz, CH_2P), 1.07 (br, 3H, BH_3) ppm. ^{13}C NMR (75 MHz, $CDCl_3$): δ = 156.1 (s, pyridine-*C*-CH_2Cl), 152.7 (s, pyridine-*C*-CH_2P), 138.4 (s, 2x*C*-Cl), 137.5 (s, pyridine-CH), 134.2 (d, J_{PC} = 10.2 Hz, 4xAr-CH), 129.2 (d, J_{PC} = 10.6 Hz, 4xAr-CH), 126.7 (d, J_{PC} = 55.7 Hz, 2xAr-C-P) 124.6 (s, pyridine-CH), 121.3 (s, pyridine-CH), 46.5 (s, CH_2Cl), 36.4 (d, J_{PC} = 31.3 Hz, CH_2P) ppm. ^{31}P NMR (121 MHz, $CDCl_3$): δ = 18.3 (m) ppm; IR (solid): $\tilde{\nu}$ = 2962 (w), 2379 (m, B–H), 2347 (m), 1574 (m), 1482 (m), 1451 (m), 1388 (m), 1297 (w), 1262 (w), 1083 (s), 1058 (s), 1012 (m), 813 (s), 768 (s), 744 (s), 701 (m), 615 (s), 543 (m), 489 (s) cm^{-1}; ESI-HRMS (m/z, pos): Calculated for $C_{19}H_{18}BCl_3NP$-H: 406.0257 [*M*-H]$^+$; found: 406.0261; Elemental analysis calcd (%) for [$C_{19}H_{18}BCl_3NP$]: C 55.87, H 4.44, N 3.43; found: C 55.46, H 4.26, N 3.05.

2-(Chloromethyl)-6-((di-cyclohexylphosphino)-methyl)pyridine-borane Adduct ***1d***

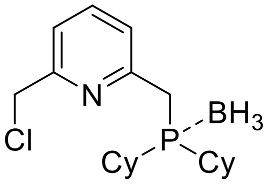

1d

The phosphine-borane adduct **1d** was obtained from 2,6-bis(chloromethyl)pyridine (0.64 g, 3.6 mmol, 1.0 equiv.) and lithium boranyl-di-cyclohexyl-phosphanide (1.0 equiv.) as an clear oil after flash chromatography. Yield: 0,25 g (20%). ^1H NMR (300 MHz, $CDCl_3$): δ = 7.64 (t, 1H, J_{HH} = 7.7 Hz, pyridine-H), 7.31-7.26 (m, 2H, pyridine-H), 4.60 (s, 2H, CH_2Cl), 3.22 (d, 2H,

J_{PH} = 11.2 Hz, CH_2P), 1.87-1.77 (m, 12H, Cy-H), 1.38-1.17 (m, 10H, Cy-H), 0.34 (br, 3H, BH_3) ppm. ^{13}C NMR (101 MHz, $CDCl_3$): δ = 156.0 (d, J_{PC} = 1.6 Hz, pyridine-*C*-CH_2Cl), 155.1 (d, J_{PC} = 5.6 Hz, pyridine-*C*-CH_2P), 137.4 (d, J_{PC} = 1.8 Hz, pyridine-CH), 124.5 (d, J_{PC} = 3.1 Hz, pyridine-CH), 120.9 (d, J_{PC} = 2.0 Hz, pyridine-CH), 46.9 (s, CH_2Cl), 35.3 (d, J_{PC} = 5.0 Hz, CH_2P), 31.7 (d, J_{PC} = 31.1 Hz, PCH_2), 30.4 (d, J_{PC} = 26.4 Hz, CH_2PCH), 27.1 (d, J_{PC} = 4.4 Hz, Cy-CH_2), 27.0 (d, J_{PC} = 5.1 Hz, Cy-CH_2), 26.9 (s, Cy-CH_2), 26.8 (d, J_{PC} = 2.1 Hz, Cy-CH_2), 26.1 (d, J_{PC} = 1.5 Hz, Cy-CH_2) ppm. ^{31}P NMR (121 MHz, $CDCl_3$): δ = 28.4 (m) ppm; IR (solid): $\tilde{\nu}$ = 2926 (s), 2851 (s), 2368 (s, B–H), 1591 (m), 1574 (m), 1451 (s), 1404 (w), 1274 (w), 1062 (s), 1004 (w), 995 (w), 891 (w), 855 (s), 827 (m), 745 (s), 595 (m), 579 (m), 525 (w) cm^{-1}; ESI-HRMS (m/z, pos): Calculated for $C_{19}H_{32}BClNP$-H: 350.1976 [*M*-H]$^+$; found: 350.1979 [M-H]$^+$; Elemental analysis calcd (%) for [$C_{19}H_{32}BClNP$]: C 64.89, H 9.17, N 3.98; found: C 64.83, H 9.04, N 3.98.

2-(Chloromethyl)-6-((di-iso-butylphosphino)-methyl)pyridine-borane Adduct ***1e***

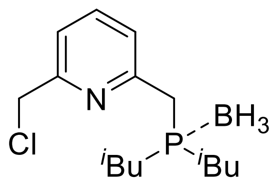

1e

The phosphine-borane adduct **1e** was obtained from 2,6-bis(chloromethyl)pyridine (0.29 g, 1.7 mmol, 1.0 equiv.) and lithium boranyl-di-*iso*-butyl-phosphanide (1.0 equiv.) as a white solid after flash chromatography. Yield: 0.10 g (20%). ^{1}H NMR (500 MHz, $CDCl_3$): δ = 7.67 (t, 1H, J_{HH} = 7.7 Hz, pyridine-H), 7.33 (d, 1H, J_{HH} = 7.7 Hz, pyridine-H), 7.19 (d, 1H, J_{HH} = 7.7 Hz, pyridine-H), 4.62 (s, 2H, CH_2Cl), 3.22 (d, 2H, J_{PH} = 10.5 Hz, CH_2P), 2.02 (m, 2H, C*H*($CH_3)_2$), 1.67 (ddd, 2H, J_{HH} = 14.7, 12.0, 7.0 Hz, C*H*HCH($CH_3)_2$), 1.48 (ddd, 2H, J_{HH} = 14.7, 10.1, 7.0 Hz, CH*H*CH($CH_3)_2$), 1.01 (dd, 12H, J_{HH} = 12.0, 6.6 Hz, 4xCH_3), 0.54 (br, 3H, BH_3) ppm. ^{13}C NMR (126 MHz, $CDCl_3$): δ = 156.1 (s, pyridine-*C*-CH_2Cl), 154.4 (d, J_{PC} = 7.3 Hz, pyridine-*C*-CH_2P), 137.5 (s, pyridine-CH), 124.1 (d, J_{PC} = 3.3 Hz, pyridine-CH), 120.9 (s, pyridine-CH), 46.7 (s, CH_2Cl), 35.3 (d, J_{PC} = 5.0 Hz, CH_2P), 33.1 (d, J_{PC} = 31.2 Hz, PCH_2), 24.8 (dd, J_{PC} = 18.1, 7.3 Hz, CH_3), 24.3 (s, *C*($CH_3)_2$) ppm. ^{31}P NMR (202 MHz, $CDCl_3$): δ = 15.9 (m) ppm; IR (solid): $\tilde{\nu}$ = 2958 (m), 2926 (w), 2872 (w), 2380 (s, B–H), 1583 (m), 1456 (s), 1403 (w), 1282 (w), 1250 (w), 1139 (w), 1056 (s), 993 (w), 826 (s), 749 (s), 711 (w), 631 (w), 574 (m), 520 (w) cm^{-1}; ESI-HRMS (m/z, pos): Calculated for $C_{15}H_{28}BClNP$-H: 298.1663 [*M*-H]$^+$; found: 298.1664; Elemental analysis calcd (%) for [$C_{15}H_{28}BClNP$]: C 60.13, H 9.42, N 4.67; found: C 60.41, H 9.59, N 4.59.

2-(Chloromethyl)-6-((di-tert-butylphosphino)-methyl)pyridine-borane Adduct ***1f***

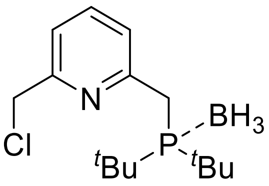

1f

The phosphine-borane adduct **1f** was obtained from 2,6-bis(chloromethyl)pyridine (1.0 g, 5.7 mmol, 1.0 equiv.) and lithium boranyl-di-*tert*-butyl-phosphanide (1.0 equiv.) as a white solid after flash chromatography. Yield: 0.97 g (57%). NMR data matches literature values.[4] ^{1}H NMR (400 MHz, $CDCl_3$): δ = 7.67 (t, 1H, J_{HH} = 7.7 Hz, pyridine-H), 7.58 (d, 1H, J_{HH} = 7.7 Hz, pyridine-H), 7.31 (d, 1H, J_{HH} = 7.7 Hz, pyridine-H), 4.63 (s, 2H, CH_2Cl), 3.38 (d, 2H, J_{PH} = 12.1 Hz, CH_2P), 1.29 (d, 18H, J_{PH} = 12.7 Hz, 6xCH_3), 0.62 (br, 3H, BH_3) ppm. ^{31}P NMR (162 MHz, $CDCl_3$): δ = 47.3 (m) ppm; IR (solid): $\tilde{\nu}$ = 2904 (s), 2849 (w), 2384 (s, B–H), 2347 (w), 1575 (m), 1453 (s), 1069 (m), 993 (w), 971 (w), 823 (s), 744 (s), 681 (w), 625 (m), 600 (m), 522 (w), 432 (s) cm^{-1}, Elemental analysis calcd (%) for [$C_{15}H_{28}BClNP$]: C 60.13, H 9.42, N 4.67; found: C 60.26, H 9.30, N 4.48.

2-(Chloromethyl)-6-((tert-butylphenyl-phosphino)-methyl)pyridine-borane Adduct ***1g***

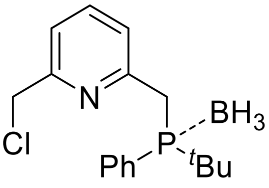

1g

The phosphine-borane adduct **1g** was obtained from 2,6-bis(chloromethyl)pyridine (0.65 g, 3.7 mmol, 1.0 equiv.) and lithium boranyl-*tert*-butylphenyl-phosphanide (1.0 equiv.) as a white solid after flash chromatography. Yield: 0.79 g (67%). ^{1}H NMR (400 MHz, $CDCl_3$): δ = 7.88-7.83 (m, 2H, PPh), 7.57 (t, 1H, J_{HH} = 7.6 Hz, pyridine-H), 7.49-7.40 (m, 5H, pyridine-H, PPh), 7.23 (d, 1H, J_{HH} = 7.6 Hz, pyridine-H), 4.53 (s, 2H, CH_2Cl), 3.78 (t, 1H, J_{PH} = 13.7 Hz, J_{HH} = 13.7 Hz, C*H*HP), 3.64 (dd, 1H, J_{PH} = 9.6 Hz, J_{HH} = 13.7 Hz, CH*H*P), 1.18 (d, 9H, J_{PH} = 14.0 Hz, 3xCH_3), 0.89 (br, 3H, BH_3) ppm. ^{13}C NMR (101 MHz, $CDCl_3$): δ = 155.4 (s, pyridine-C-CH_2Cl), 153.8 (d, J_{PC} = 2.8 Hz, pyridine-C-CH_2P), 137.0 (s, pyridine-CH), 134.2

(d, J_{PC} = 8.2 Hz, 2xphenyl-CH), 131.2 (d, J_{PC} = 2.4 Hz, phenyl-CH), 127.9 (d, J_{PC} = 9.5 Hz, 2xphenyl-CH), 124.8 (d, J_{PC} = 2.5 Hz, pyridine-CH), 120.8 (d, J_{PC} = 2.3 Hz, pyridine-CH), 46.7 (s, CH_2Cl), 30.2 (d, J_{PC} = 2.8 Hz, P-*C*(CH_3)$_3$), 29.9 (d, J_{PC} = 5.0 Hz, CH_2P), 25.6 (d, J_{PC} = 2.1 Hz, P-C(*C*H_3)$_3$) ppm. ^{31}P NMR (162 MHz, $CDCl_3$): δ = 33.3 (m) ppm; IR (solid): $\tilde{\nu}$ = 3054 (w), 2401 (w), 2367 (m, B–H), 1584 (m), 1456 (m), 1433 (m), 1105 (m), 1061 (m), 821 (w), 740 (s), 690 (s), 596 (m), 532 (m), 493 (m), 464 (m), 418 (m) cm^{-1}; ESI-HRMS (m/z, pos): Calculated for $C_{17}H_{24}BClNP$-H: 318.1350 [*M*-H]$^+$; found: 318.1354; Elemental analysis calcd (%) for [$C_{17}H_{24}BClNP$]: C 63.88, H 7.54, N 4.38; found: C 63.95, H 7.25, N 4.29.

*2-(Chloromethyl)-6-((di-adamantylphosphino)-methyl)pyridine-borane Adduct **1h***

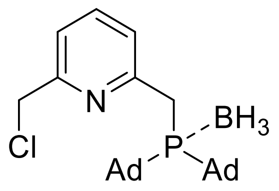

1h

The phosphine-borane adduct **1h** was obtained from 2,6-bis(chloromethyl)pyridine (0.22 g, 1.3 mmol, 1.0 equiv.) and lithium boranyl-di-adamantyl-phosphanide (1.0 equiv.) as a white solid after flash chromatography together with 9% of disubstituted product. Yield: 0.38 g (67%). ^{1}H NMR (400 MHz, $CDCl_3$): δ = 7.62 (t, 1H, J_{HH} = 7.7 Hz, pyridine-H), 7.53 (d, 1H, J_{HH} = 7.9 Hz, pyridine-H) 7.28 (m, 1H, pyridine-H), 4.61 (s, 2H, CH_2Cl), 3.31 (d, 2H, J_{PH} = 12.1 Hz, CH_2P), 2.15-1.70 (m, 30H, adamantyl-H), 0.41 (br, 3H, BH_3) ppm. ^{13}C NMR (101 MHz, $CDCl_3$): δ = 156.2 (d, J_{PC} = 2.0 Hz, pyridine-*C*-CH_2P), 155.2 (s, pyridine-*C*-CH_2Cl), 136.8 (s, pyridine-CH), 125.5 (s, pyridine-CH), 120.7 (s, pyridine-CH), 46.8 (s, CH_2Cl), 38.0 (s, adamantyl-CH_2) 37.6 (d, J_{PC} = 23.8 Hz, adamantyl-C-P), 36.5 (s, adamantyl-CH_2), 28.3 (d, J_{PC} = 7.9 Hz, adamantyl-CH), 26.8 (d, J_{PC} = 23.4 Hz, CH_2P) ppm. ^{31}P NMR (202 MHz, $CDCl_3$): δ = 38.0 (m) ppm; IR (solid): $\tilde{\nu}$ = 2905 (s), 2849 (m), 2368 (w, B–H), 1581 (m), 1451 (m), 1067 (m), 970 (w), 837 (w), 744 (w), 685 (w), 596 (m), 527 (w), 413 (s) cm^{-1}; ESI-HRMS (m/z, pos): Calculated for $C_{27}H_{40}BClNP$-H: 454.2602 [*M*-H]$^+$; found: 454.2610; Elemental analysis calcd (%) for [$C_{27}H_{40}BClNP$]: C 71.14, H 8.84, N 3.07; found: C 71.84, H 9.22, N 3.25.

General Procedure for the Synthesis of Resin-Bound PNP-Pincer Ligands L_1-L_{14}

Step 1

A resin-bound phosphine-borane (**2a**, 1.40 g, 1.57 mmol, 1.0 equiv.), (**2b**, 0.22 g, 0.24 mmol, 1.0 equiv.), (**2c**, 0.25 g, 0.28 mmol, 1.0 equiv.) or (**2d**, 0.12 g, 0.22 mmol, 1.0 equiv.) was swollen in THF (20 mL). After addition of KHMDS (20% in THF, 10 equiv.) under gentle stirring to avoid mechanical abrasion of the resin, the orange resin was allowed to react for 2 hours at room temperature. The supernatant was removed and the resin was washed three times with THF (15 mL) followed by three times with Et_2O (15 mL). Without further purification the BH_3-protected resin-bound potassium phosphides **K·2a-d** were used in the next step.

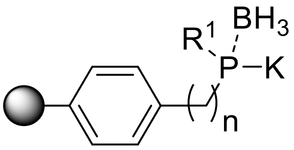

K·2a: MF 1%, n = 1, R^1 = Ph
K·2b: MF 4%, n = 1, R^1 = Ph
K·2c: MF 1%, n = 1, R^1 = Cy
K·2d: PS, n = 0, R^1 = ^{t}Bu

K·1a: Orange resin: ^{31}P NMR (162 MHz, THF): δ = -37.1 (br s) ppm.

K·2b: Yellow resin: ^{31}P NMR (121 MHz, THF:C_6D_6 6:1): δ = -39.5 (br s) ppm.

K·2c: Orange resin: ^{31}P NMR (162 MHz, THF): δ = -34.5 (br s) ppm.

K·2d: Orange resin: ^{31}P NMR (162 MHz, THF): δ = -14.6 (br s) ppm.

Step 2

A previously synthesized BH_3-protected resin-bound potassium phosphide (**K·2a**, 1.57 mmol, 1.0 equiv.), (**K·2b**, 0.24 mmol, 1.0 equiv.), (**K·2c**, 0.28 mmol, 1.0 equiv.) or (**K·2d**, 0.22 mmol, 1.0 equiv.) was swollen in THF (10 mL) and cooled to -78 °C. A 2-(chloromethyl)-6-(phosphinomethyl)pyridine-borane (**1a-h**,1.1 equiv.) was azeotropically dried with toluene (3x5 mL), dissolved in 10 mL THF and added to the resin at -78 °C under gentle stirring to avoid mechanical abrasion. The mixture was left with occasional stirring and allowed to warm up to room temperature overnight. The reaction was monitored using gel-phase ^{31}P NMR and was allowed to react until full conversion was observed. Next, the supernatant was removed and the resin was washed three times with THF (10 mL) followed by three times with Et_2O (10 mL) and dried *in vacuo* yielding a pale yellow resin-bound PNP borane adduct (**3a-n**).

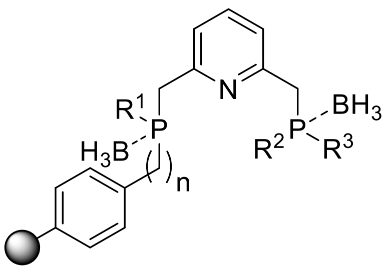

3a: MF 1%, n = 1, R^1 = Ph, R^2 = R^3 = Ph
3b: MF 1%, n = 1, R^1 = Ph, R^2 = R^3 = 4-MeOPh
3c: MF 1%, n = 1, R^1 = Ph, R^2 = R^3 = 4-ClPh
3d: MF 1%, n = 1, R^1 = Ph, R^2 = R^3 = Cy
3e: MF 1%, n = 1, R^1 = Ph, R^2 = R^3 = ^{i}Bu
3f: MF 1%, n = 1, R^1 = Ph, R^2 = R^3 = ^{t}Bu
3g: MF 4%, n = 1, R^1 = Ph, R^2 = R^3 = ^{t}Bu
3h: MF 1%, n = 1, R^1 = Ph, R^2 = Ph, R^3 = ^{t}Bu
3i: MF 1%, n = 1, R^1 = Ph, R^2 = R^3 = Ad
3j: MF 4%, n = 1, R^1 = Ph, R^2 = R^3 = Ad
3k: MF 1%, n = 1, R^1 = Cy, R^2 = R^3 = Ph
3l: MF 1%, n = 1, R^1 = Cy, R^2 = R^3 = ^{t}Bu
3m: PS, n = 0, R^1 = ^{t}Bu, R^2 = R^3 = ^{t}Bu
3n: PS, n = 0, R^1 = ^{t}Bu, R^2 = Ph, R^3 = ^{t}Bu

3a: Pale yellow resin: ^{31}P NMR (162 MHz, THF): δ = 18.4 (br s, -*P*Ph_2-BH_3 and -MF-*P*Ph-BH_3), -11.1 (s, free -*P*Ph_2) ppm.

3b: Yellow resin: ^{31}P NMR (162 MHz, THF): δ = 18.5 (br s, -MF-*P*Ph-BH_3), 14.9 (br, -*P*(4-MeOPh)$_2$-BH_3 and ppm.

3c: Pale yellow resin: ^{31}P NMR (121 MHz, THF:C_6D_6 6:1): δ = 18.0 (br s, -*P*(4-ClPh)$_2$-BH_3 and -MF-*P*Ph-BH_3), -13.5 (s, free -*P*(4-ClPh)$_2$) ppm.

3d: Pale yellow resin: ^{31}P NMR (121 MHz, THF:C_6D_6 6:1): δ = 28.9 (br s, -PCy_2-BH_3), 18.4 (br s, -MF-*P*Ph-BH_3) ppm.

3e: Pale yellow resin: ^{31}P NMR (162 MHz, THF): δ = 21.3 (br s, -MF-*P*Ph-BH_3), 18.4 (s, -*P*^{i}Bu_2-BH_3) ppm.

3f: Pale yellow resin: ^{31}P NMR (162 MHz, THF): δ = 48.0 (s, -*P*^{t}Bu_2-BH_3), 18.8 (br s, -MF-*P*Ph-BH_3) ppm.

3g: Yellow resin: ^{31}P NMR (121 MHz, THF:C_6D_6 6:1): δ = 47.6 (s, -*P*^{t}Bu_2-BH_3), 18.3 (br s, -MF-*P*Ph-BH_3) ppm.

3h: Pale yellow resin: ^{31}P NMR (162 MHz, THF): δ = 33.8 (s, -*P*Ph^{t}Bu-BH_3), 18.6 (br s, -MF-*P*Ph-BH_3) ppm.

3i: Pale yellow resin: ^{31}P NMR (162 MHz, THF): δ = 38.9 (s, -*P*Ad_2-BH_3), 18.8 (br s, -MF-*P*Ph-BH_3) ppm.

3j: Yellow resin: ^{31}P NMR (121 MHz, THF:C_6D_6 2:1): δ = 38.5 (s, -*P*Ad_2-BH_3), 18.2 (br s, -MF-*P*Ph-BH_3) ppm.

3k: Pale orange resin: ^{31}P NMR (162 MHz, THF): δ = 25.8 (br s, -MF-*P*Cy-BH_3), 18.1 (s, -*P*Ph_2-BH_3), -11.1 (s, free -*P*Ph_2) ppm.

3l: Pale orange resin: ^{31}P NMR (162 MHz, THF): δ = 48.2 (s, -*P*^{t}Bu_2-BH_3), 25.9 (br s, -MF-*P*Cy-BH_3) ppm.

3m: Pale yellow resin: ^{31}P NMR (162 MHz, THF): δ = 48.3 (s, -*P*^{t}Bu$_2$-BH$_3$), 32.6 (br s, -PS-*P*^{t}Bu-BH$_3$) ppm.

3n: Pale yellow resin: ^{31}P NMR (162 MHz, THF): δ = 33.5 (br s, -*P*Ph^{t}Bu-BH$_3$), 32.0 (br s, -PS-*P*^{t}Bu-BH$_3$) ppm.

Step 3

A resin-bound PNP borane adduct **3a-n** synthesized in the last step was swollen in 10 mL of diethyl amine and heated to 50 °C overnight with occasional stirring to avoid mechanical abrasion of the resin. The reaction was monitored using gel-phase ^{31}P NMR and was allowed to react until full conversion was observed. Next, the mixture was cooled to room temperature and the supernatant was removed. The resin was washed with three portions of THF (10 mL) followed by three portions of Et$_2$O (10 mL) and dried *in vacuo* yielding a pale yellow resin-bound PNP pincer ligand (**L$_1$**-**L$_{14}$**).

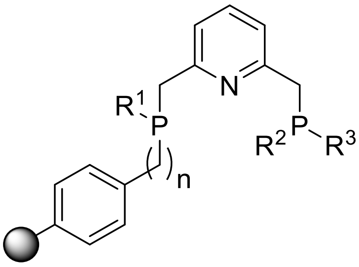

L$_1$: MF 1%, n = 1, R^1 = Ph, R^2 = R^3 = Ph
L$_2$: MF 1%, n = 1, R^1 = Ph, R^2 = R^3 = 4-MeOPh
L$_3$: MF 1%, n = 1, R^1 = Ph, R^2 = R^3 = 4-ClPh
L$_4$: MF 1%, n = 1, R^1 = Ph, R^2 = R^3 = Cy
L$_5$: MF 1%, n = 1, R^1 = Ph, R^2 = R^3 = ^{i}Bu
L$_6$: MF 1%, n = 1, R^1 = Ph, R^2 = R^3 = ^{t}Bu
L$_7$: MF 4%, n = 1, R^1 = Ph, R^2 = R^3 = ^{t}Bu
L$_8$: MF 1%, n = 1, R^1 = Ph, R^2 = Ph, R^3 = ^{t}Bu
L$_9$: MF 1%, n = 1, R^1 = Ph, R^2 = R^3 = Ad
L$_{10}$: MF 4%, n = 1, R^1 = Ph, R^2 = R^3 = Ad
L$_{11}$: MF 1%, n = 1, R^1 = Cy, R^2 = R^3 = Ph
L$_{12}$: MF 1%, n = 1, R^1 = Cy, R^2 = R^3 = ^{t}Bu
L$_{13}$: PS, n = 0, R^1 = ^{t}Bu, R^2 = R^3 = ^{t}Bu
L$_{14}$: PS, n = 0, R^1 = ^{t}Bu, R^2 = Ph, R^3 = ^{t}Bu

L$_1$: Pale yellow resin (288 mg, 0.245 mmol, 99%): ^{31}P NMR (162 MHz, THF): δ = -11.2 (s, -*P*Ph$_2$), -14.0 (br s, -MF-*P*Ph) ppm; IR (KBr): $\tilde{\nu}$ = 3056 (m), 3024 (m), 2918 (m), 2849 (m), 1578 (m), 1487 (m), 1445 (m), 1110 (w), 1071 (w), 838 (w), 743 (m, P-Ar), 697 (s, P-Ar) cm^{-1}; Elemental analysis calcd (%) for **L$_1$** (0.82 mmol·g^{-1}): P 5.08, N 1.15; found: P 4.98, N 0.97.

L$_2$: Pale yellow resin (322 mg, 0.260 mmol, 90%): ^{31}P NMR (162 MHz, THF): δ = -14.0 (br, -*P*(4-MeOPh)$_2$ and -MF-*P*Ph) ppm; IR (KBr): $\tilde{\nu}$ = 3057 (m), 3023 (m), 2919 (m), 2845 (m), 1592 (s), 1497 (s), 1448 (s), 1282 (w), 1246 (m, C-O), 1177 (m), 1102 (w), 824 (w), 750 (m, P-Ar), 698 (s, P-Ar) cm^{-1}; Elemental analysis calcd (%) for **L$_2$** (0.81 mmol·g^{-1}): P 5.01, N 1.13; found: P 5.55, N 0.84.

L_3: Pale yellow resin (322 mg, 0.260 mmol, 90%): ^{31}P NMR (121 MHz, THF:C_6D_6 6:1): δ = -13.3 (br, -*P*(4-ClPh)$_2$), -14.3 (br, -MF-*P*Ph) ppm; IR (KBr): $\tilde{\nu}$ = 3056 (w), 3022 (m), 2917 (m), 2850 (m), 1583 (s), 1495 (m), 1445 (s), 1384 (m), 1081 (w), 1013 (m), 838 (m), 812 (m), 743 (s, P-Ar), 697 (s, P-Ar), 498 (s) cm^{-1}; Elemental analysis calcd (%) for **L_3** (0.77 mmol·g^{-1}): P 4.78, N 1.08, Cl 5.47; found: P 5.41, N 0.75, Cl 5.41.

L_4: Pale yellow resin (195 mg, 0.164 mmol, 97%): ^{31}P NMR (121 MHz, THF:C_6D_6 6:1): δ = 3.0 (s, -*P*Cy$_2$), -14.5 (br s, -MF-*P*Ph) ppm; IR (KBr): $\tilde{\nu}$ = 3056 (w), 3024 (m), 2919 (m), 2855 (m), 1578 (m), 1495 (m), 1446 (s), 1179 (w), 845 (w), 752 (m, P-Ar), 698 (s, P-Ar) cm^{-1}, Elemental analysis calcd (%) for **L_4** (0.84 mmol·g^{-1}): P 5.10, N 1.19; found: P 5.01, N 1.08.

L_5: Pale yellow resin (401 mg, 0.356 mmol, 98%): ^{31}P NMR (162 MHz, THF): δ = -14.7 (br s, -MF-*P*Ph), -33.4 (s, -*P*^{i}Bu$_2$), ppm; IR (KBr): 3057 (w), 3024 (m), 2919 (s), 2868 (m), 1578 (m), 1495 (m), 1448 (s), 1162 (w), 845 (w), 752 (m, P-Ar), 697 (s, P-Ar), 514 (w) cm^{-1}; Elemental analysis calcd (%) for **L_5** (0.84 mmol·g^{-1}): P 5.18, N 1.17; found: P 5.26, N 1.03.

L_6: Pale yellow resin (1.12 g, 0.994 mmol, 95%): ^{31}P NMR (162 MHz, THF): δ = 35.5 (s, -*P*^{t}Bu$_2$), -13.9 (br s, -MF-*P*Ph) ppm; IR (KBr): $\tilde{\nu}$ = 3057 (m), 3024 (m), 2920 (m), 2857 (m), 1578 (m), 1497 (m), 1448 (m), 1179 (w), 1111 (w), 1073 (w), 831 (w), 746 (m, P-Ar), 698 (s, P-Ar), 539 (w) cm^{-1}; Elemental analysis calcd (%) for **L_6** (0.84 mmol·g^{-1}): P 5.18, N 1.17; found: P 4.30, N 0.98.

L_7: Yellow resin (200 mg, 0.231 mmol, 96%): ^{31}P NMR (121 MHz, THF:C_6D_6 6:1): δ = 35.0 (s, -*P*^{t}Bu$_2$), -14.3 (br s, -MF-*P*Ph) ppm; IR (KBr): $\tilde{\nu}$ = 3056 (w), 3024 (w), 2919 (m), 2856 (w), 1577 (m), 1497 (m), 1445 (m), 1111 (w), 1071 (w), 833 (w), 745 (m, P-Ar), 697 (s, P-Ar) cm^{-1}; Elemental analysis calcd (%) for **L_7** (0.87 mmol·g^{-1}): P 5.36; found: P 6.42.

L_8: Pale yellow resin (132 mg, 0.114 mmol, 96%): ^{31}P NMR (162 MHz, THF): δ = 9.0 (s, -*P*Ph^{t}Bu), -14.1 (br s, -MF-*P*Ph) ppm; IR (KBr): $\tilde{\nu}$ = 3056 (w), 3024 (m), 2921 (m), 2853 (m), 1578 (m), 1496 (m), 1448 (m), 1180 (w), 1107 (w), 1072 (w), 837 (w), 746 (m, P-Ar), 697 (s, P-Ar) cm^{-1}; Elemental analysis calcd (%) for **L_8** (0.82 mmol·g^{-1}): P 5.08, N 1.15; found: P 4.51, N 1.08.

L_9: Pale yellow resin (338 mg, 0.262 mmol, 91%): ^{31}P NMR (162 MHz, THF): δ = 32.1 (s, -*P*Ad$_2$), -13.9 (br s, -MF-*P*Ph) ppm; IR (KBr): $\tilde{\nu}$ = 3056 (m), 3024 (m), 2901 (s), 2847 (m), 1578 (m), 1496 (m), 1446 (m), 1111 (w), 835 (w), 746 (m, P-Ar), 697 (s, P-Ar) cm^{-1}; Elemental analysis calcd (%) for **L_9** (0.74 mmol·g^{-1}): P 4.60, N 1.04; found: P 3.57, N 0.86.

L_{10}: Pale yellow resin (320 mg, 0.244 mmol, 95%): ^{31}P NMR (121 MHz, THF:C_6D_6 6:1): δ = 31.5 (s, -*P*Ad$_2$), -14.9 (br s, -MF-*P*Ph) ppm; IR (KBr): $\tilde{\nu}$ = 3057 (w), 3024 (w), 2899

(m), 2848 (w), 1578 (m), 1495 (m), 1445 (m), 1111 (w), 837 (w), 746 (m, P-Ar), 696 (s, P-Ar) cm^{-1}; Elemental analysis found (%) for $\mathbf{L_{10}}$ (0.66 $mmol{\cdot}g^{-1}$): P 8.18.

$\mathbf{L_{11}}$: Pale yellow resin (324 mg, 0.274 mmol, 99%): ^{31}P NMR (162 MHz, THF): δ = -4.8 (br s, -MF-*P*Cy), -10.8 (s, -*P*Ph_2) ppm; IR (KBr): $\tilde{\nu}$ = 3057 (w), 3024 (m), 2920 (m), 2848 (m), 1577 (m), 1490 (m), 1449 (m), 1070 (w), 837 (w), 745 (m, P-Ar), 698 (s, P-Ar) cm^{-1}; Elemental analysis calcd (%) for $\mathbf{L_{11}}$ (0.85 $mmol{\cdot}g^{-1}$): P 5.24, N 1.19; found: P 5.32, N 1.11.

$\mathbf{L_{12}}$: Pale yellow resin (276 mg, 0.242 mmol, 88%): ^{31}P NMR (162 MHz, THF): δ = 35.1 (s, -$P^t$$Bu_2$), -4.7 (br s, -MF-*P*Cy) ppm; IR (KBr): $\tilde{\nu}$ = 3057 (m), 3025 (m), 2922 (s), 2852 (m), 1578 (m), 1497 (m), 1448 (m), 1179 (w), 1072 (w), 822 (w), 753 (m, P-Ar), 698 (s, P-Ar) cm^{-1}; Elemental analysis calcd (%) for $\mathbf{L_{12}}$ (0.88 $mmol{\cdot}g^{-1}$): P 5.42, N 1.23; found: P 5.02, N 1.09.

$\mathbf{L_{13}}$: Pale yellow resin (125 mg, 0.159 mmol, 98%): ^{31}P NMR (162 MHz, THF): δ = 35.2 (s, -$P^t$$Bu_2$), 7.2 (br s, -PS-$P^t$Bu) ppm; IR (KBr): $\tilde{\nu}$ = 3059 (m), 3026 (m), 2928 (s), 2860 (m), 1578 (m), 1495 (m), 1452 (m), 1179 (w), 822 (m), 754 (m, P-Ar), 699 (s, P-Ar) cm^{-1}; Elemental analysis calcd (%) for $\mathbf{L_{13}}$ (1.20 $mmol{\cdot}g^{-1}$): P 7.47, N 1.69; found: P 5.78, N 1.36.

$\mathbf{L_{14}}$: Pale yellow resin (177 mg, 0.220 mmol, 99%): ^{31}P NMR (162 MHz, THF): δ = 8.9 (br s, -*P*Ph^tBu and -PS-P^tBu) ppm; IR (KBr): $\tilde{\nu}$ = 3059 (m), 3025 (m), 2926 (m), 2857 (m), 1579 (m), 1495 (w), 1451 (m), 1180 (w), 825 (w), 749 (m, P-Ar), 698 (s, P-Ar) cm^{-1}; Elemental analysis calcd (%) for $\mathbf{L_{14}}$ (1.18 $mmol{\cdot}g^{-1}$): P 7.29, N 1.65; found: P 5.49, N 1.19.

General Procedure for the Synthesis of Resin-Bound complexes C_1-C_{14}

A previously synthesized resin-bound PNP pincer ligand ($\mathbf{L_1}$-$\mathbf{L_{14}}$, ~80-170 mg, 1.0 equiv.) and [Ru(HCl(PPh_3)$_3$CO] (1.1 equiv.) were weighed into a Schlenk tube. The mixture was suspended in THF (10 mL) and heated to 60 °C under gentle stirring. The reaction mixture was left at 60 °C with occasional stirring to avoid mechanical abrasion of the resin and the progress of the reaction was monitored by gel-phase ^{31}P NMR. Once full complexation of the resin-bound PNP ligand was observed, the mixture was cooled to room temperature and the supernatant was removed. The resin-bound complex was washed with three portions of THF (10 mL), three portions of CH_2Cl_2 (10 mL) followed by three portions of Et_2O (10 mL). After drying *in vacuo* a yellow to brown resin-bound Ru-PNP complex ($\mathbf{C_1}$-$\mathbf{C_{14}}$) was obtained.

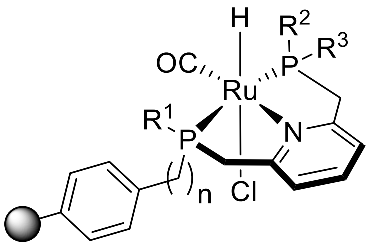

C_1: MF 1%, n = 1, R^1 = Ph, R^2 = R^3 = Ph
C_2: MF 1%, n = 1, R^1 = Ph, R^2 = R^3 = 4-MeOPh
C_3: MF 1%, n = 1, R^1 = Ph, R^2 = R^3 = 4-ClPh
C_4: MF 1%, n = 1, R^1 = Ph, R^2 = R^3 = Cy
C_5: MF 1%, n = 1, R^1 = Ph, R^2 = R^3 = ^{i}Bu
C_6: MF 1%, n = 1, R^1 = Ph, R^2 = R^3 = ^{t}Bu
C_7: MF 4%, n = 1, R^1 = Ph, R^2 = R^3 = ^{t}Bu
C_8: MF 1%, n = 1, R^1 = Ph, R^2 = Ph, R^3 = ^{t}Bu
C_9: MF 1%, n = 1, R^1 = Ph, R^2 = R^3 = Ad
C_{10}: MF 4%, n = 1, R^1 = Ph, R^2 = R^3 = Ad
C_{11}: MF 1%, n = 1, R^1 = Cy, R^2 = R^3 = Ph
C_{12}: MF 1%, n = 1, R^1 = Cy, R^2 = R^3 = ^{t}Bu
C_{13}: PS, n = 0, R^1 = ^{t}Bu, R^2 = R^3 = ^{t}Bu
C_{14}: PS, n = 0, R^1 = ^{t}Bu, R^2 = Ph, R^3 = ^{t}Bu

C_1: Yellow-brown resin (85 mg, 0.063 mmol, 93%): ^{31}P NMR (162 MHz, THF): δ = 51.6 (br s, 2P) ppm; IR (KBr): $\tilde{\nu}$ = 3054 (m), 3024 (m), 2917 (m), 2850 (w), 1926 (s, CO), 1598 (w), 1485 (m), 1443 (m), 1099 (m), 746 (m, P-Ar), 696 (s, P-Ar) cm^{-1}.

C_2: Yellow-orange resin (161 mg, 0.115 mmol, 95%): ^{31}P NMR (162 MHz, THF): δ = 49.7 (br s, 2P) ppm; IR (KBr): $\tilde{\nu}$ = 3055 (w), 3024 (m), 2918 (m), 2846 (w), 1926 (s, CO), 1594 (m), 1497 (s), 1453 (m), 1286 (w), 1250 (m, C-O), 1180 (m), 1102 (m), 750 (m, P-Ar), 697 (s, P-Ar) cm^{-1}.

C_3: Yellow-orange resin (85 mg, 0.063 mmol, 93%): ^{31}P NMR (121 MHz, THF:C_6D_6 6:1): δ = 50.8 (br s, 2P) ppm; IR (solid): $\tilde{\nu}$ = 3055 (w), 3023 (m), 2917 (m), 2850 (w), 1927 (s, CO), 1599 (m), 1570 (w), 1485 (m), 1450 (s), 1384 (m), 1183 (w), 1081 (m), 1011 (m), 965 (w), 839 (m), 815 (m), 741 (s, P-Ar), 694 (s, P-Ar), 494 (s) cm^{-1}.

C_4: Yellow-brown resin (123 mg, 0.094 mmol, 84%): ^{31}P NMR (121 MHz, THF:C_6D_6 6:1): δ = 66.8 (br s, -*P*Cy$_2$), 50.0 (br s, -MF-*P*Ph) ppm; IR (KBr): $\tilde{\nu}$ = 3056 (w), 3024 (m), 2918 (m), 2856 (w), 1920 (s, CO), 1596 (w), 1487 (w), 1450 (m), 752 (m, P-Ar), 698 (s, P-Ar) cm^{-1}.

C_5: Yellow-brown resin (171 mg, 0.131 mmol, 91%): ^{31}P NMR (162 MHz, THF): δ = 59.6 (br s, 1P), 46.7 (br s, 1P) ppm; IR (KBr): $\tilde{\nu}$ = 3024 (w), 2918 (m), 2863 (w), 1921 (s, CO), 1599 (w), 1494 (w), 1453 (m), 842 (w), 751 (m, P-Ar), 698 (s, P-Ar) cm^{-1}.

C_6: Yellow-brown resin (175 mg, 0.134 mmol, 93%): ^{31}P NMR (162 MHz, THF): δ = 91.2 (br s, -*P*^{t}Bu$_2$), 56.5 (br s, -MF-*P*Ph) ppm; IR (KBr): $\tilde{\nu}$ = 3024 (w), 2918 (m), 2866 (w), 1921 (s, CO), 1599 (w), 1496 (w), 1454 (m), 842 (w), 750 (m, P-Ar), 698 (s, P-Ar) cm^{-1}.

C_7: Yellow-brown resin (218 mg, 0.165 mmol, 71%): ^{31}P NMR (162 MHz, THF): δ = 91.3 (br s, -*P*^{t}Bu$_2$), 56.4 (br s, -MF-*P*Ph) ppm; IR (solid): $\tilde{\nu}$ = 3024 (w), 2917 (m), 2852 (w),

1918 (s, CO), 1599 (m), 1492 (w), 1451 (m), 835 (w), 744 (m, P-Ar), 695 (s, P-Ar) cm^{-1}.

C_8: Orange-brown resin (88 mg, 0.076 mmol, 88%): ^{31}P NMR (162 MHz, THF): $\delta = 76.3$ (br s, -*P*Ph^{t}Bu), 65.2 (br s, -*P*Ph^{t}Bu), 53.5 (br s, -MF-*P*Ph) ppm; IR (KBr): $\tilde{\nu} = 3055$ (w), 3024 (w), 2918 (m), 2855 (m), 1921 (s, CO), 1600 (w), 1497 (w), 1450 (m), 1105 (w), 750 (m, P-Ar), 697 (s, P-Ar) cm^{-1}.

C_9: Yellow resin (203 mg, 0.139 mmol, 92%): ^{31}P NMR (162 MHz, THF): $\delta = 83.7$ (br s, -*P*Ad_2), 56.5 (br s, -MF-*P*Ph), 31.0 (-*P*Ad_2 of free ligand) ppm; IR (KBr): $\tilde{\nu} = 3056$ (w), 3024 (m), 2903 (s), 2849 (m), 1920 (s, CO), 1597 (s), 1490 (w), 1449 (m), 1109 (w), 967 (w), 748 (m, P-Ar), 697 (s, P-Ar) cm^{-1}.

C_{10}: Yellow-brown resin (325 mg, 0.220 mmol, 90%): ^{31}P NMR (162 MHz, THF): $\delta = 86.9$ (br s, -*P*Ad_2), 58.8 (br s, -MF-*P*Ph) ppm; IR (solid): $\tilde{\nu} = 3055$ (w), 3024 (m), 2902 (m), 1918 (s, CO), 1595 (m), 1487 (w), 1448 (m), 1105 (w) 746 (m, P-Ar), 695 (s, P-Ar) cm^{-1}.

C_{11}: Yellow-brown resin (111 mg, 0.086 mmol, 93%): ^{31}P NMR (162 MHz, THF): $\delta = 61.0$ (br s, -*P*Cy), 51.5 (br s, -MF-*P*Ph_2) ppm; IR (KBr): $\tilde{\nu} = 3055$ (w), 3024 (w), 2920 (m), 2850 (m), 1924 (s, CO), 1598 (w), 1494 (m), 1450 (m), 1105 (m), 750 (m, P-Ar), 697 (m, P-Ar) cm^{-1}.

C_{12}: Yellow-brown resin (129 mg, 0.099 mmol, 90%): ^{31}P NMR (162 MHz, THF): $\delta = 90.7$ (br s, -*P* ^{t}Bu_2), 66.8 (br s, -MF-*P*Cy) ppm; IR (KBr): $\tilde{\nu} = 3024$ (w), 2921 (m), 2852 (m), 1919 (s, CO), 1598 (w), 1497 (w), 1451 (m), 1179 (w), 752 (m, P-Ar), 698 (m, P-Ar) cm^{-1}.

C_{13}: Orange resin (113 mg, 0.119 mmol, 94%): ^{31}P NMR (162 MHz, THF): $\delta = 90.7$ (br s, -*P* ^{t}Bu_2), 79.8 (br s, -*P*Ph^{t}Bu), 64.9 (br s, -*P*Ph^{t}Bu) ppm; IR (KBr): $\tilde{\nu} = 3024$ (w), 2926 (m), 2864 (m), 1916 (s, CO), 1598 (w), 1458 (m), 1178 (w), 1107 (w), 836 (w), 752 (m, P-Ar), 698 (m, P-Ar) cm^{-1}.

C_{14}: Orange resin (110 mg, 0.107 mmol, 94%): ^{1}H MAS NMR (400 MHz): $\delta = -13.85$ (s, Ru–H) ppm; ^{13}C CP-MAS NMR (101 MHz): $\delta = 211.0$ (CO), 162.1 (pyridine-*C*), 145.5 (pyridine-*C*), 128.1 (PS-*C*, P-Ph), 120.8 (pyridine-*C*), 40.5 (PS-*C*, *C*H_2P), 35.0 (P-*C*$(CH_3)_3$), 31.9 (P-*C*$(CH_3)_3$), 27.5 (P-C(*C*$H_3)_3$) ppm; ^{31}P MAS NMR (400 MHz): $\delta = 78.9$ (br s), 65.1 (br s) ppm; IR (KBr): $\tilde{\nu} = 3056$ (w), 3025 (w), 2926 (m), 2861 (m), 1917 (s, CO) 1597 (w), 1456 (m), 1106 (w), 750 (m, P-Ar), 698 (m, P-Ar) cm^{-1}.

Synthesis of PNP-Borane Adduct 4

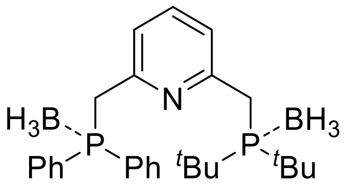

4

n-BuLi (0.25 mL, 0.62 mmol, 2.5 M in hexanes, 1.2 equiv.) was added dropwise to a solution of di-*tert*-butylphosphino-borane adduct (100 mg, 0.62 mmol, 1.2 equiv.) in dry THF at -78 °C,. The solution was stirred for 30 min at -78 °C and subsequently warmed to room temperature and was left for an additional amount of time until full conversion was achieved according to ^{31}P NMR. **1a** (156 mg, 0.52 mmol, 1.0 equiv.) was dissolved in dry THF and cooled to -78 °C. Next, the freshly prepared lithium boranyl phosphanide solution in THF was added. The mixture was warmed to room temperature overnight leading to a yellow solution. The solvent was removed under vacuum and the yellow residue was dissolved in CH_2Cl_2. The organic phase was washed with water and brine and subsequently dried over $MgSO_4$. After filtration, the solvent was removed under reduced pressure. The residue was purified *via* flash chromatography (1:4 Hexanes : CH_2Cl_2) yielding **4** as a white solid (220 mg, 91% yield). ^{1}H NMR (300 MHz, $CDCl_3$): δ = 7.75-7.68 (m, 4H, PPh), 7.50-7.38 (m, 8H, PPh and pyridine-H), 6.96 (d, 1H, J_{HH} = 6.7 Hz, pyridine-H), 3.78 (d, 2H, J_{PH} = 12.0 Hz, CH_2PPh), 3.11 (d, 2H, J_{PH} = 12.2 Hz, CH_2P^{t}Bu), 1.18 (d, 18H, J_{HH} = 12.7 Hz, $C(CH_3)_3$) , 0.65 (br, 6H, BH_3) ppm. ^{13}C NMR (101 MHz, $CDCl_3$): δ = 155.1 (s, pyridine-*C*-CH_2P), 152.2 (d, J_{PC} = 3.2 Hz, pyridine-*C*-CH_2P), 136.2 (s, pyridine-CH), 132.8 (d, J_{PC} = 9.3 Hz, 4xAr-CH), 131.3 (s, 2xAr-CH), 129.2 (d, J_{PC} = 55.0 Hz, 2xAr-C-P), 128.7 (d, J_{PC} = 10.0 Hz, 4xAr-CH), 123.9 (s, pyridine-CH), 123.0 (s, pyridine-CH), 36.2 (d, J_{PC} = 32.3 Hz, CH_2PPh), 32.8 (d, J_{PC} = 25.3 Hz, 2xP-*C*$(CH_3)_3$), 28.9 (d, J_{PC} = 23.3 Hz, CH_2P^{t}Bu), 28.2 (s, 2xP-C($C$$H_3)_3$) ppm. ^{31}P NMR (121 MHz, $CDCl_3$): δ = 47.0-46.4 (m, P^{t}Bu$_2$), 18.4-17.9 (m, PPh_2) ppm; IR (solid): $\tilde{\nu}$ = 2965 (w), 2902 (w), 2403 (m, BH_3), 2373 (m, BH_3), 2348 (m, BH_3), 1588 (w), 1571 (m, C=N), 1478 (w), 1451 (s), 1434 (s), 1391 (w), 1367 (m), 1276 (w), 1231 (w), 1185 (w), 1158 (w), 1132 (w), 1104 (m), 1059 (s), 1024 (m), 948 (w), 837 (w), 810 (m), 740 (s), 723 (m), 690 (s), 627 (m), 577 (m), 490 (m), 469 (m), 436 (m) cm^{-1}; ESI-HRMS (m/z, pos): Calculated for $C_{27}H_{41}B_2NP_2+H^+$: 464.2973 [M+H]$^+$; found: 464.2998; Elemental analysis calcd (%) for [$C_{27}H_{41}B_2NP_2$]: C 70.01, H 8.92, N 3.02; found: C 70.34, H 9.13, N 3.46.

Synthesis of homogeneous complex 5

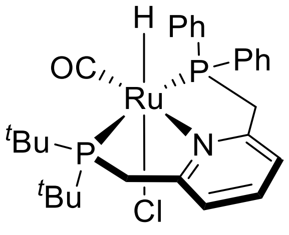

5

Step1

The phosphine-borane **4** (188 mg, 0.41 mmol, 1.0 equiv.) was dissolved in 10 mL of diethyl amine and stirred overnight at 50 °C. After cooling to room temperature the excess diethyl amine was removed. The remaining oil was dried *in vacuo* at 40 °C overnight and analyzed by ^{31}P NMR (162 MHz, C_6D_6): δ = 34.9 (s, -P^{t}Bu$_2$), -11.3 (s, -PPh$_2$) ppm. The ligand was used in the next step without further purification.

Step 2

[Ru(HCl(PPh$_3$)$_3$CO] (387 mg, 0.41 mmol, 1.0 equiv.) and 15 ml of THF were added to the colorless oil of the PNP ligand prepared in the previous step. The suspension was heated to 60 °C and the resulting yellow solution was stirred for 5 hours. All volatiles were removed *in vacuo*. Next the yellow residue was dissolved in 1 mL of THF and 10 mL of *n*-pentane were added to precipitate the pale yellow solid. After filtration, the solid was washed with *n*-pentane (3x5 mL) and dried *in vacuo* yielding Ru-complex **5** (202 mg, 83%). Crystals suitable for X-ray diffraction could be obtained through vapor diffusion of *n*-pentane into a solution of **5** in CH_2Cl_2 over two days at room temperature. ^{1}H NMR (400 MHz, CD_2Cl_2): δ = 7.88-7.83 (m, 2H, phenyl-CH), 7.60-7.58 m, 3H, 2xphenyl-CH, 1xpyridine-H), 7.46-7.45 (m, 3H, phenyl-CH), 7.35-7.27 (m, 5H, 3xphenyl-CH, 2xpyridine-H), 4.89 (dd, 1H, J_{PH} = 9.5 Hz, J_{HH} = 16.0 Hz, C*H*HPPh$_2$), 4.12 (ddd, 1H, J_{HH} = 2.6 Hz, J_{PH} = 12.1 Hz, J_{HH} = 16.0 Hz, CH*H*PPh$_2$), 3.73-3.66 (m, 1H, C*H*HP^{t}Bu$_2$), 3.37 (dd, 1H, J_{HH} = 8.3 Hz, J_{PH} = 16.6 Hz, CH*H*P^{t}Bu$_2$), 1.42 (d, 9H, J_{PH} = 9.0 Hz, PC(CH$_3$)$_3$), 1.39 (d, 9H, J_{PH} = 9.0 Hz, PC(CH$_3$)$_3$), -14.51 (dd, 1H, J_{PH} = 17.1 Hz, J_{PH} = 20.5 Hz, Ru–H) ppm. ^{1}H-NMR (300 MHz, C_6D_6): δ = 8.26-8.19 (m, 2H, phenyl-CH), 7.64-7.54 m, 2H, phenyl-CH), 7.13-7.04 (m, 3H, phenyl-CH), 6.94-6.86 (m, 3H, phenyl-CH), 6.75 (t, 1H, J_{HH} = 7.7 Hz, pyridine-H), 6.52 (d, 1H, J_{HH} = 7.7 Hz, pyridine-H) 6.37 (d, 1H, J_{HH} = 7.7 Hz, pyridine-H), 5.15 (dd, 1H, J_{PH} = 9.5 Hz, J_{HH} = 15.6 Hz, C*H*HPPh$_2$), 3.65 (ddd, 1H, J_{HH} = 2.9 Hz, J_{PH} = 12.2 Hz J_{HH} = 15.6 Hz, CH*H*PPh$_2$), 3.11-3.02 (m, 1H, C*H*HP^{t}Bu$_2$), 2.70 (dd, 1H, J_{HH} = 7.8 Hz, J_{PH} = 16.5 Hz, CH*H*P^{t}Bu$_2$), 1.45 (d, 9H, J_{PH} = 13.2 Hz, PC(CH$_3$)$_3$), 1.21 (d, 9H, J_{PH} = 12.8 Hz, PC(CH$_3$)$_3$), -13.87 (dd, 1H,

J_{HP} = 17.5 Hz, J_{PH} = 20.4 Hz, Ru–H) ppm. ^{13}C NMR (101 MHz, CD_2Cl_2): δ = 208.5 (t, J_{PC} = 12.2 Hz, Ru-CO), 163.6 (s, pyridine-*C*-CH_2P), 160.9 (dd, J_{PC} = 3.0 Hz, 7.8 Hz, pyridine-*C*-CH_2P), 138.5 (dd, J_{PC} = 1.6 Hz, 41.5 Hz Ar-C-P), 137.4 (s, pyridine-CH), 133.4 (dd, J_{PC} = 1.4 Hz, 11.5 Hz, 2xAr-CH), 133.4 (dd, J_{PC} = 2.6 Hz, 39.6 Hz, Ar-C-P), 132.3 (d, J_{PC} = 12.1 Hz, 2xAr-CH), 130.5 (d, J_{PC} = 2.2 Hz, Ar-CH), 130.1 (d, J_{PC} = 2.0 Hz, Ar-CH), 128.7 (d, J_{PC} = 9.8 Hz, 4xAr-CH), 120.9 (d, J_{PC} = 10.3 Hz, pyridine-CH), 120.2 (d, J_{PC} = 9.3 Hz, pyridine-CH), 44.3 (d, J_{PC} = 24.2 Hz, CH_2PPh), 38.4 (d, J_{PC} = 16.3 Hz, CH_2P^tBu), 37.8 (dd, J_{PC} = 5.4 Hz, 8.2 Hz, P-*C*$(CH_3)_3$), 34.6 (d, J_{PC} = 24.2 Hz, P-*C*$(CH_3)_3$), 30.1 (d, J_{PC} = 4.0 Hz, 2xP-C(CH_3)$_3$), 29.3 (d, J_{PC} = 4.6 Hz, 2xP-C(CH_3)$_3$) ppm. ^{31}P NMR (162 MHz, CD_2Cl_2): δ = 90.4 (d, J_{PP} = 266.6 Hz, P^tBu_2) 53.6 (d, J_{PP} = 266.6 Hz, PPh_2) ppm; IR (solid): $\tilde{\nu}$ = 2953 (w), 2895 (w), 2863 (w), 2034 (w, Ru–H), 1887(s, CO), 1597 (w), 1584 (w), 1458 (m), 1433 (m), 1386 (w), 1366 (w), 1281 (w), 1179 (w), 1187 (m), 1018 (w), 962 (w), 837 (m), 814 (m), 780 (w), 751 (m), 713 (w), 693 (s), 623 (m), 511 (m), 474 (m), 457 (m) cm^{-1}, Elemental analysis calcd (%) for [$C_{28}H_{36}ClNOP_2Ru$]: C 55.95, H 6.04, N 2.33; found: C 54.56, H 6.28, N 2.93.

General Procedure for Ru-catalyzed Ester Hydrogenation

The hydrogenation experiments were performed in a stainless steel autoclave charged with an insert suitable for up to 12 reaction vessels (2 mL) including Teflon mini stirring bars. Inside a glove box, a reaction vessel was charged with a resin-bound Ru-PNP complex **C_1-C_{14}** (~7 mg, 5.0 µmol, 1.0 mol%). To the reaction vessel 0.5 mL of a stock solution of KO^tBu (10 mol%) in THF was added and the mixture was stirred for 5 minutes. Next, 0.5 mL of the substrates **S_1-S_{12}** (0.5 mmol) and the internal standard dodecane (50 mol%) dissolved in THF were added. Subsequently, the autoclave was purged three times with 10 bar of argon gas and the insert loaded with reaction vessels was transferred into the autoclave. Next, the autoclave was purged three times with 10 bar of H_2 and then pressurized (30-50 bar) and heated to the desired temperature. The reaction mixtures were gently stirred at 450 rpm for 16-24 hours. The autoclave was cooled to room temperature, depressurized and the conversion was determined by GC-FID measurements using the following column and conditions:

Restek RTX-1 column (30 m, 0.25 mm, 0.1 µm): T_0 = 50 °C, ΔT = 8 °C min^{-1} to 180 °C, then hold for 2 min.

S_1: t_r (ester) = 4.66 min, t_r (alcohol) = 5.77 min, t_r (transester) = 20.58 min.
S_2: t_r (ester) = 4.75 min, t_r (alcohol) = 5.67 min, t_r (transester) = 13.83 min.

S_3: t_r (ester) = 13.78 min, t_r (alcohol) = 5.73 min.

S_4: t_r (ester) = 2.49 min, t_r (alcohol) = 3.03 min, t_r (transester) = 13.02 min

S_5: t_r (ester) = 2.47 min, t_r (alcohol) = 2.83 min, t_r (transester) = 7.87 min.

S_6: t_r (ester) = 13.02 min, t_r (alcohol) = 2.49 min.

S_7: t_r (ester) = 1.81 min, t_r (alcohol) = 1.64 min, t_r (transester) = 6.45 min.

S_8: t_r (ester) = 3.15 min, t_r (alcohol) = 4.62 min, t_r (transester) = 12.46 min.

S_9: t_r (ester) = 5.13 min.

Agilent HP-5 column (30 m, 0.25 mm, 0.1 µm): T_0 = 80 °C, hold for 2 min then ΔT = 10 °C min^{-1} to 160 °C, then ΔT = 15 °C min^{-1} to 240 °C, then ΔT = 15 °C min^{-1} to 300 °C, then hold for 5 min.

S_{10}: t_r (diester) = 21.08 min, t_r (diol) = 19.91 min, t_r (monoester) = 20.52 min.

S_{11}: t_r (lactone) = 5.89 min, t_r (diol) = 6.28 min.

S_{12}: t_r (lactone) = 6.54 min, t_r (diol) = 6.81 min.

Table SI 1 Further conditions for hydrogenation of **S_1** using **C_2**, **C_6** and **C_9**.[a]

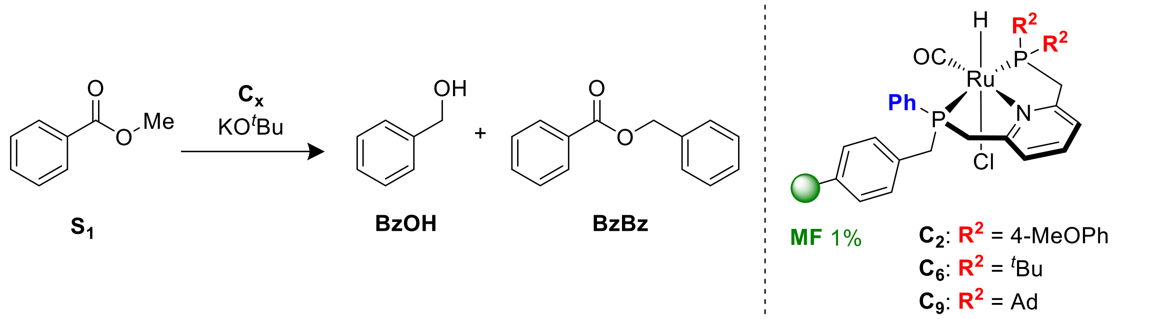

Entry	Complex	Cat. loading [mol%]	Base [mol%]	Temp. [°C]	Pressure [bar]	Time [h]	Conversion [%][b]	Selectivity [%][c]
1	**C_6**	1	5	80	50	16	76	88
2	**C_6**	1	10	80	30	16	97	99
3	**C_6**	1	10	80	20	16	87	95
4	**C_2**	1	10	60	50	16	43	75
5	**C_6**	1	10	60	50	16	77	92
6	**C_9**	1	10	60	50	16	91	99
7	**C_6**	1	10	40	50	16	9	98
8	**C_6**	1	10	60	50	24	95	98
9	**C_6**	0.5	10	60	50	24	88	96
10	**C_6**	0	10	80	50	16	0	0

[a] General conditions: substrate (0.5 mmol), THF (1 mL). [b] Conversion of **S_1** determined by GC using dodecane as internal standard. [c] Selectivity towards BzOH.

General Procedure for Batch Recycling Experiments

The first ester hydrogenation cycle was performed as described above using **C_6** (5 μmol, 1.0 mol%), 1.0 mL of a stock solution of **S_1** (0.5 M), KO^{t}Bu (10 mol%) and the internal standard dodecane (50 mol%) in THF at 100 °C and 50 bar H_2. After 2 hours the autoclave was cooled and depressurized and the reaction vessel was removed. Keeping the catalyst under an argon atmosphere the supernatant was removed. Next, new stock solution of **S_1** (0.5 M, 1.0 mL) was added to the reaction vessel and the autoclave was then charged with H_2 and a new reaction cycle was started. The supernatant was submitted for GC-FID analysis.

ICP-OES Sample Preparation: After each recycling experiment the supernatant was treated with 1 mL of aqua regia and heated to 80 °C for 1 hour. Next the solution was diluted with water to 10 mL and submitted for ICP-OES analysis.

NMR Spectra

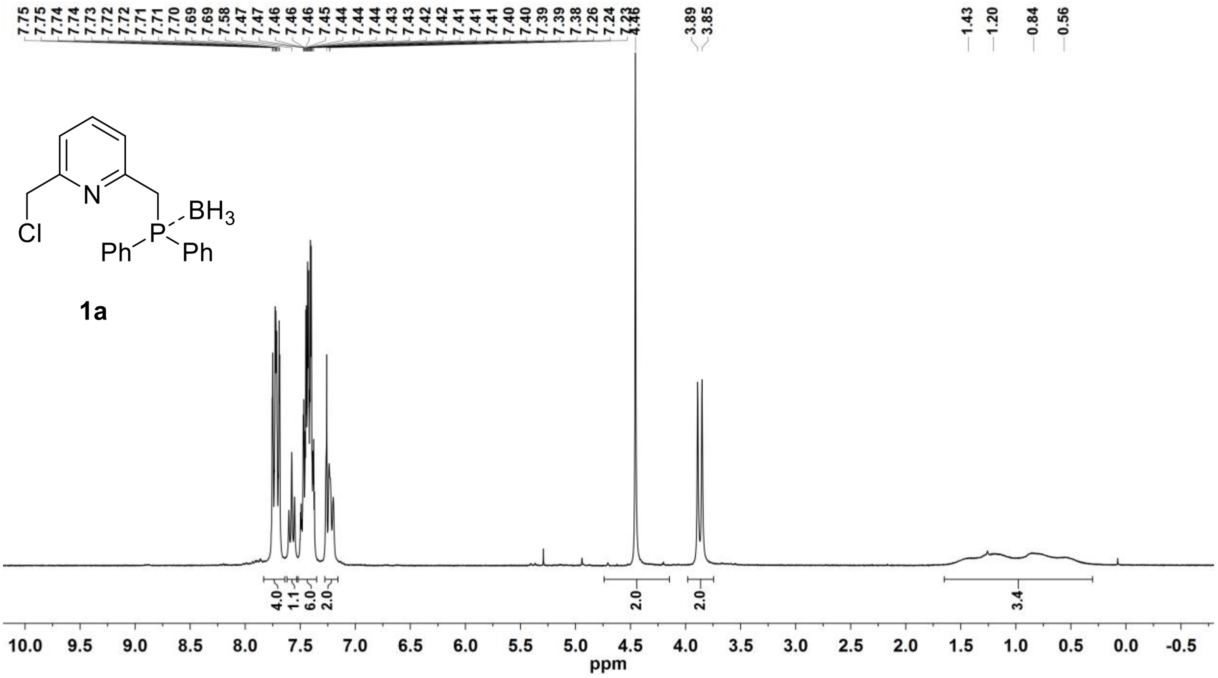

Figure SI 1 ^{1}H NMR spectrum of **1a** (300 MHz, $CDCl_3$).

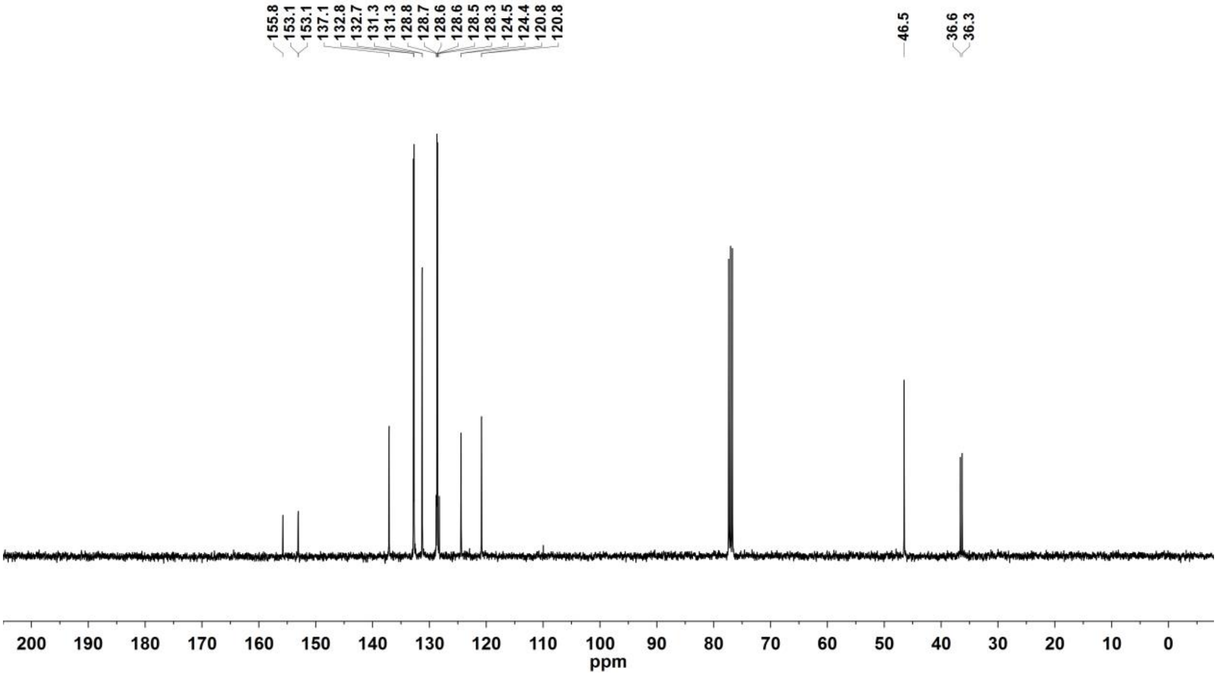

Figure SI 2 ^{13}C NMR spectrum of **1a** (101 MHz, $CDCl_3$).

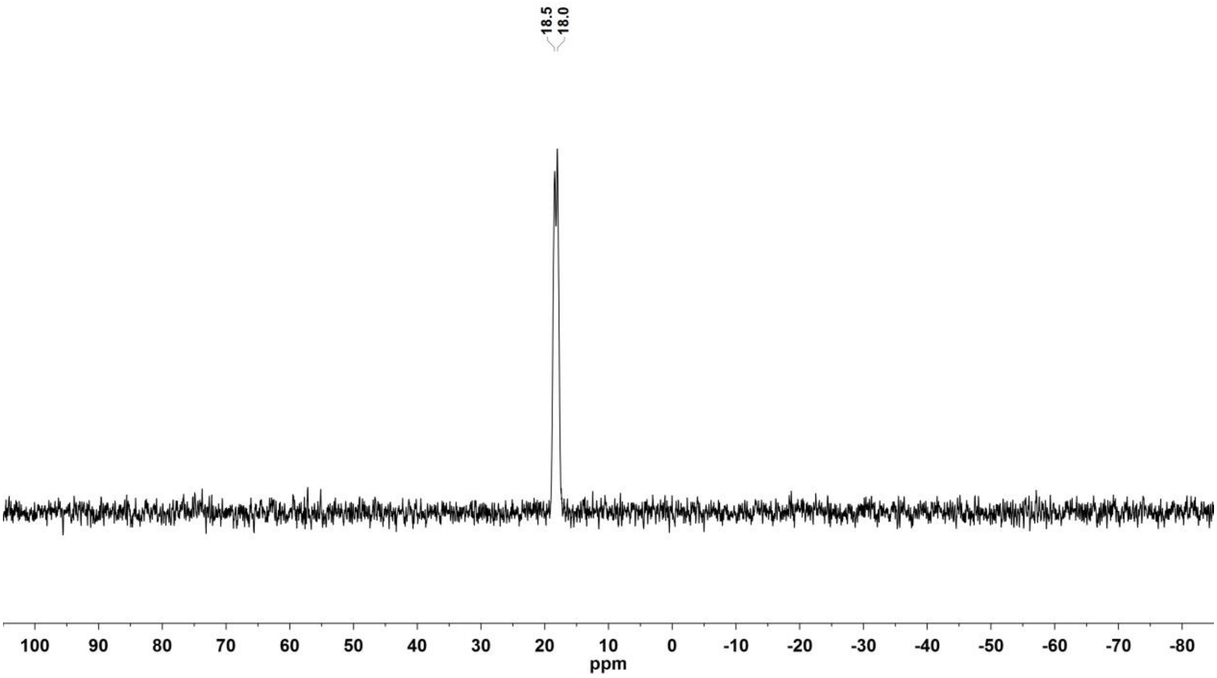

Figure SI 3 ^{31}P NMR spectrum of **1a** (162 MHz, $CDCl_3$).

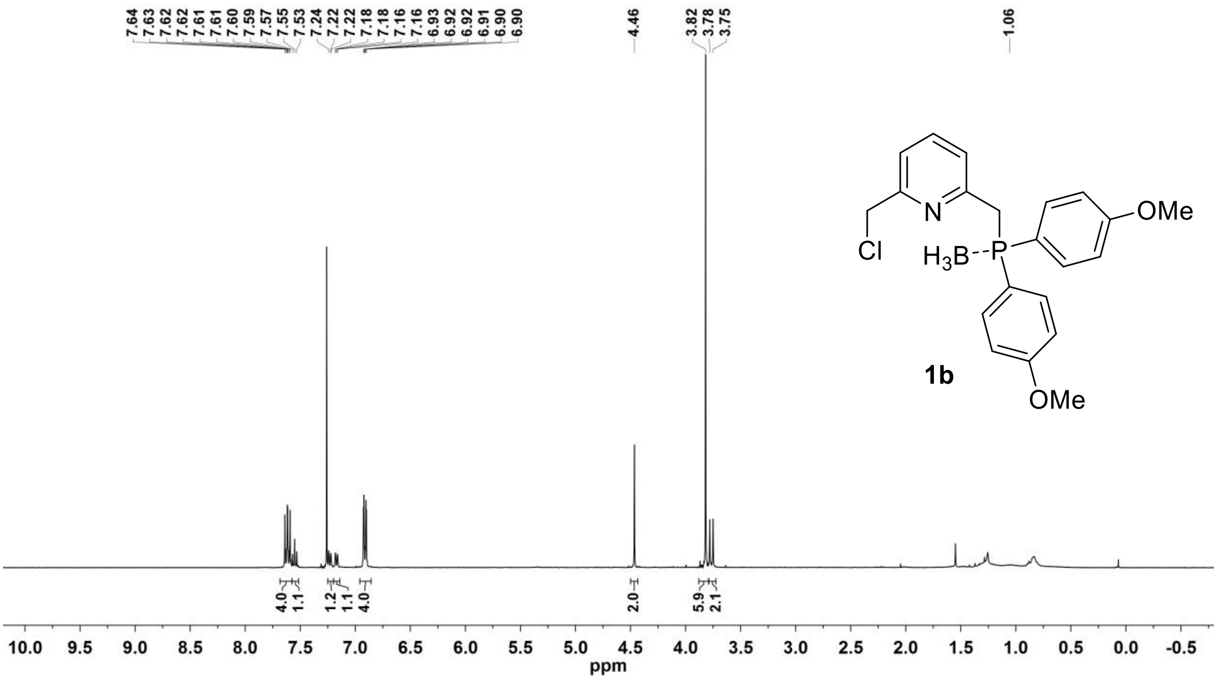

Figure SI 4 ^{1}H NMR spectrum of **1b** (400 MHz, $CDCl_3$).

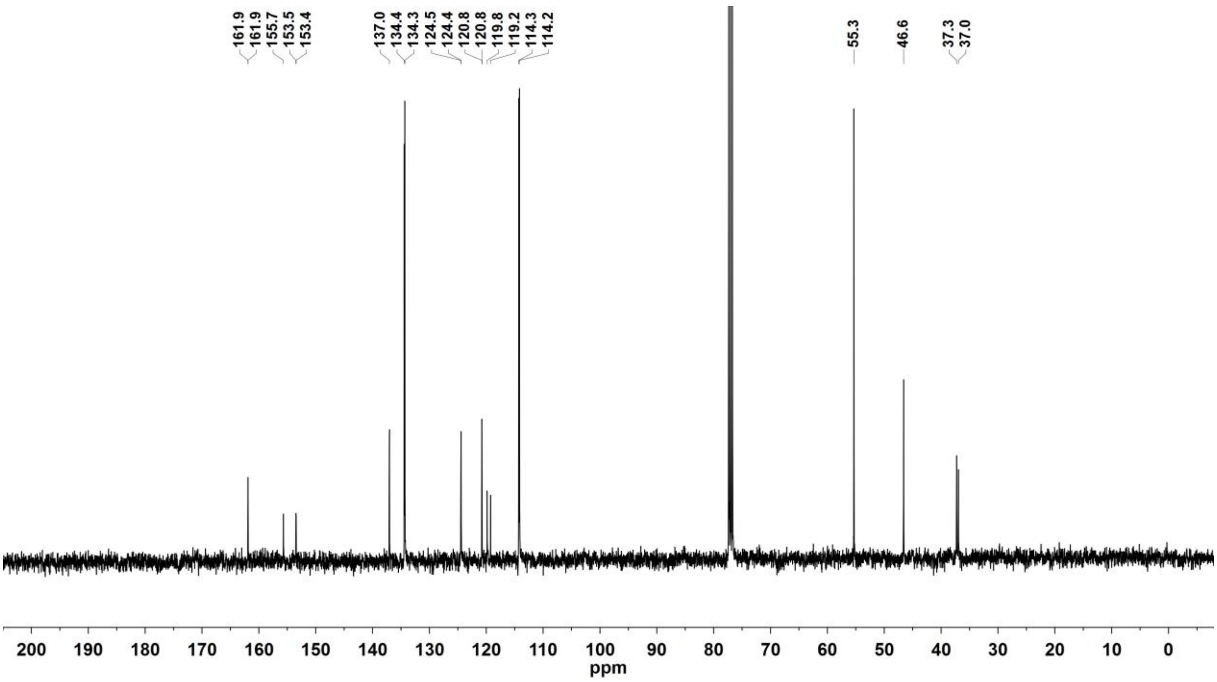

Figure SI 5 ^{13}C NMR spectrum of **1b** (101 MHz, $CDCl_3$).

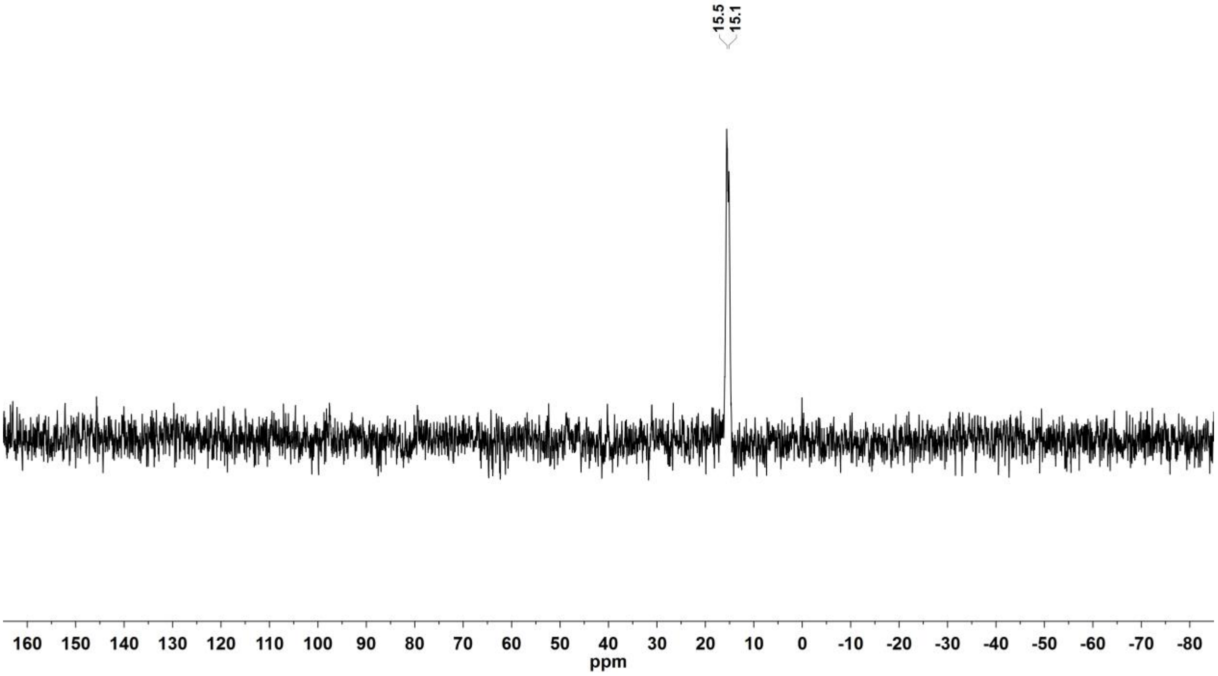

Figure SI 6 ^{31}P NMR spectrum of **1b** (162 MHz, $CDCl_3$).

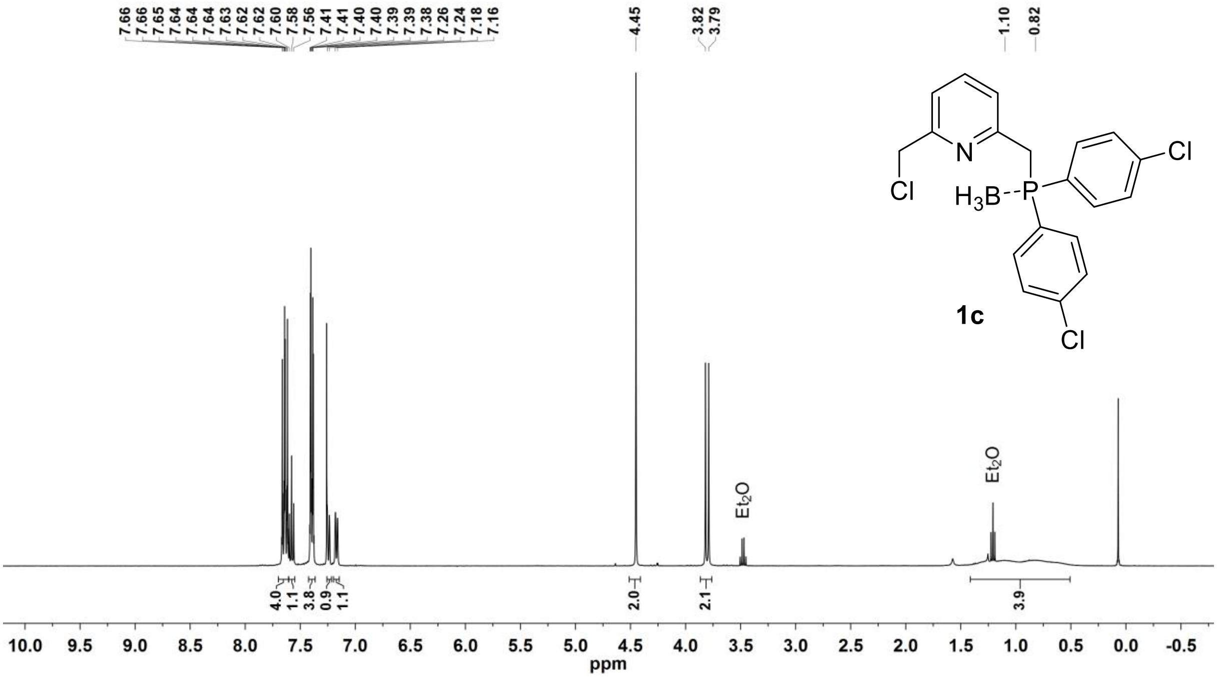

Figure SI 7 ^{1}H NMR spectrum of **1c** (300 MHz, $CDCl_3$).

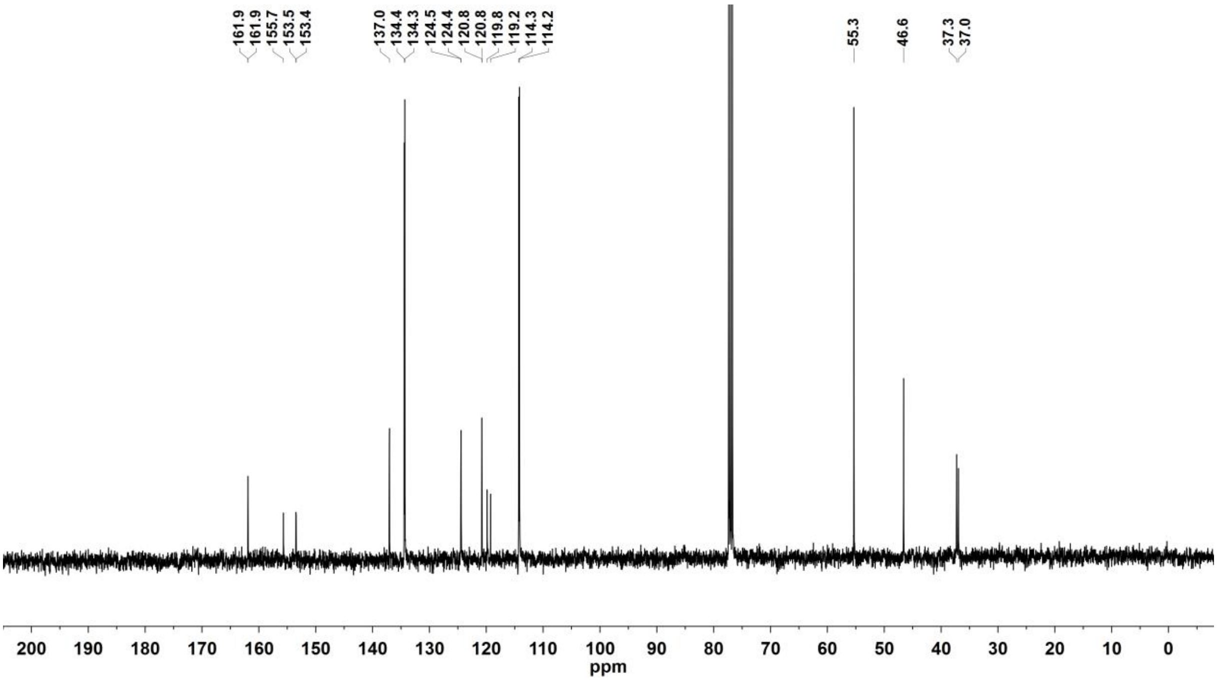

Figure SI 8 ^{13}C NMR spectrum of **1c** (75 MHz, $CDCl_3$).

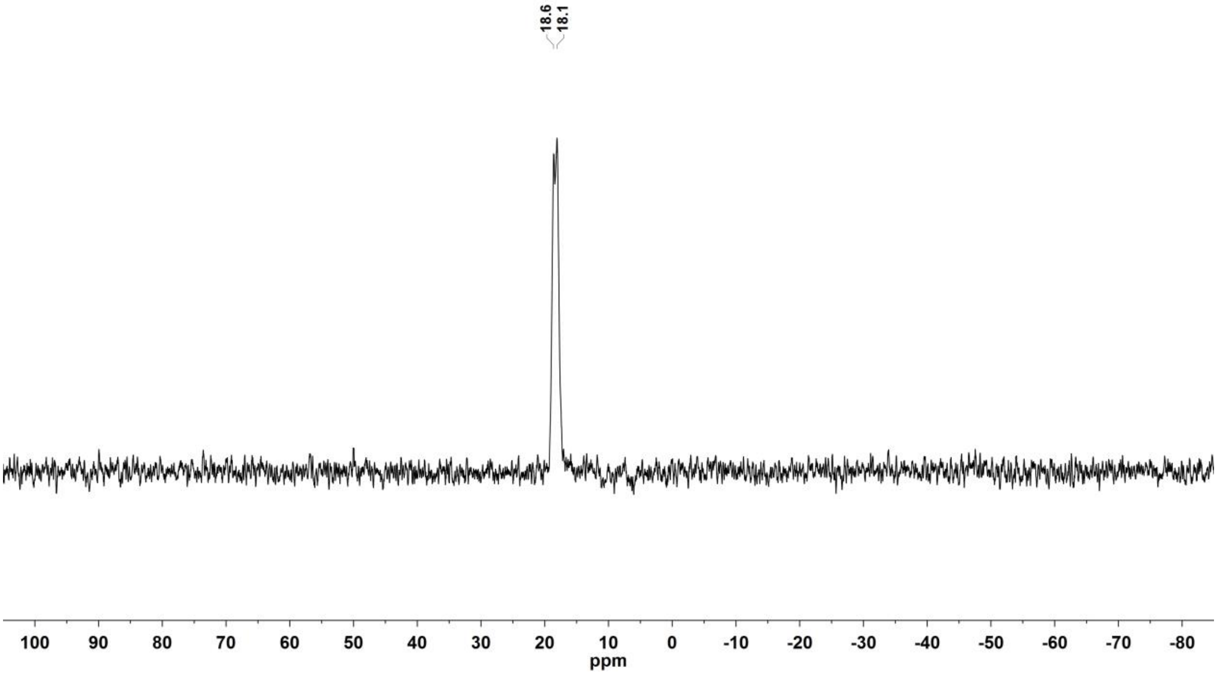

Figure SI 9 ^{31}P NMR spectrum of **1c** (121 MHz, $CDCl_3$).

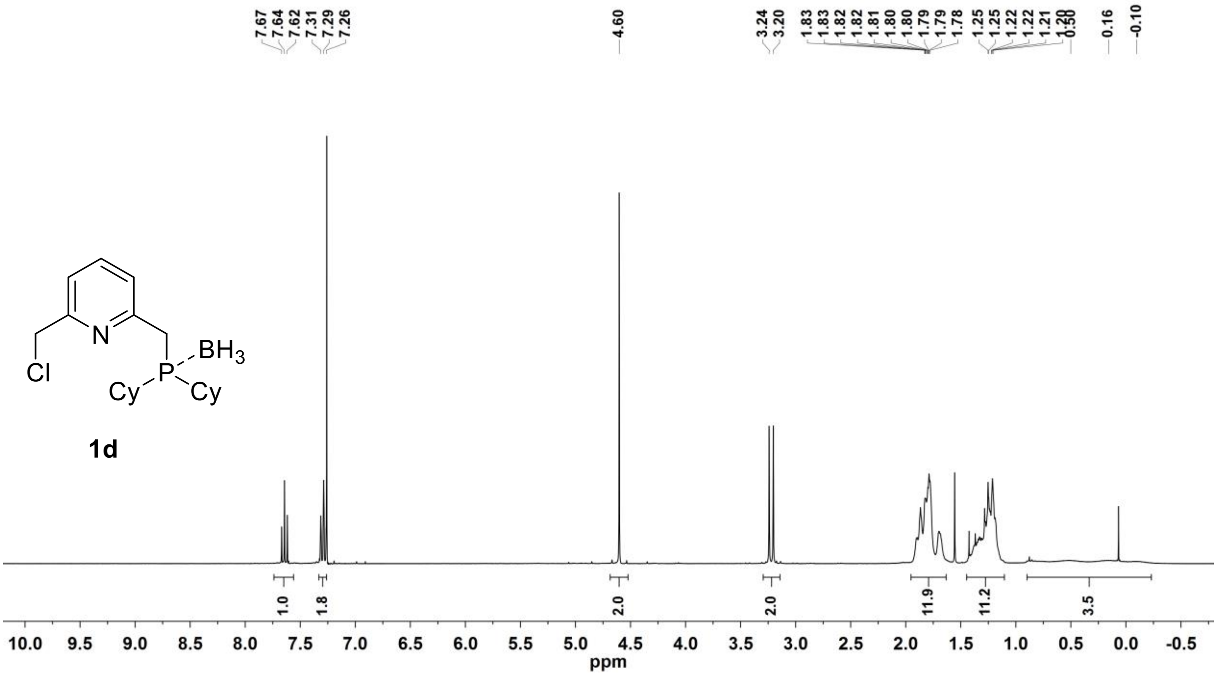

Figure SI 10 ^{1}H NMR spectrum of **1d** (300 MHz, $CDCl_3$).

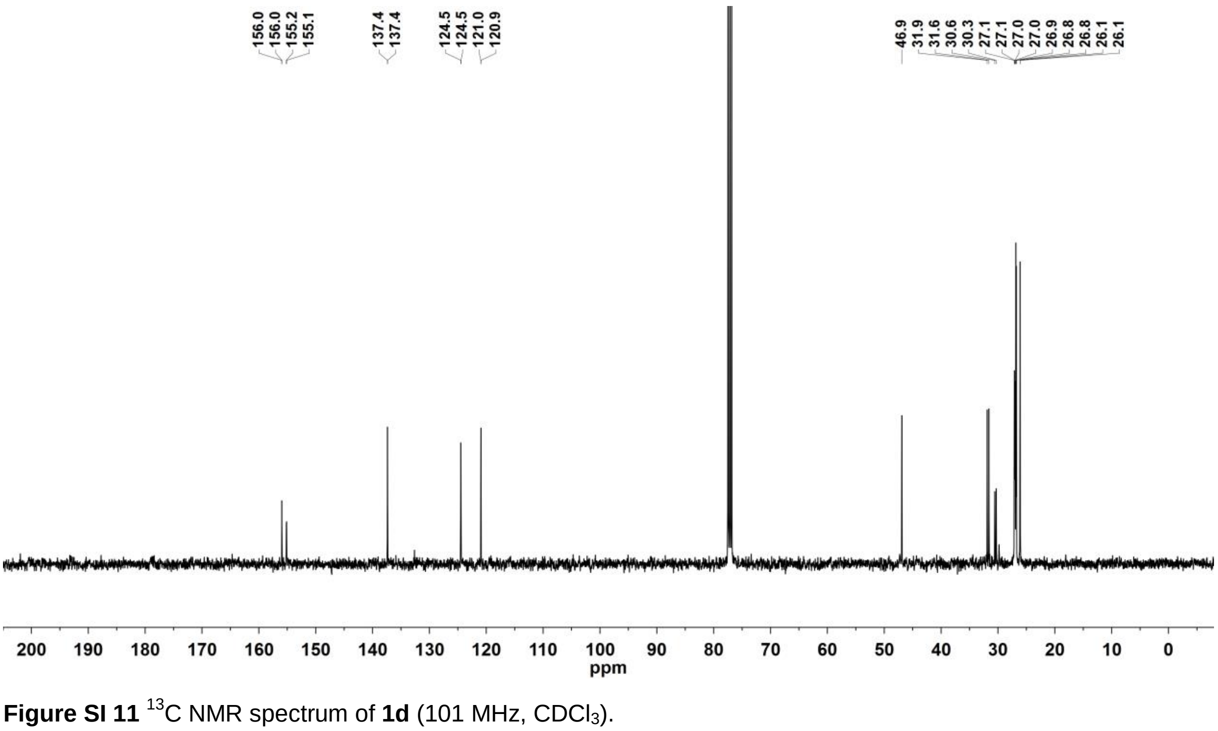

Figure SI 11 ^{13}C NMR spectrum of **1d** (101 MHz, $CDCl_3$).

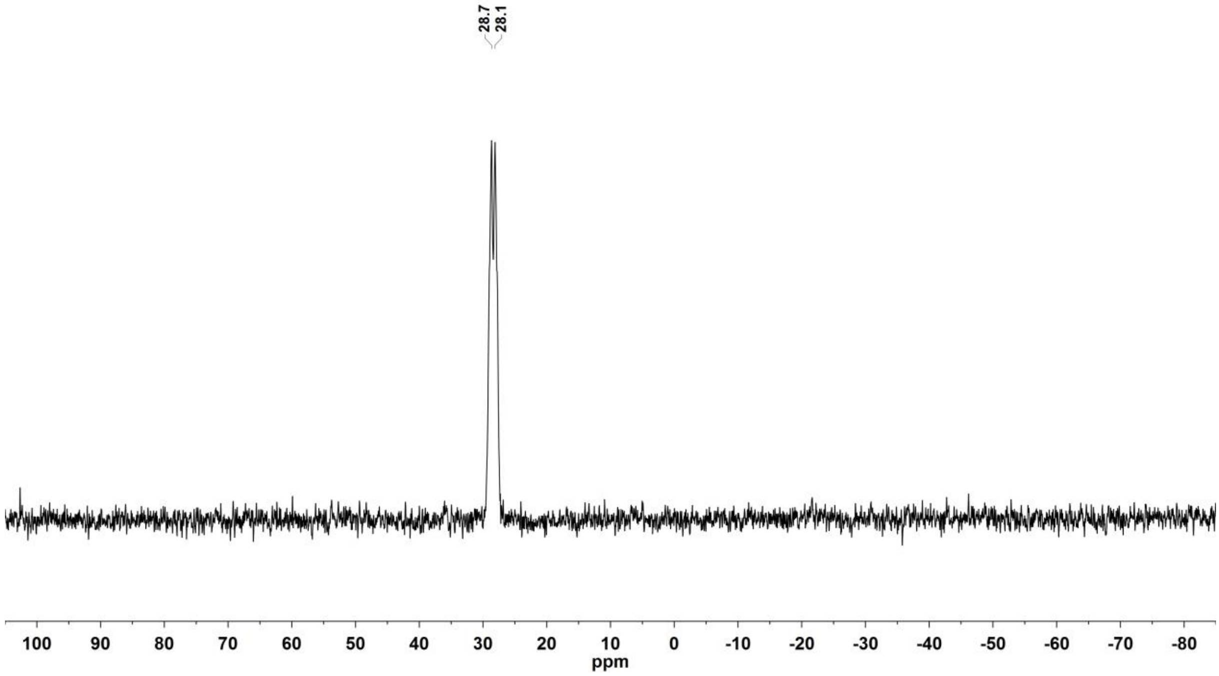

Figure SI 12 ^{31}P NMR spectrum of **1d** (121 MHz, $CDCl_3$).

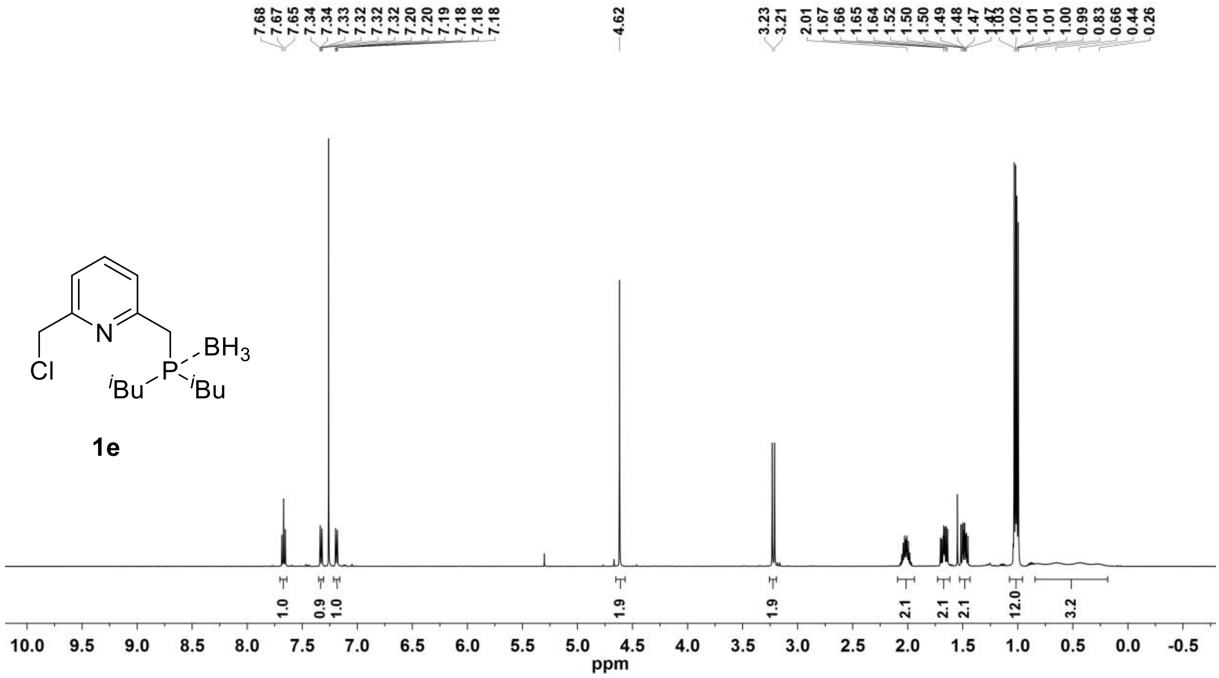

Figure SI 13 ^{1}H NMR spectrum of **1e** (500 MHz, $CDCl_3$).

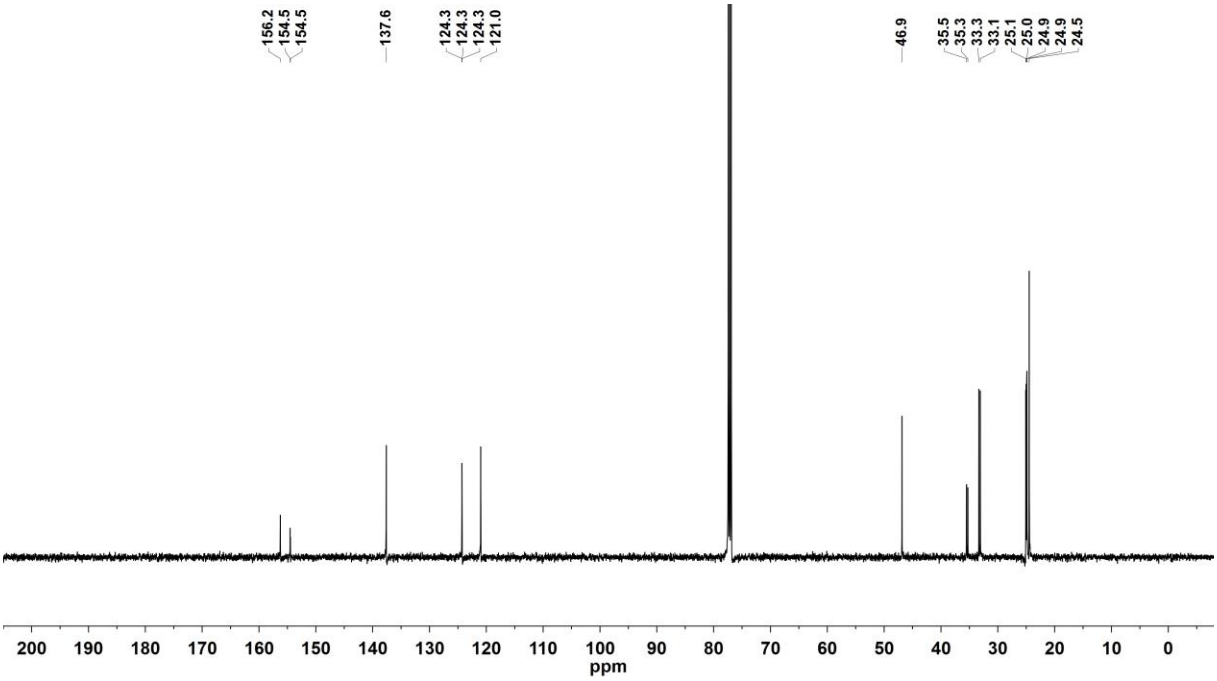

Figure SI 14 ^{13}C NMR spectrum of **1e** (126 MHz, $CDCl_3$).

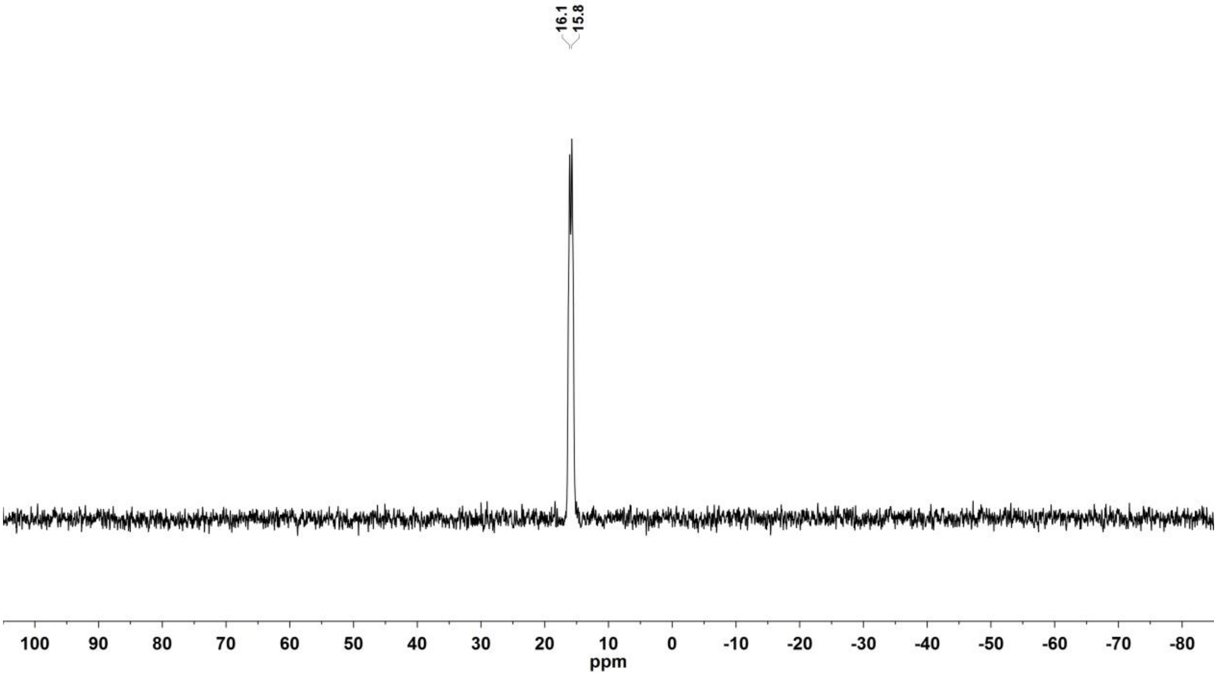

Figure SI 15 ^{31}P NMR spectrum of **1e** (202 MHz, $CDCl_3$).

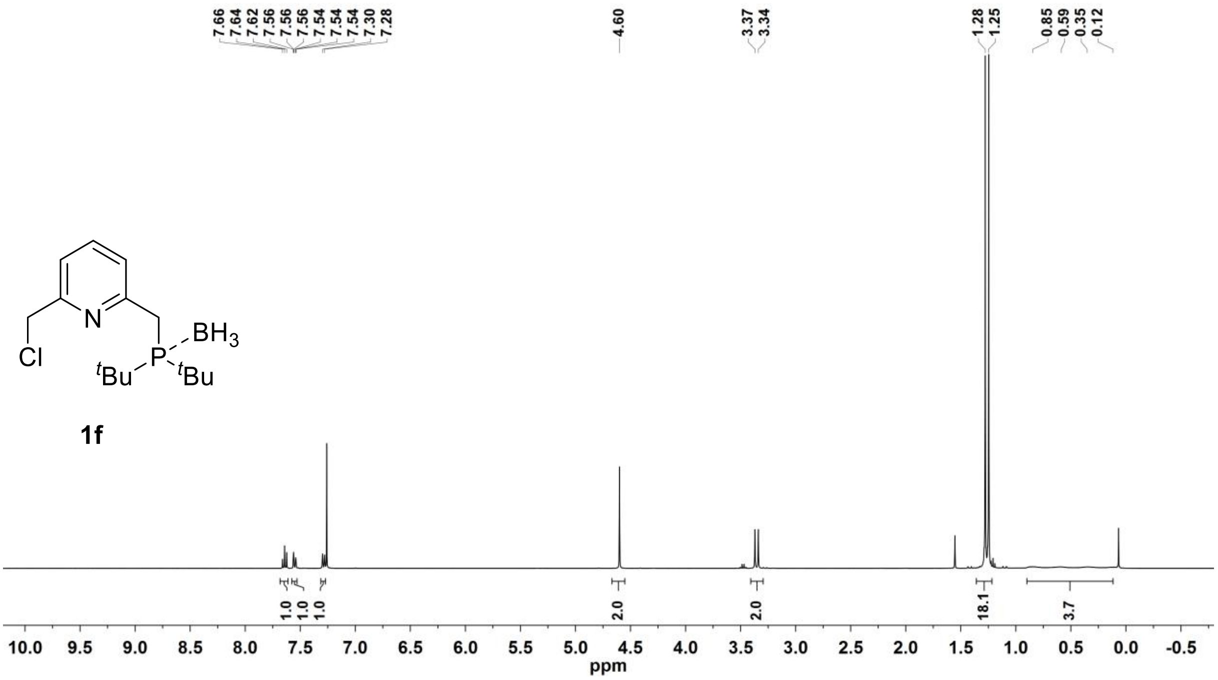

Figure SI 16 ^{1}H NMR spectrum of **1f** (400 MHz, $CDCl_3$).

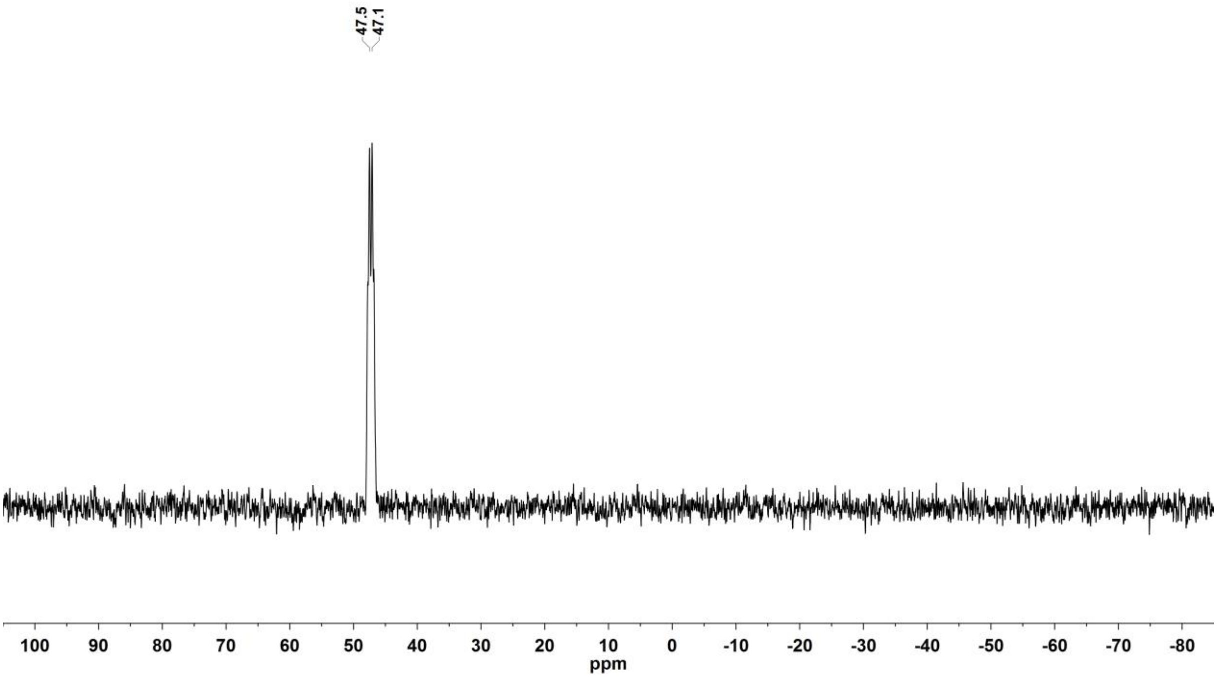

Figure SI 17 ^{31}P NMR spectrum of **1f** (162 MHz, $CDCl_3$).

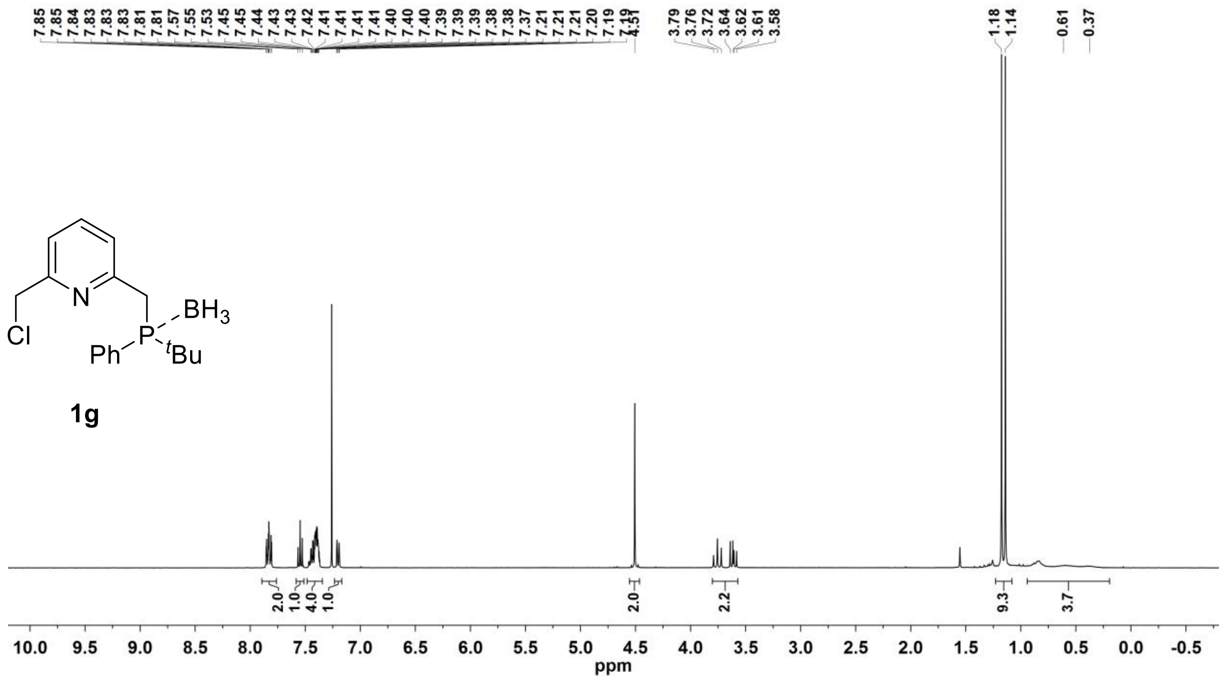

Figure SI 18 ^{1}H NMR spectrum of **1g** (400 MHz, $CDCl_3$).

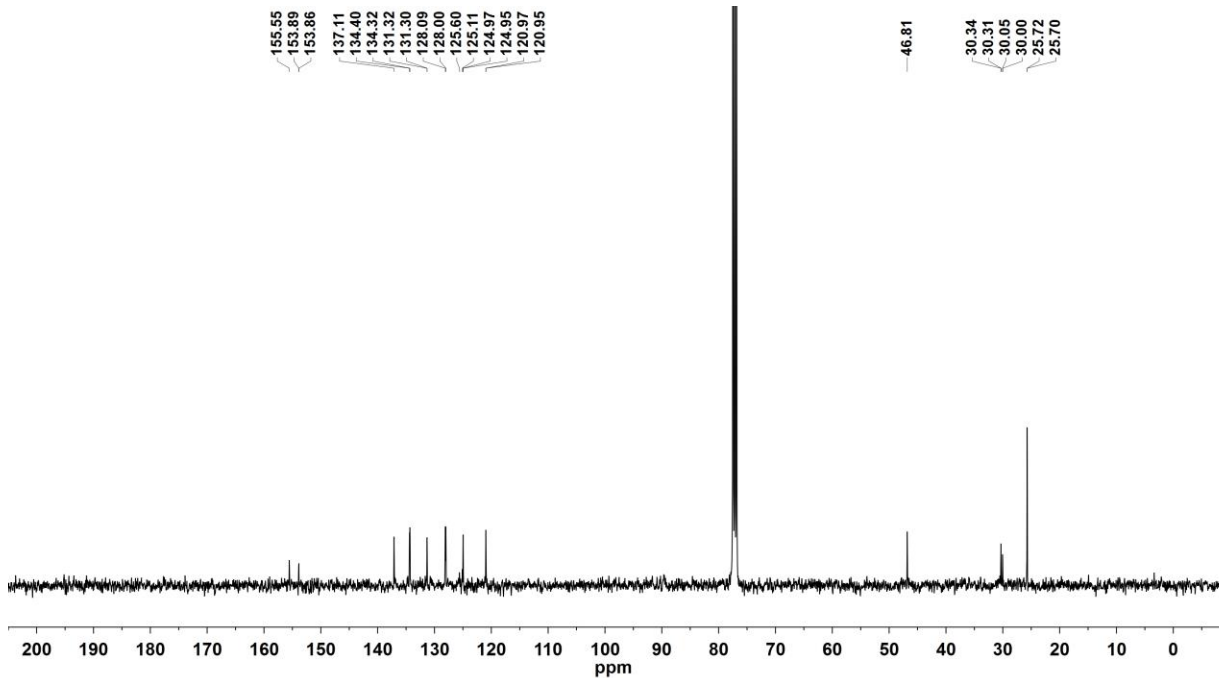

Figure SI 19 ^{13}C NMR spectrum of **1g** (101 MHz, $CDCl_3$).

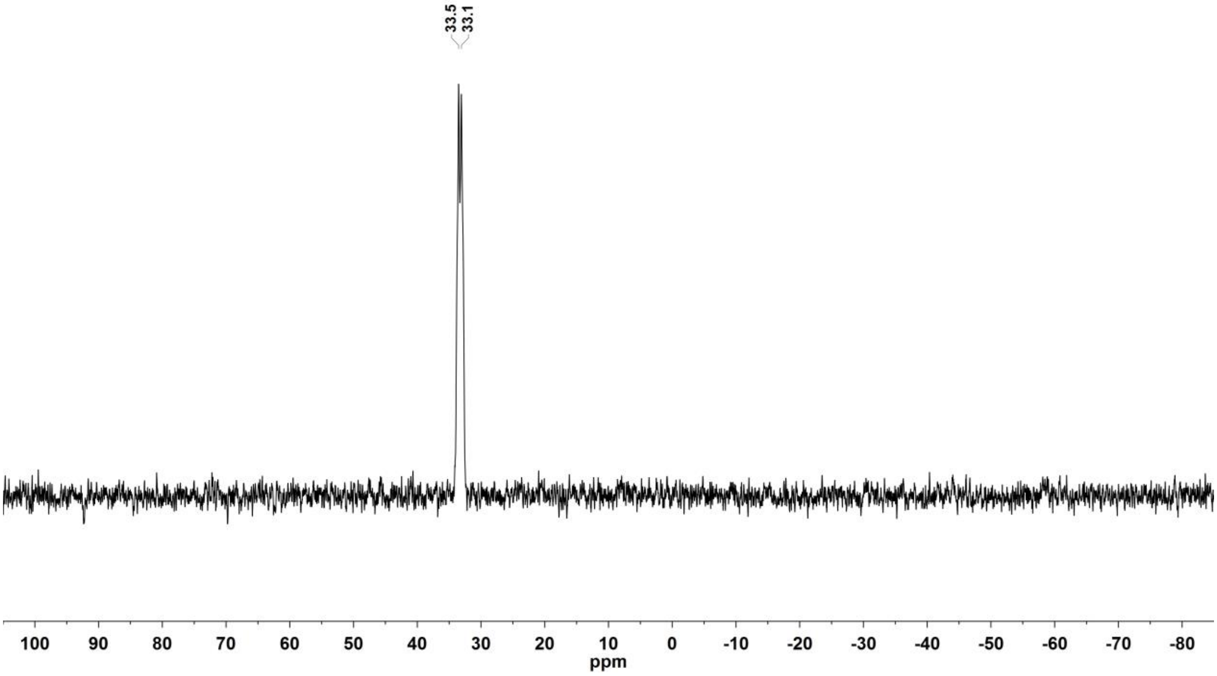

Figure SI 20 ^{31}P NMR spectrum of **1g** (162 MHz, $CDCl_3$).

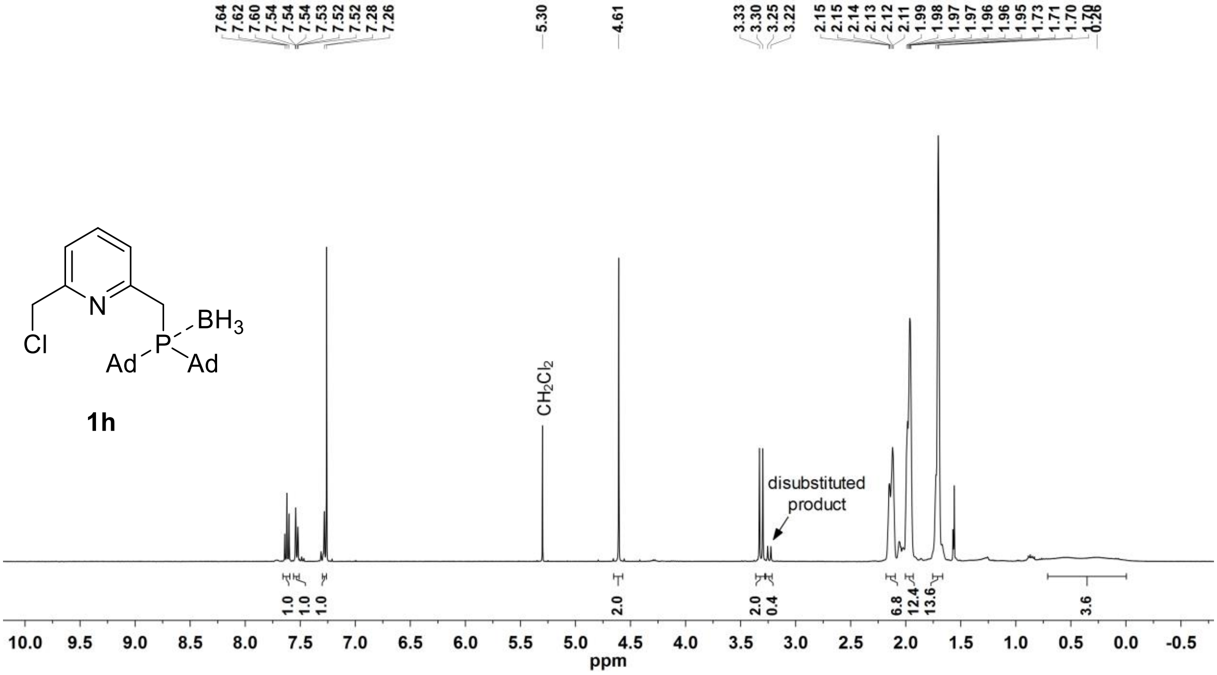

Figure SI 21 ^{1}H NMR spectrum of **1h** (400 MHz, $CDCl_3$).

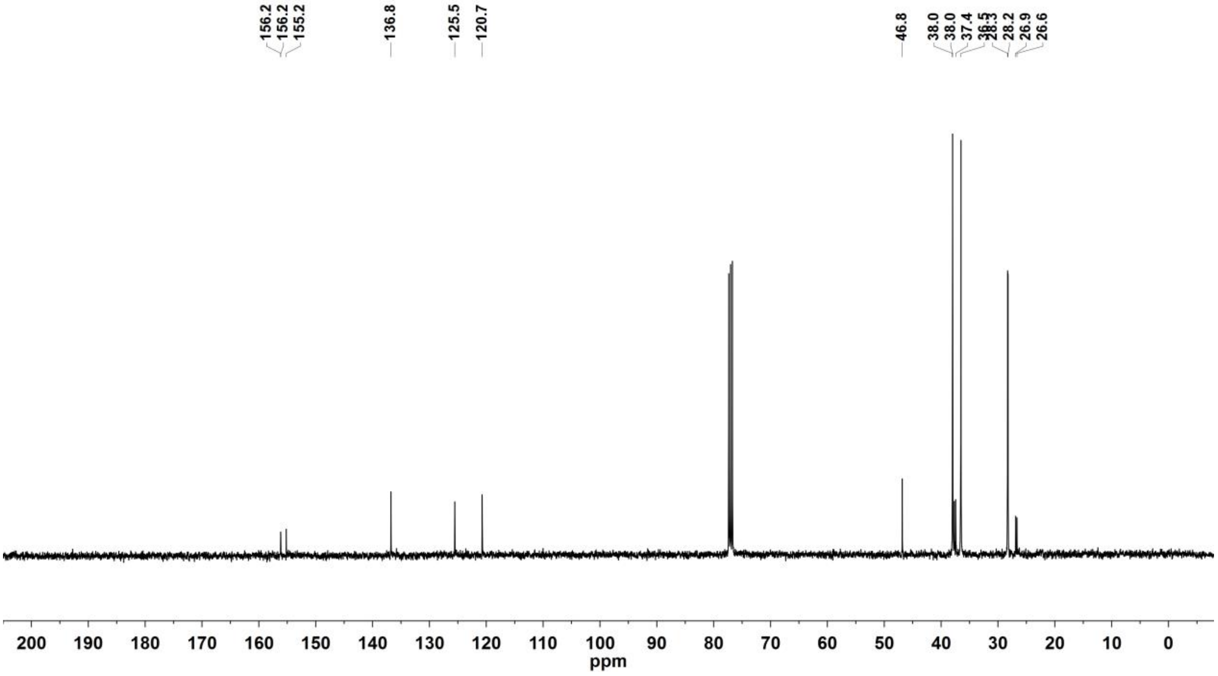

Figure SI 22 ^{13}C NMR spectrum of **1h** (101 MHz, $CDCl_3$).

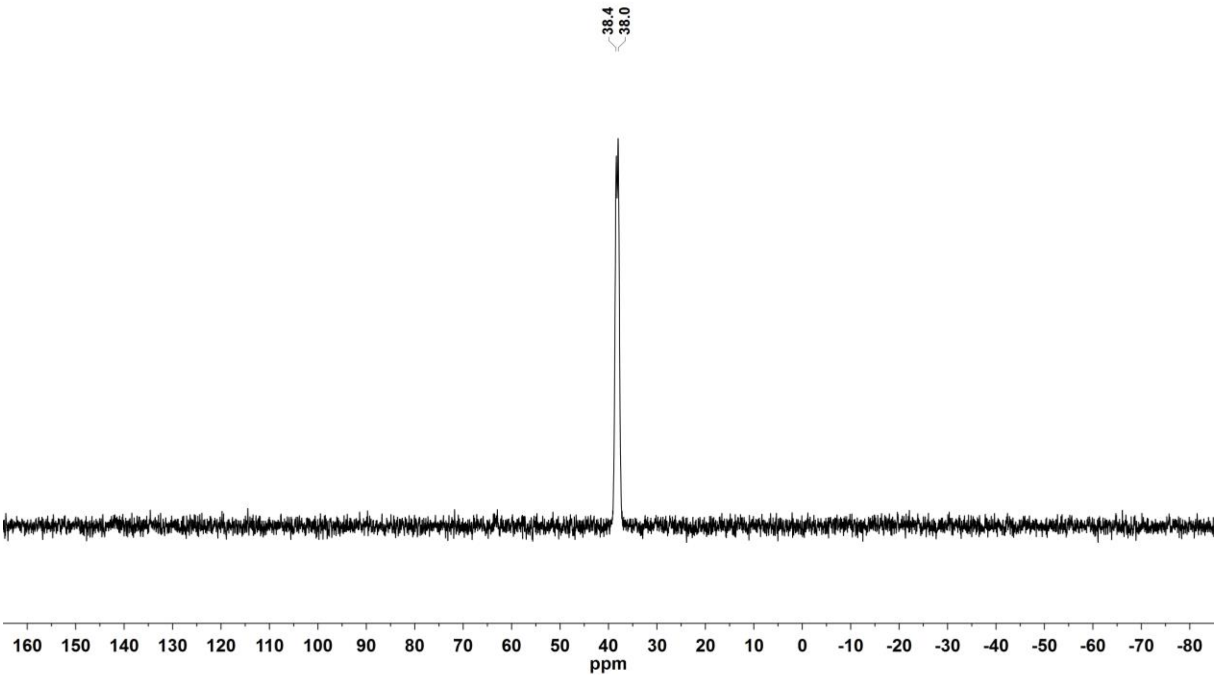

Figure SI 23 ^{31}P NMR spectrum of **1h** (202 MHz, $CDCl_3$).

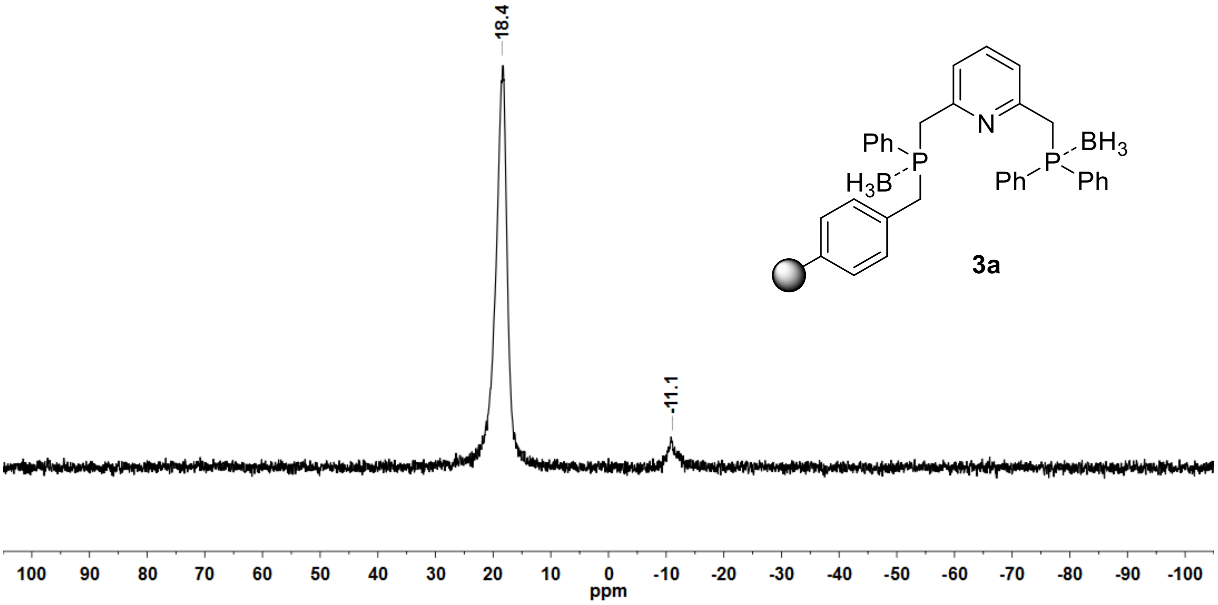

Figure SI 24 Gel-phase ^{31}P NMR of **3a** and 4% of free -*P*Ph$_2$ (162 MHz, THF).

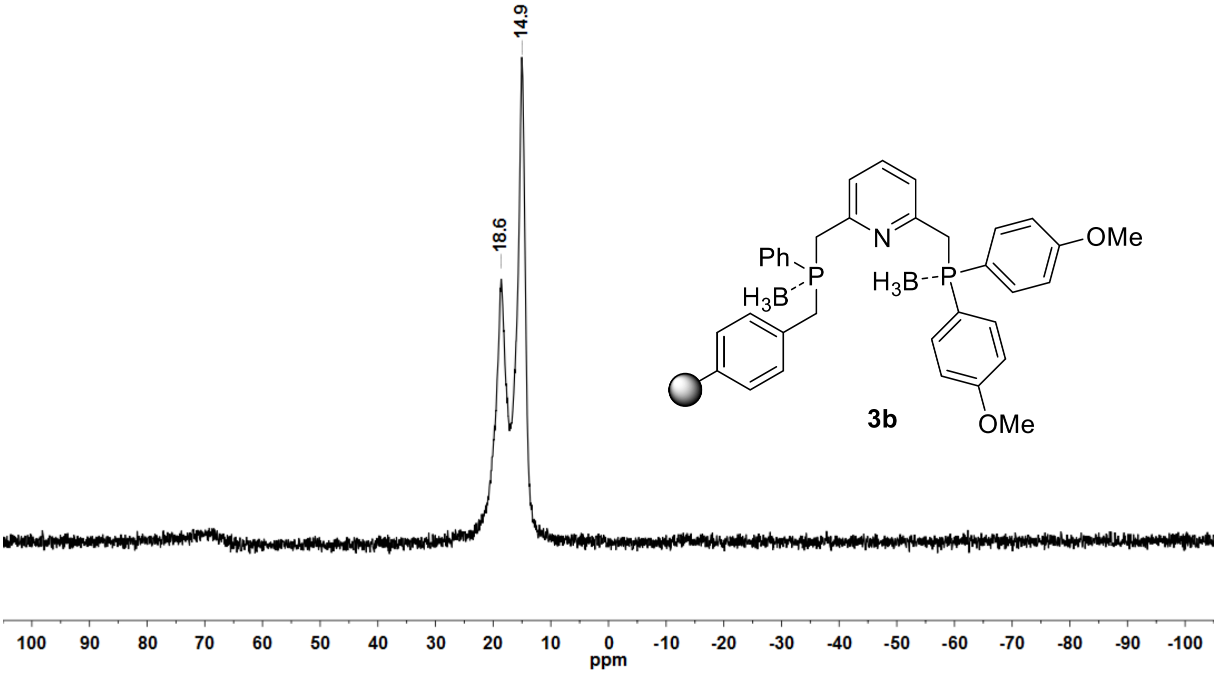

Figure SI 25 Gel-phase ^{31}P NMR of **3b** (162 MHz, THF).

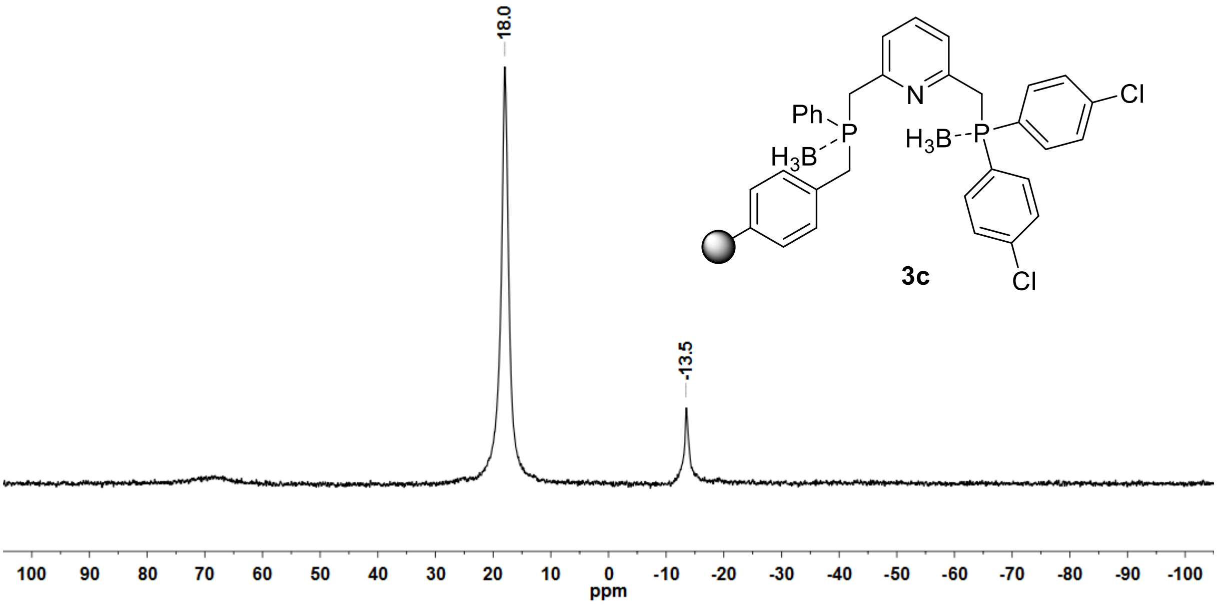

Figure SI 26 Gel-phase ^{31}P NMR of **3c** and 11% of free –*P*(*p*-4-C_6H_4)$_2$ (121 MHz, THF:C_6D_6 6:1).

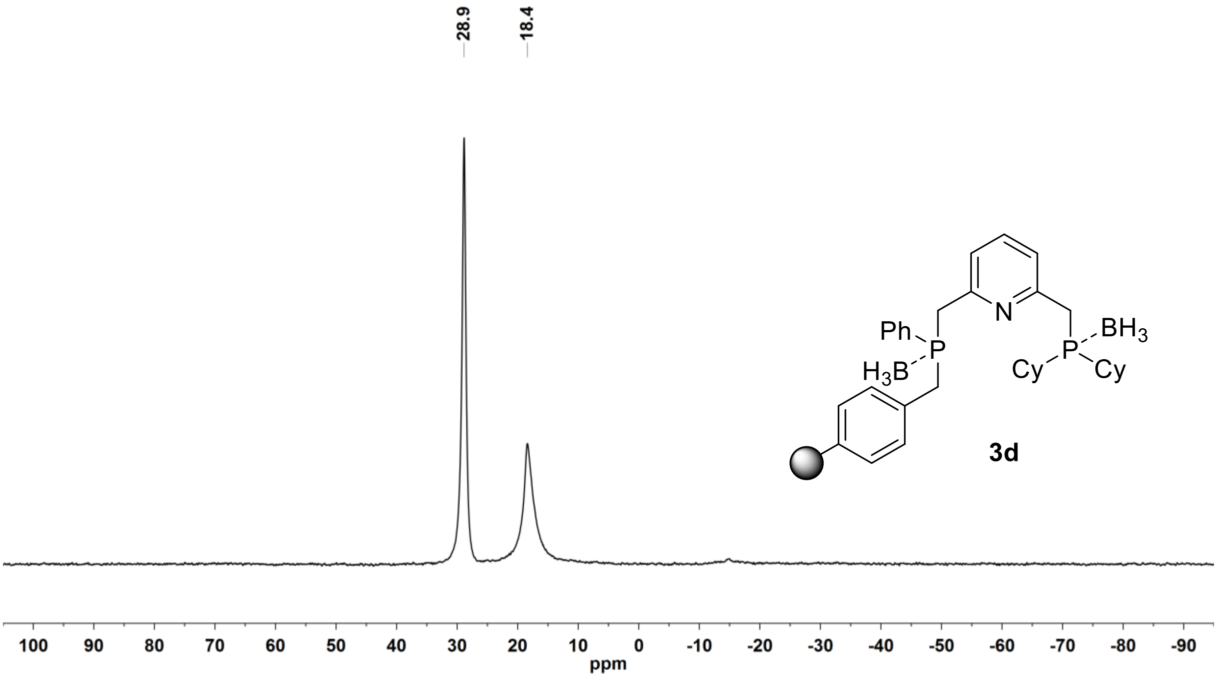

Figure SI 27 Gel-phase ^{31}P NMR of **3d** (121 MHz, THF:C_6D_6 6:1).

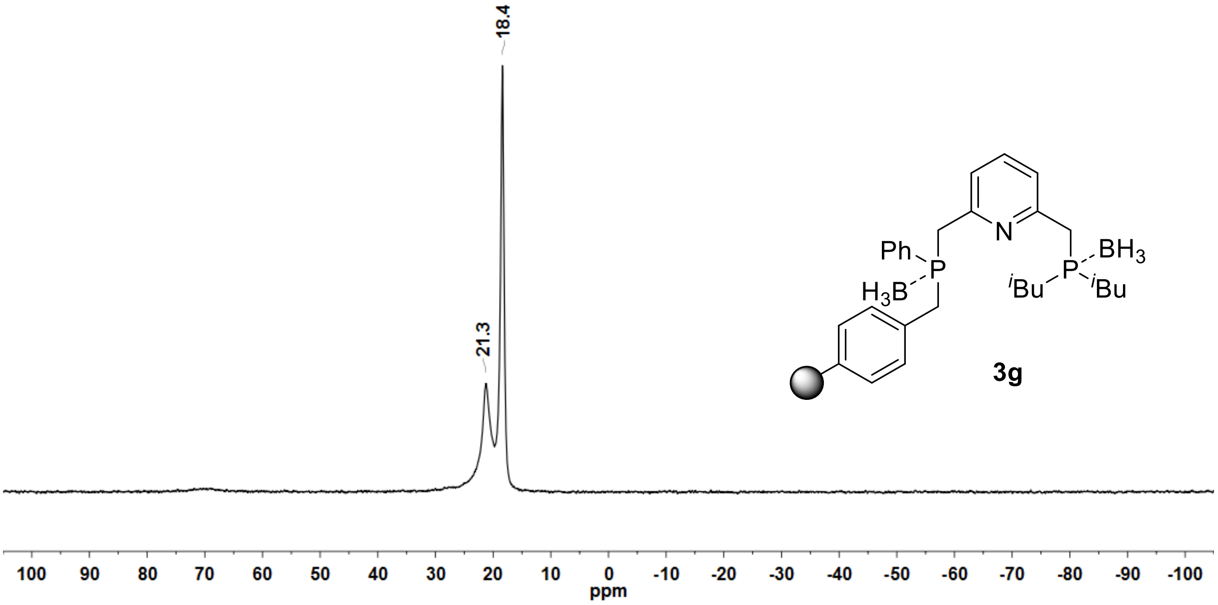

Figure SI 28 Gel-phase ^{31}P NMR of **3e** (162 MHz, THF).

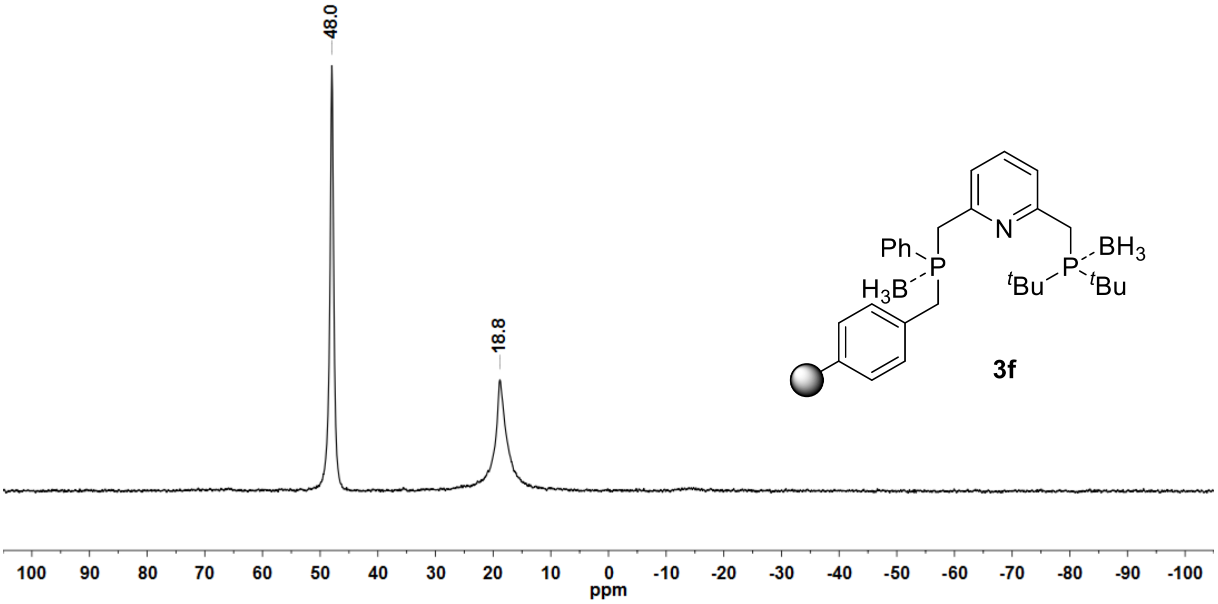

Figure SI 29 Gel-phase ^{31}P NMR of **3f** (162 MHz, THF).

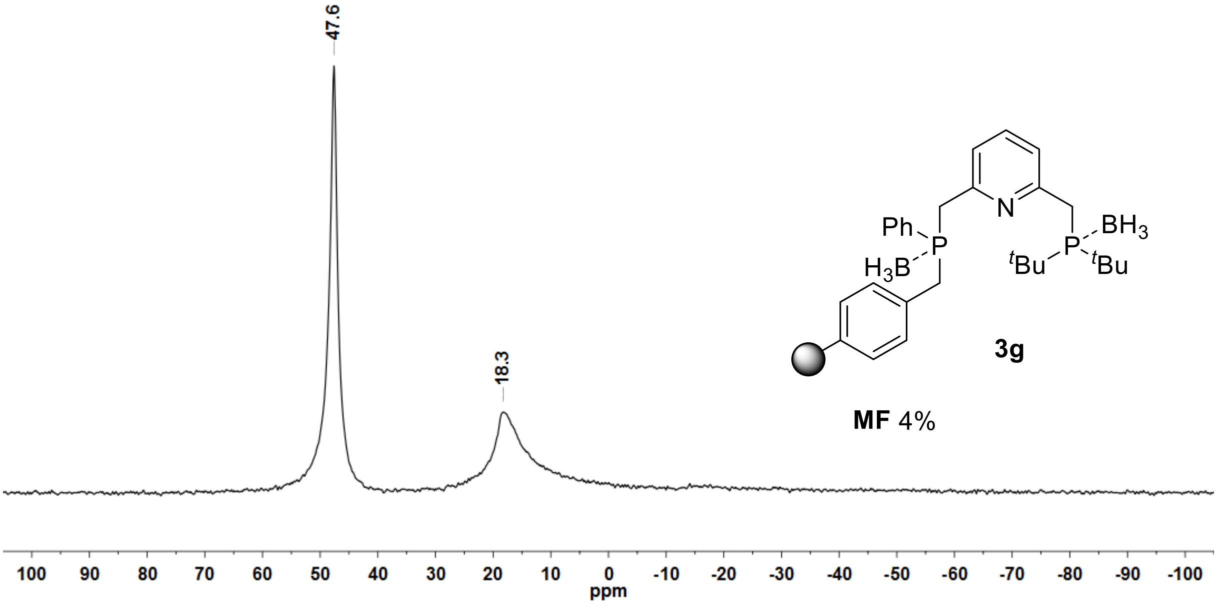

Figure SI 30 Gel-phase ^{31}P NMR of **3g** (121 MHz, THF:C_6D_6 6:1).

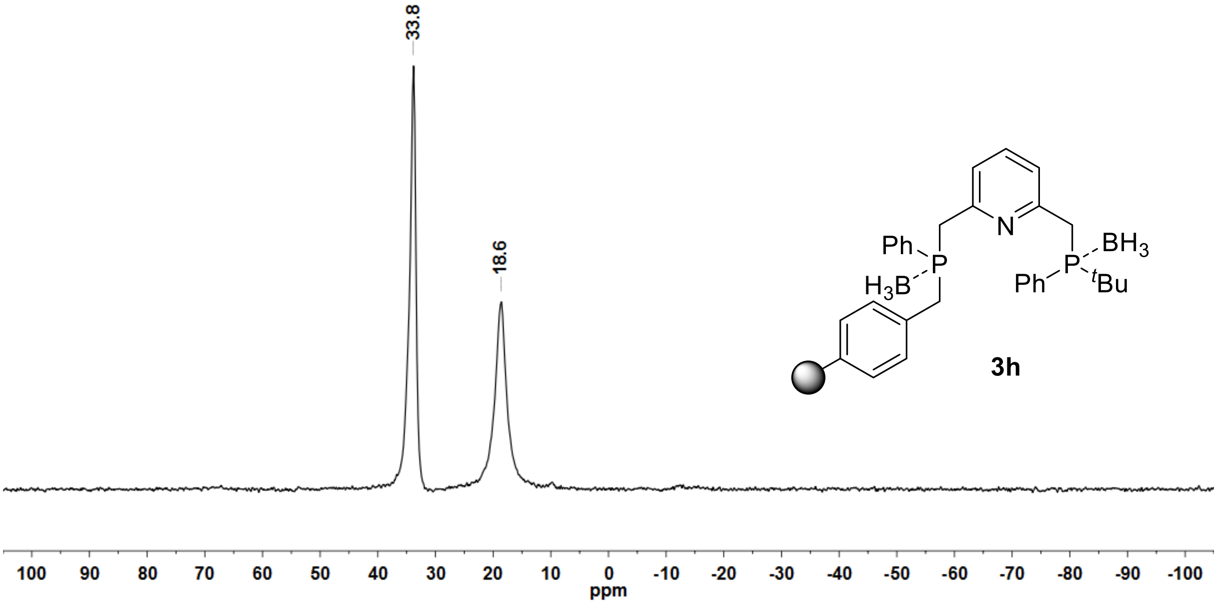

Figure SI 31 Gel-phase ^{31}P NMR of **3h** (162 MHz, THF).

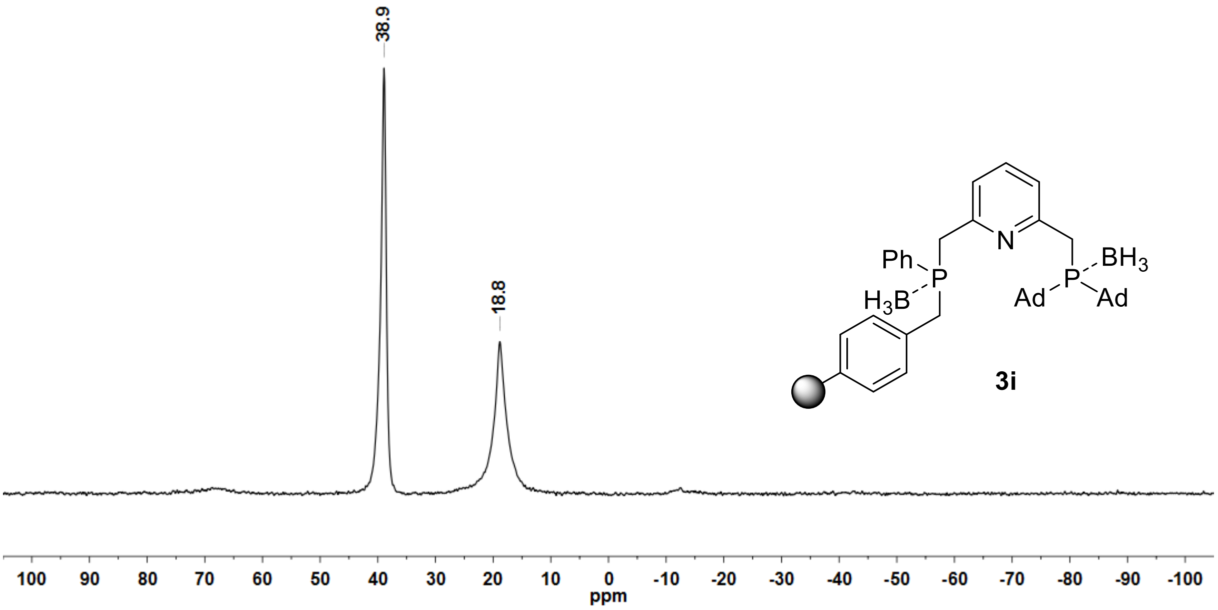

Figure SI 32 Gel-phase ^{31}P NMR of **3i** (162 MHz, THF).

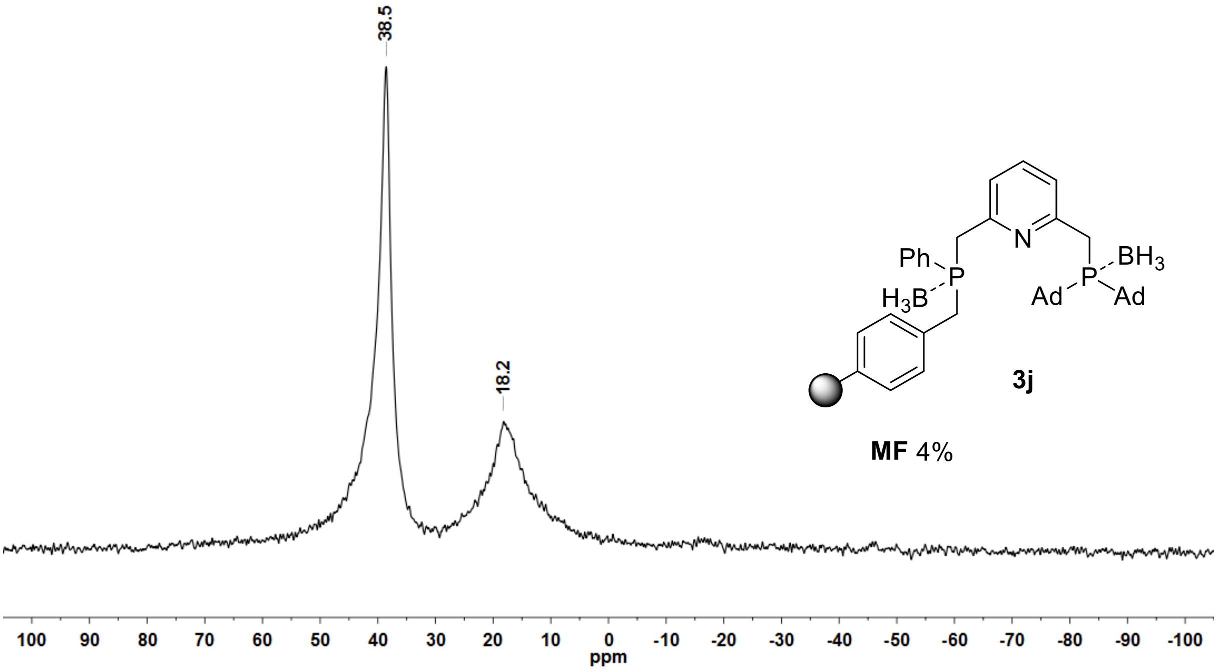

Figure SI 33 Gel-phase ^{31}P NMR of **3j** (121 MHz, THF:C_6D_6 2:1).

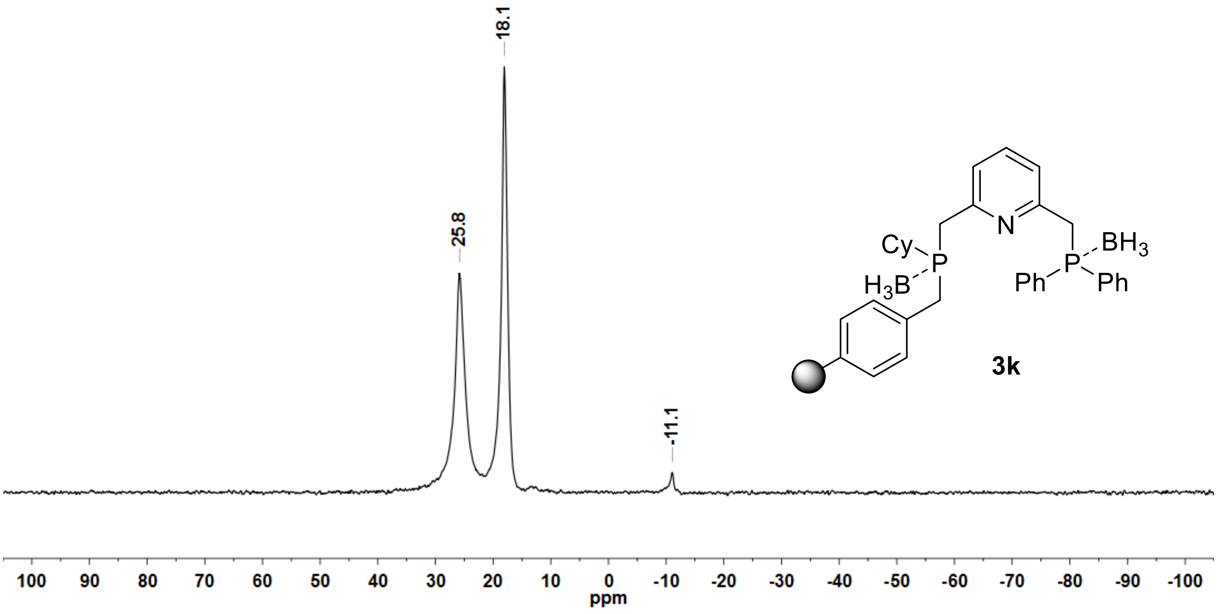

Figure SI 34 Gel-phase ^{31}P NMR of **3k** and 2% of free -*P*Ph$_2$ (162 MHz, THF).

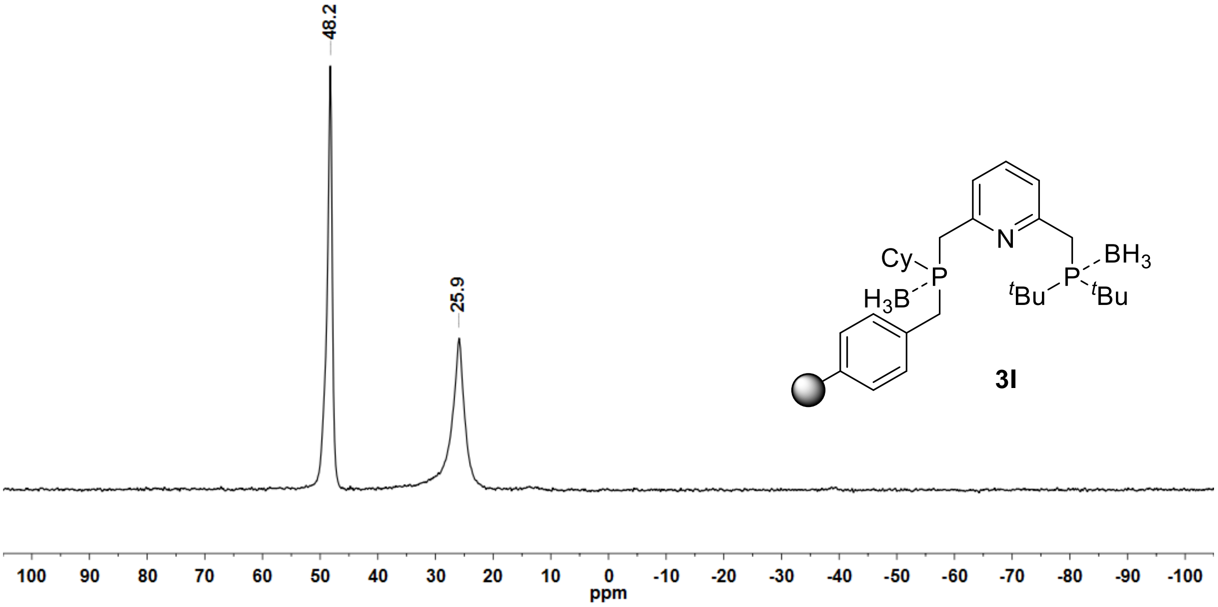

Figure SI 35 Gel-phase ^{31}P NMR of **3l** (162 MHz, THF).

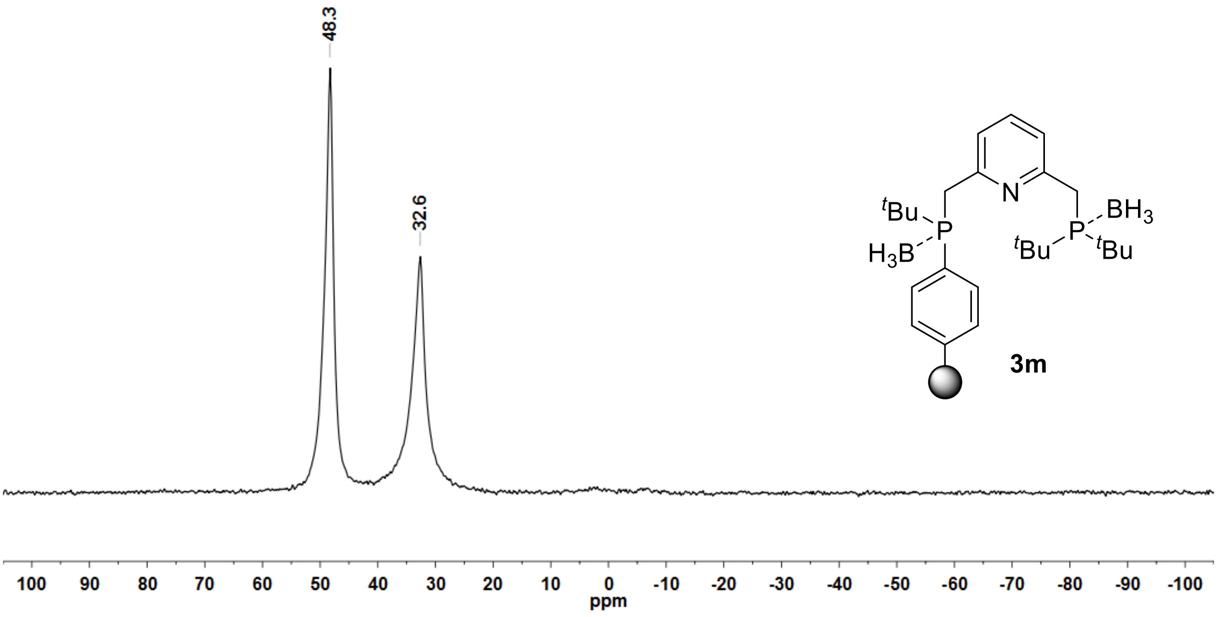

Figure SI 36 Gel-phase ^{31}P NMR of **3m** (162 MHz, THF).

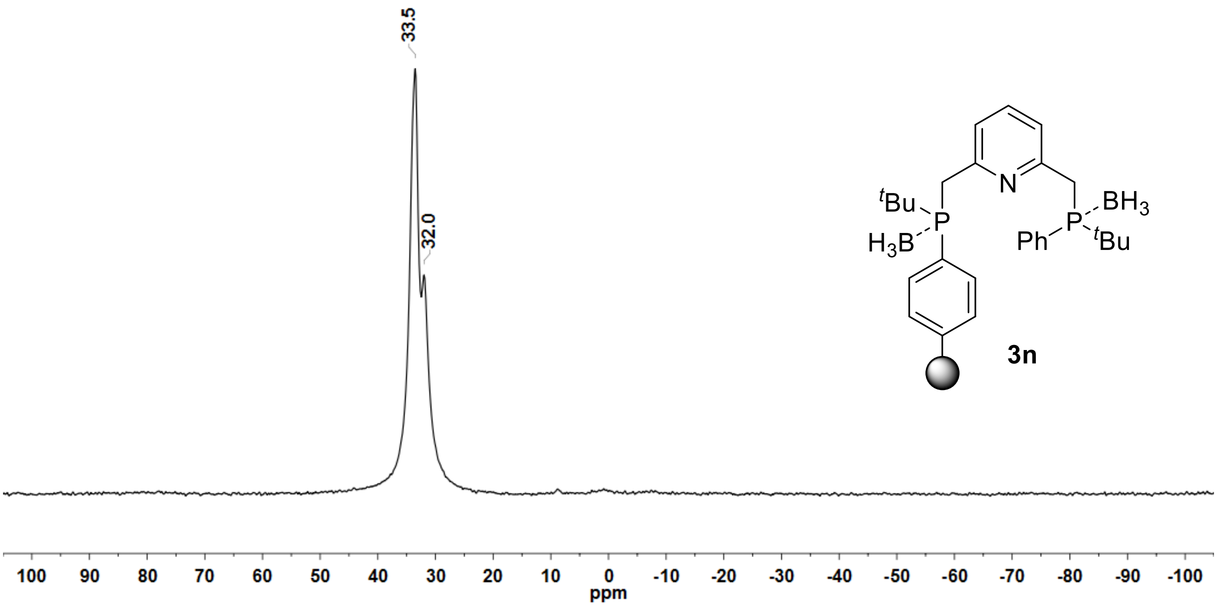

Figure SI 37 Gel-phase ^{31}P NMR of **3n** (162 MHz, THF).

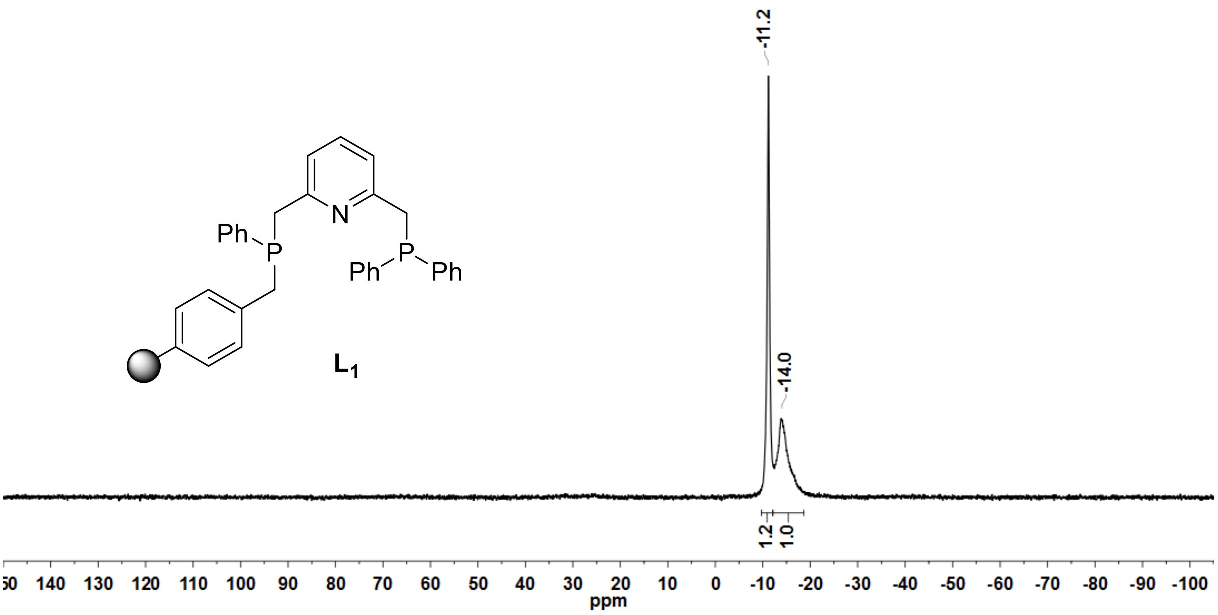

Figure SI 38 Gel-phase ^{31}P NMR of $\mathbf{L_1}$ (162 MHz, THF).

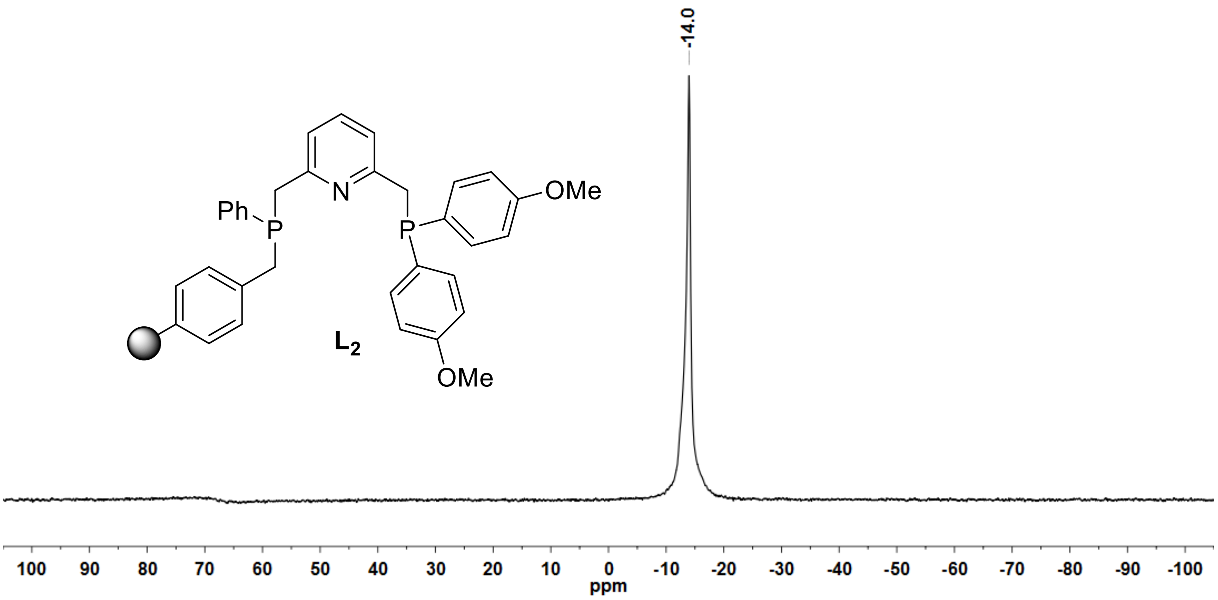

Figure SI 39 Gel-phase ^{31}P NMR of $\mathbf{L_2}$ (162 MHz, THF).

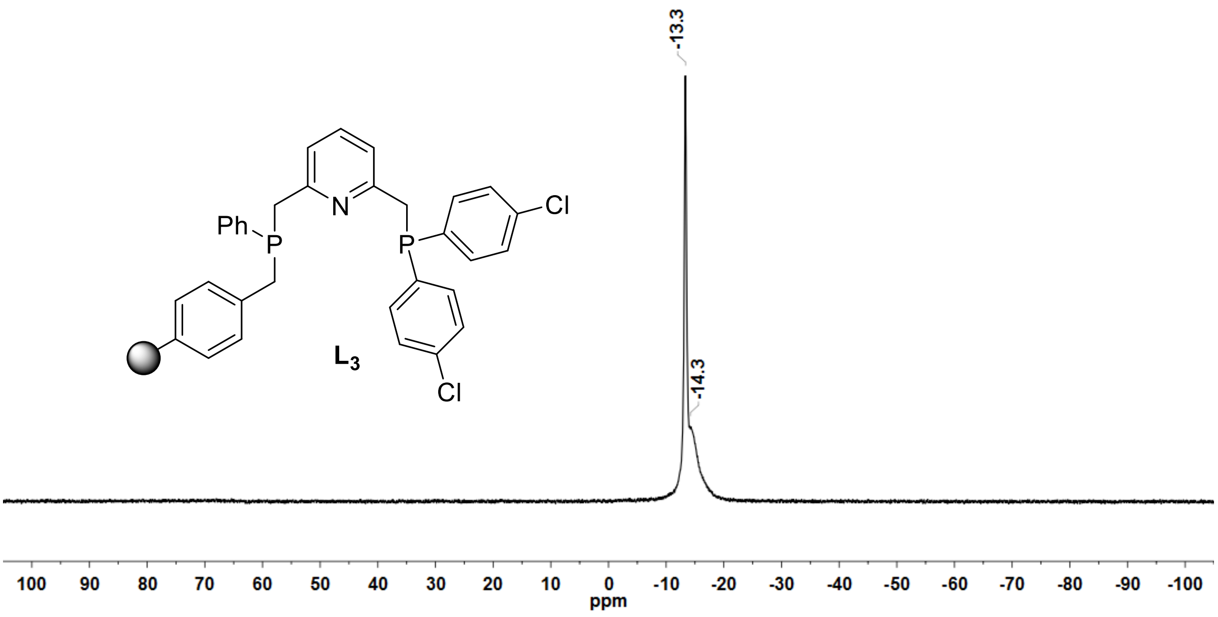

Figure SI 40 Gel-phase ^{31}P NMR of **L$_3$** (121 MHz, THF:C_6D_6 6:1).

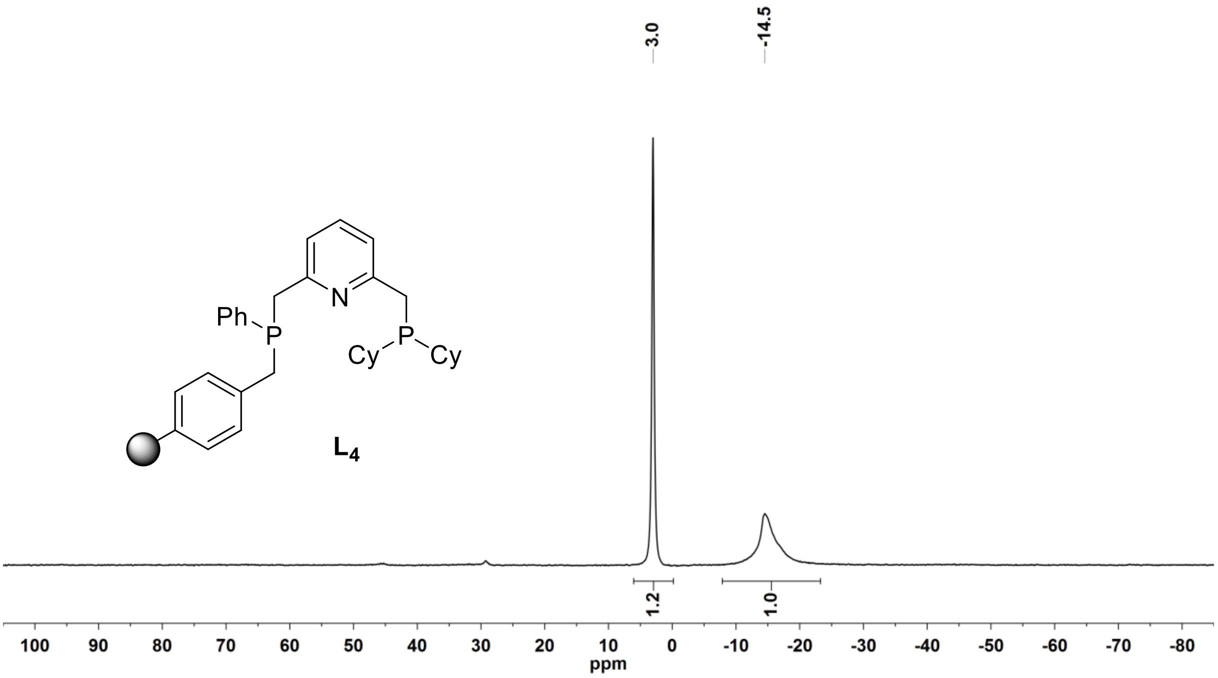

Figure SI 41 Gel-phase ^{31}P NMR of **L$_4$** (121 MHz, THF:C_6D_6 6:1).

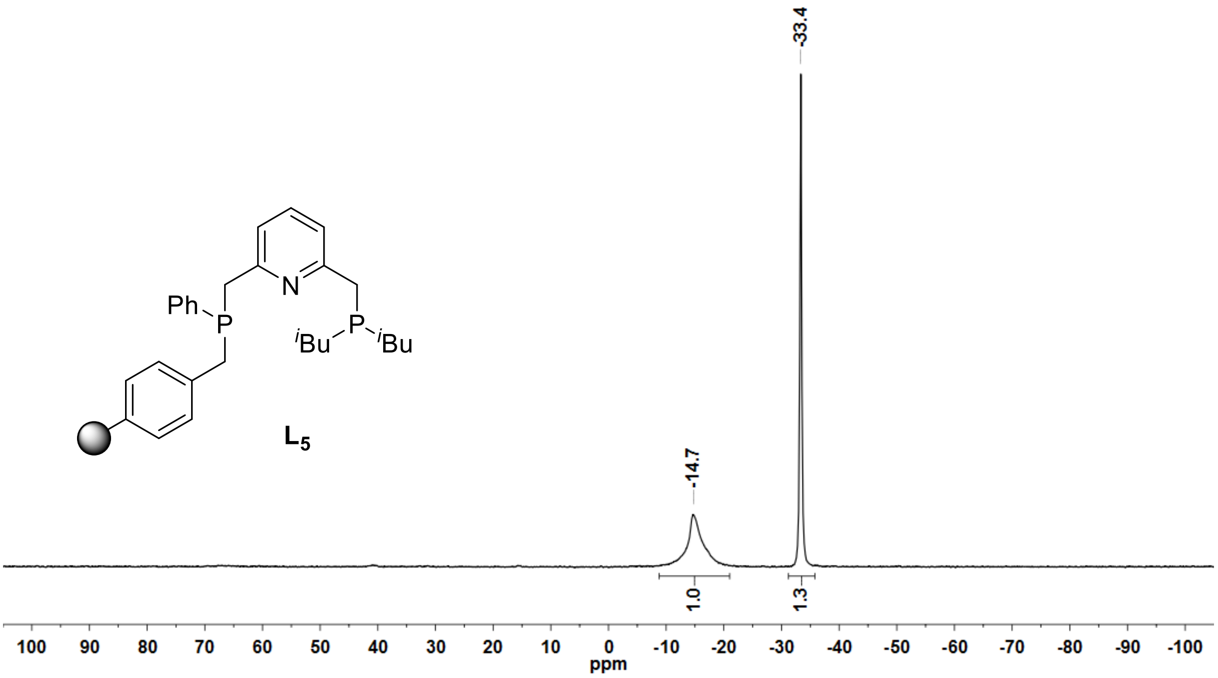

Figure SI 42 Gel-phase ^{31}P NMR of $\mathbf{L_5}$ (162 MHz, THF).

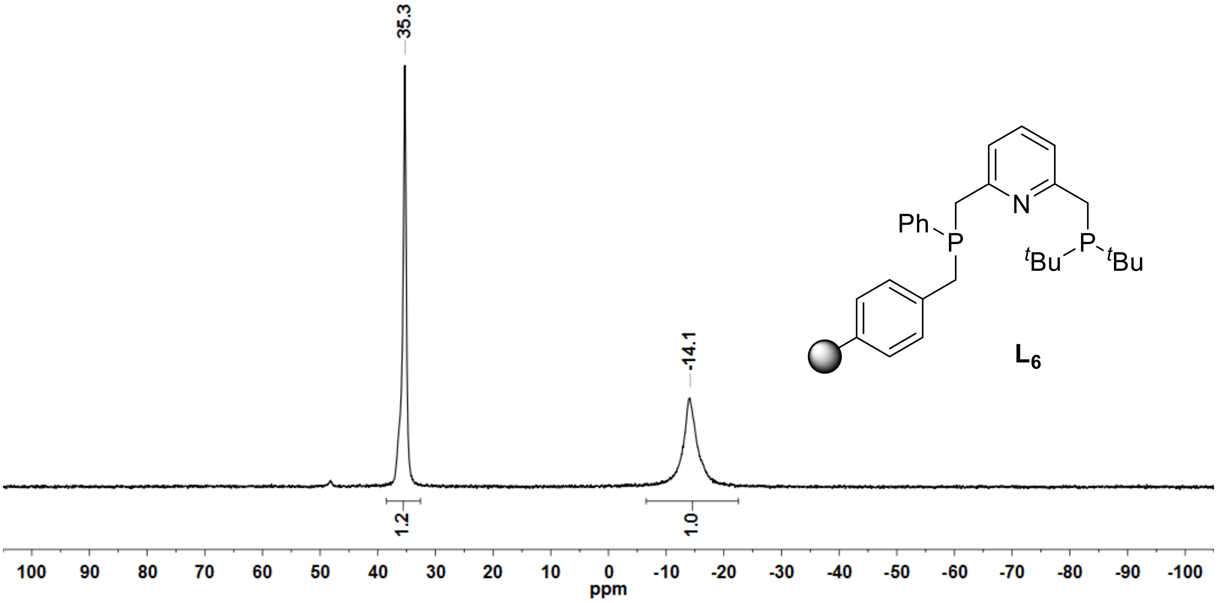

Figure SI 43 Gel-phase ^{31}P NMR of $\mathbf{L_6}$ (162 MHz, THF).

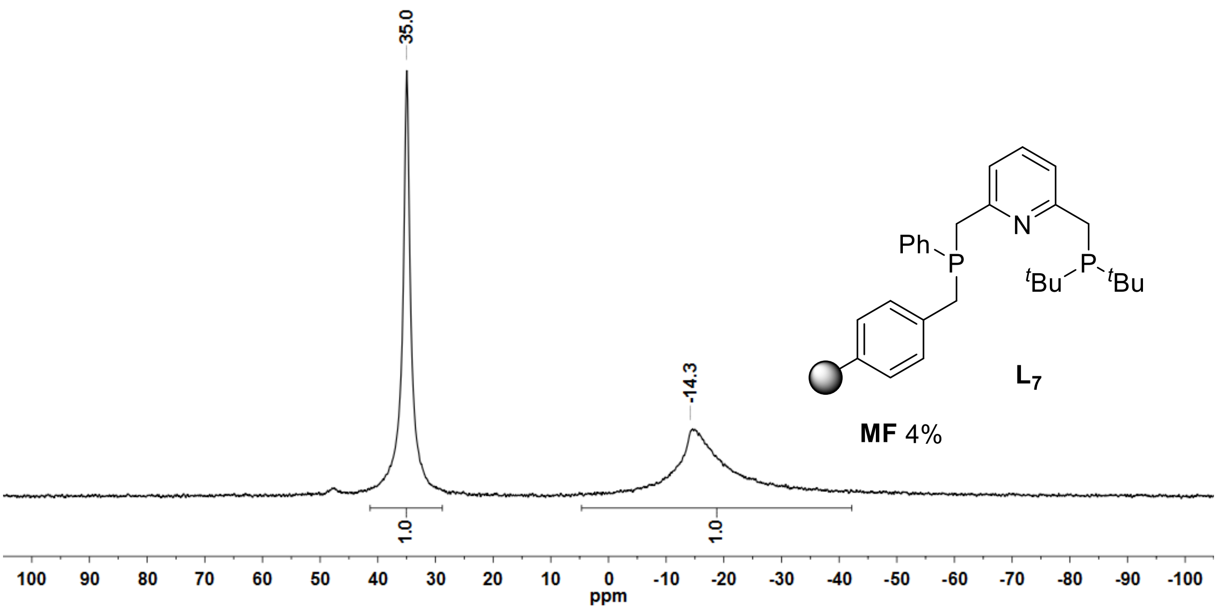

Figure SI 44 Gel-phase ^{31}P NMR of $\mathbf{L_7}$ (121 MHz, THF:C_6D_6 6:1).

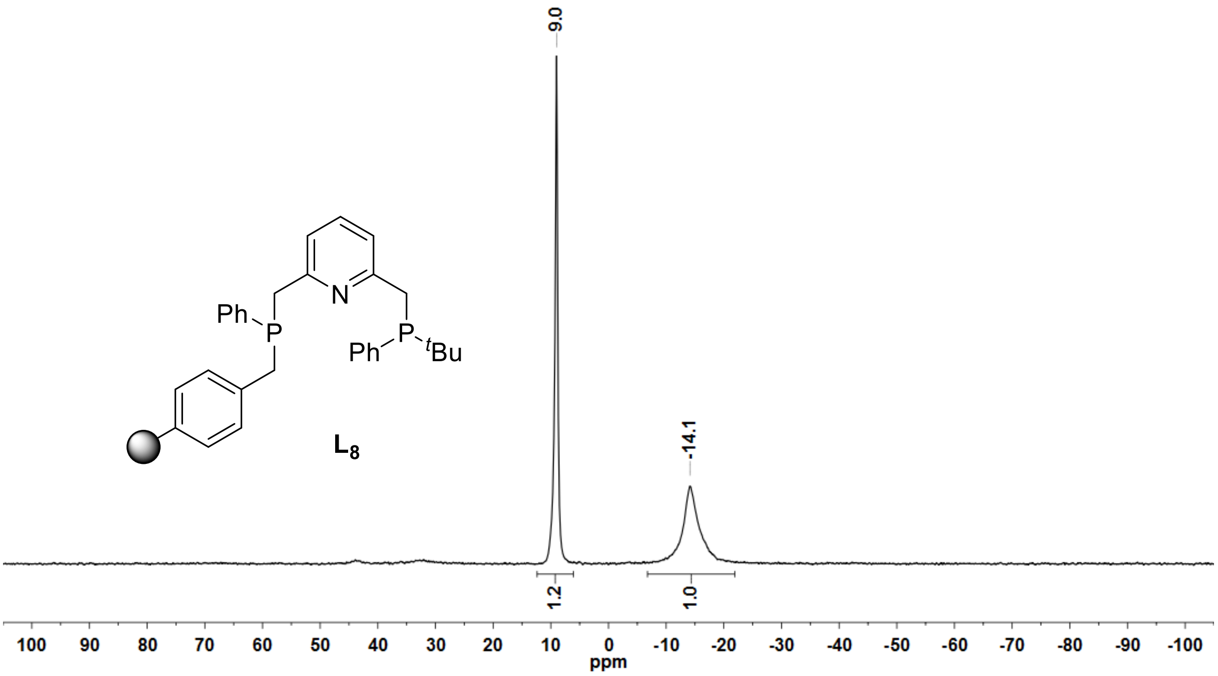

Figure SI 45 Gel-phase ^{31}P NMR of $\mathbf{L_8}$ (162 MHz, THF).

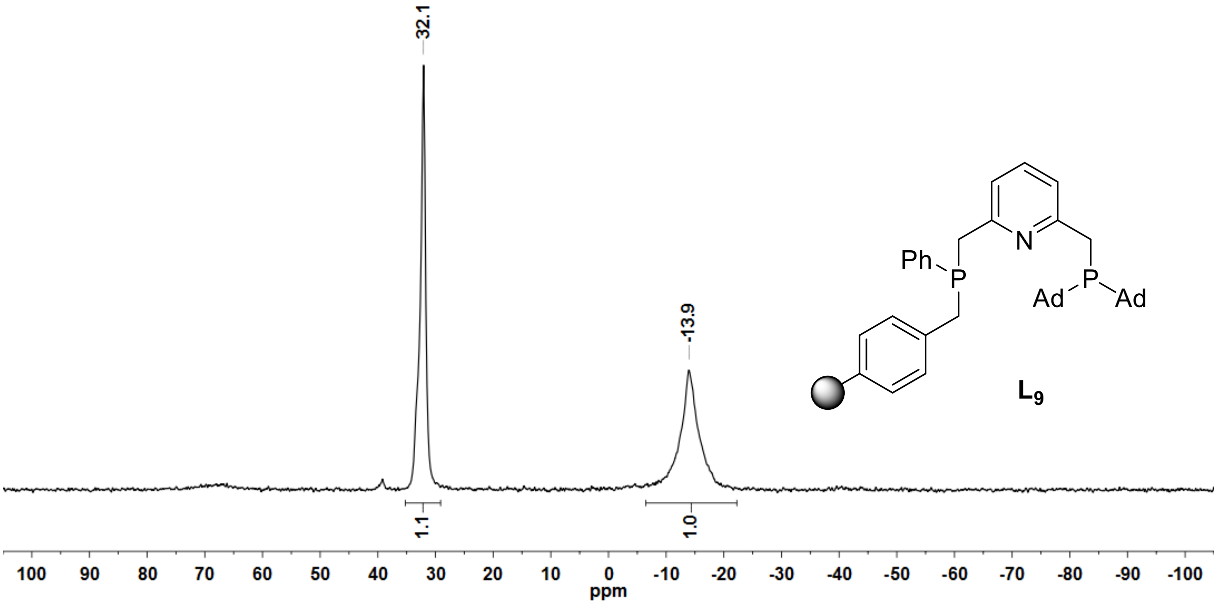

Figure SI 46 Gel-phase ^{31}P NMR of **L₉** (162 MHz, THF).

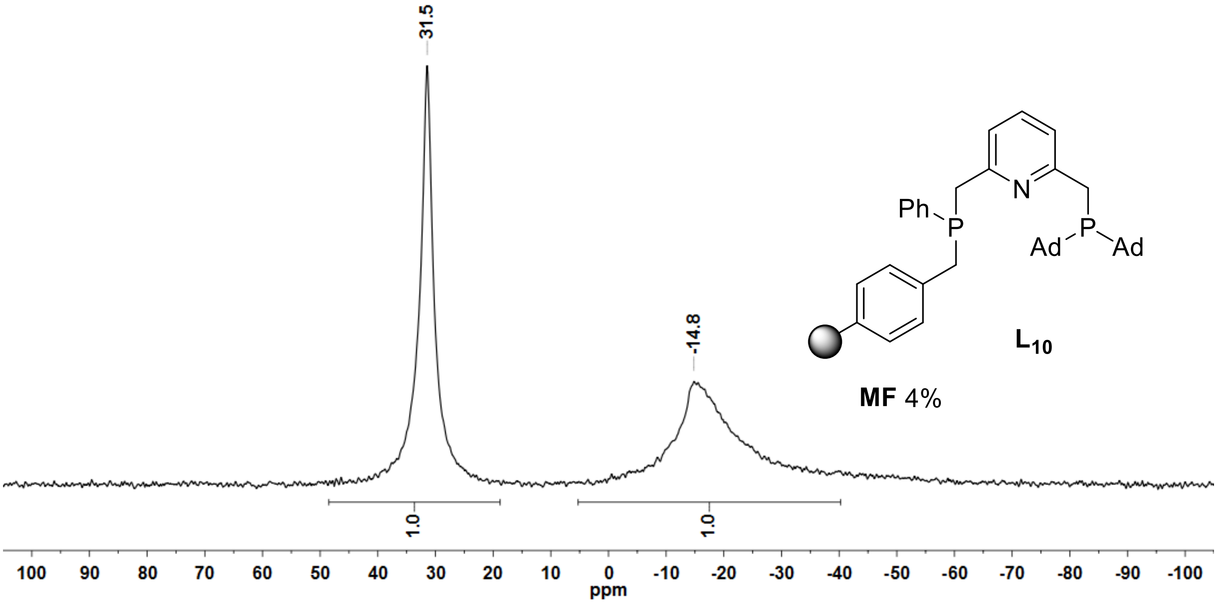

Figure SI 47 Gel-phase ^{31}P NMR of **L_{10}** (121 MHz, THF:C_6D_6 6:1).

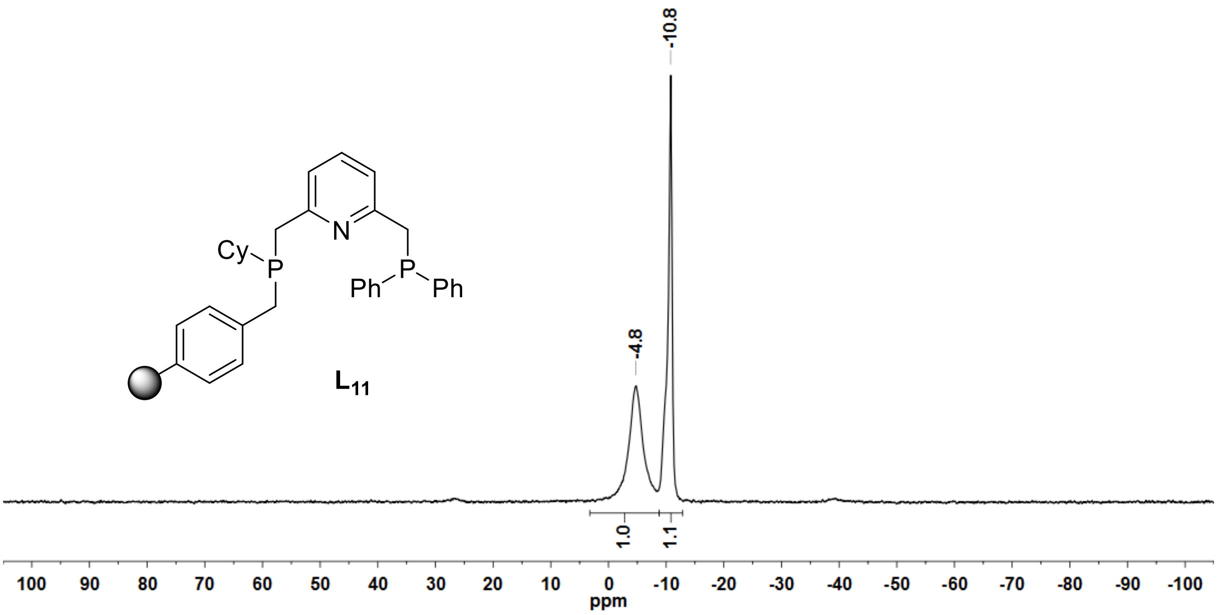

Figure SI 48 Gel-phase ^{31}P NMR of $\mathbf{L_{11}}$ (162 MHz, THF).

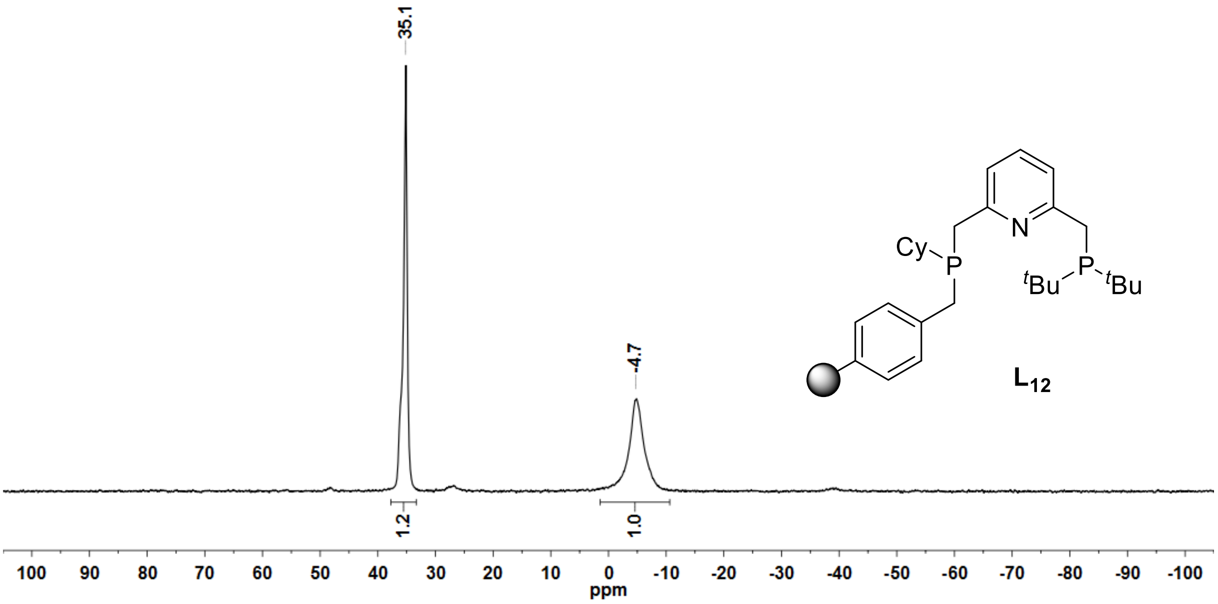

Figure SI 49 Gel-phase ^{31}P NMR of $\mathbf{L_{12}}$ (162 MHz, THF).

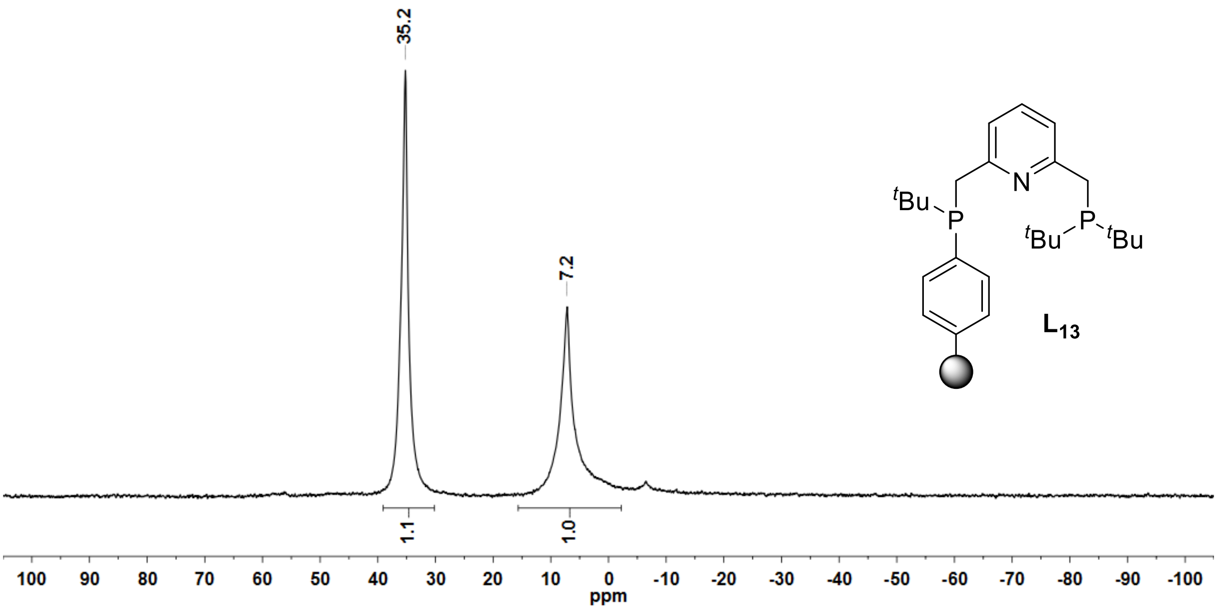

Figure SI 50 Gel-phase ^{31}P NMR of $\mathbf{L_{13}}$ (162 MHz, THF).

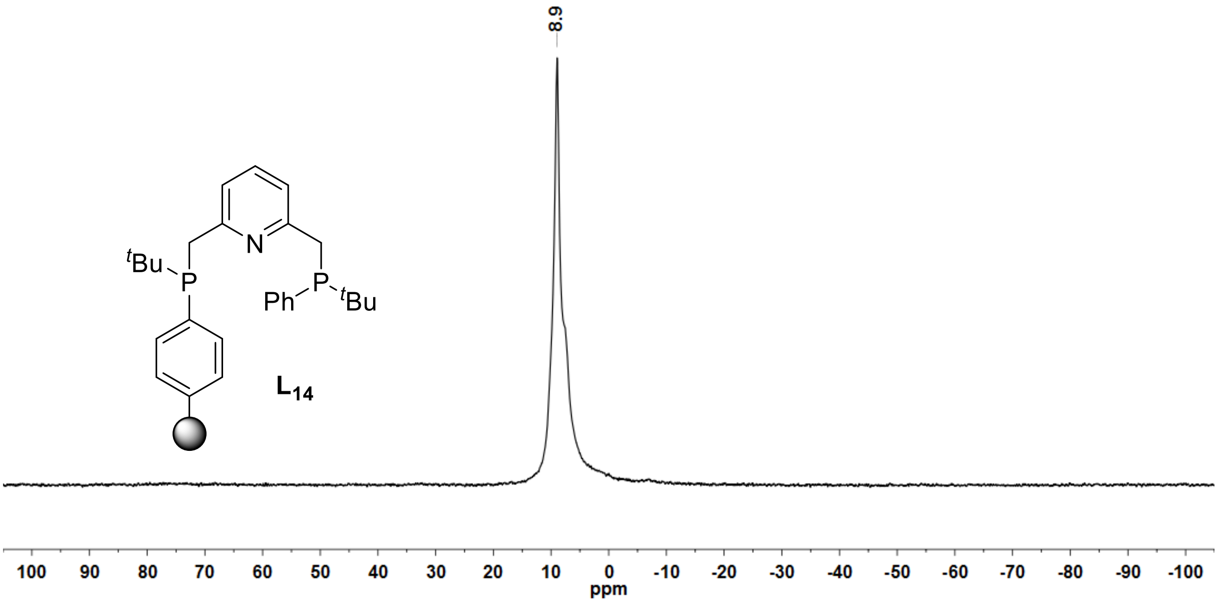

Figure SI 51 Gel-phase ^{31}P NMR of $\mathbf{L_{14}}$ (162 MHz, THF).

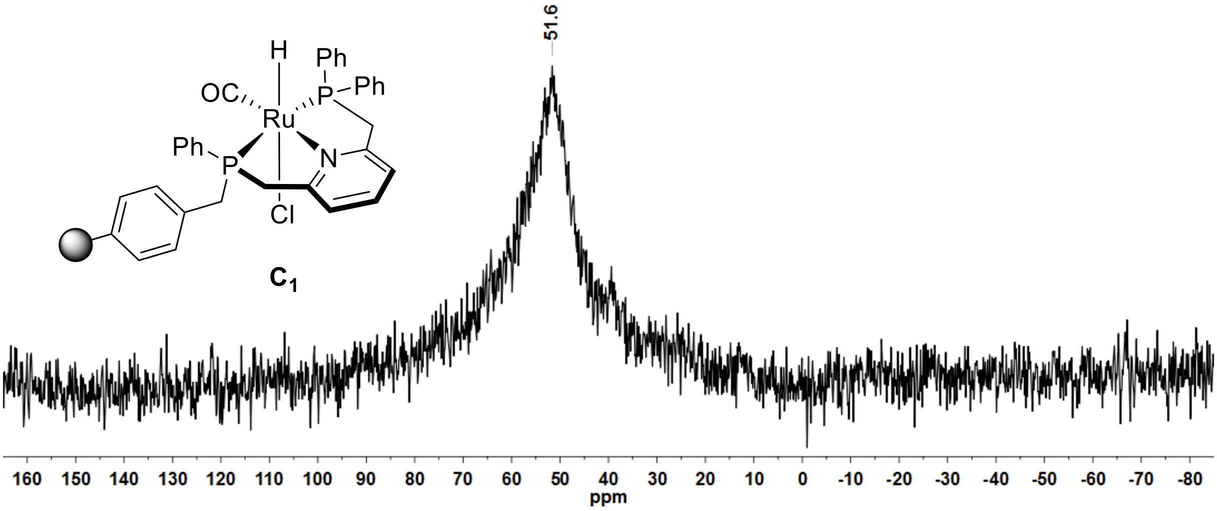

Figure SI 52 Gel-phase ^{31}P NMR of $\mathbf{C_1}$ (162 MHz, THF).

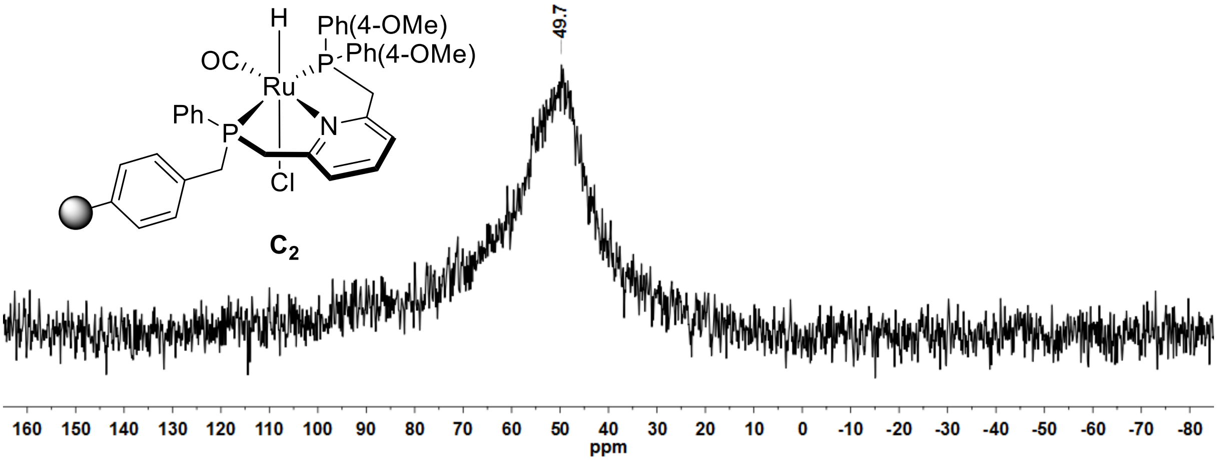

Figure SI 53 Gel-phase ^{31}P NMR of $\mathbf{C_2}$ (162 MHz, THF).

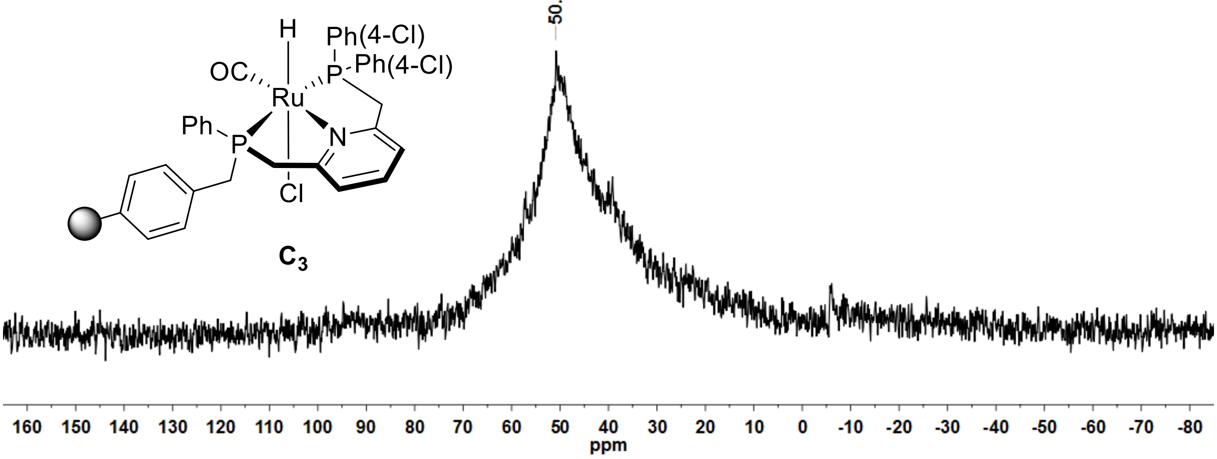

Figure SI 54 Gel-phase ^{31}P NMR of $\mathbf{C_3}$ (121 MHz, THF:C_6D_6 6:1).

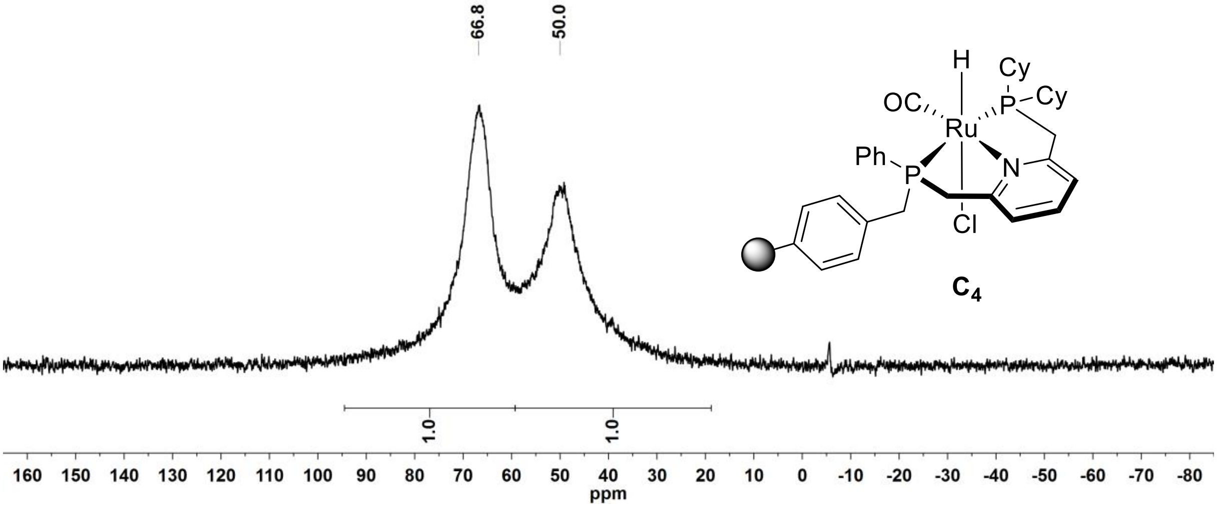

Figure SI 55 Gel-phase ^{31}P NMR of **C_4** (121 MHz, THF:C_6D_6 6:1).

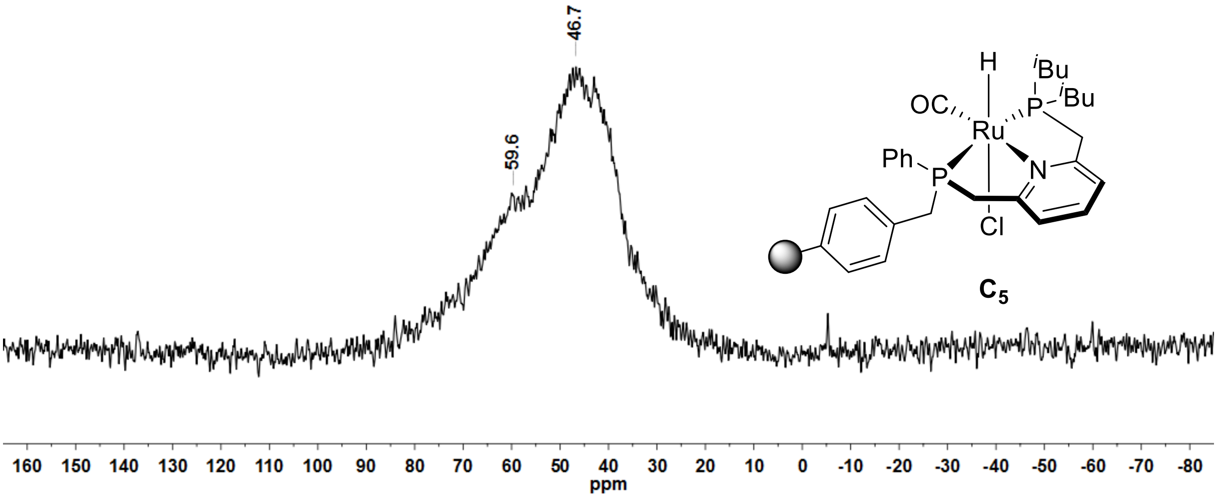

Figure SI 56 Gel-phase ^{31}P NMR of **C_5** (162 MHz, THF).

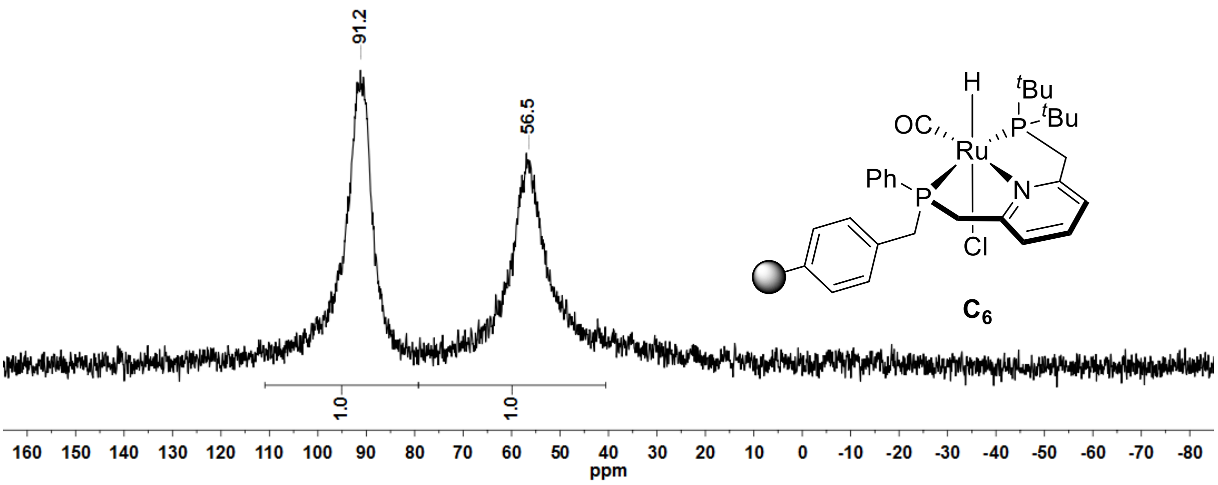

Figure SI 57 Gel-phase ^{31}P NMR of **C_6** (162 MHz, THF).

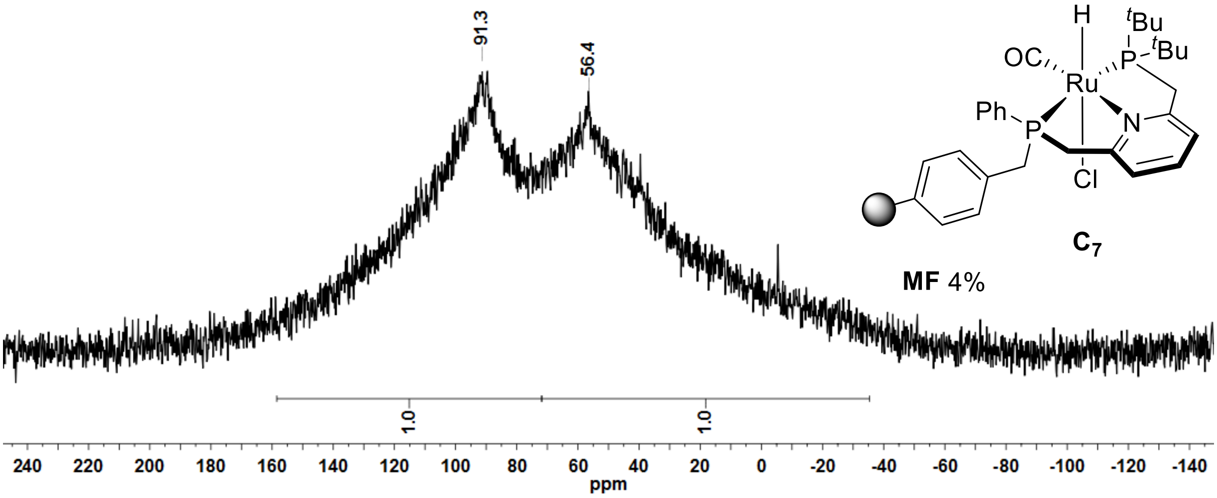

Figure SI 58 Gel-phase ^{31}P NMR of $\mathbf{C_7}$ (162 MHz, THF).

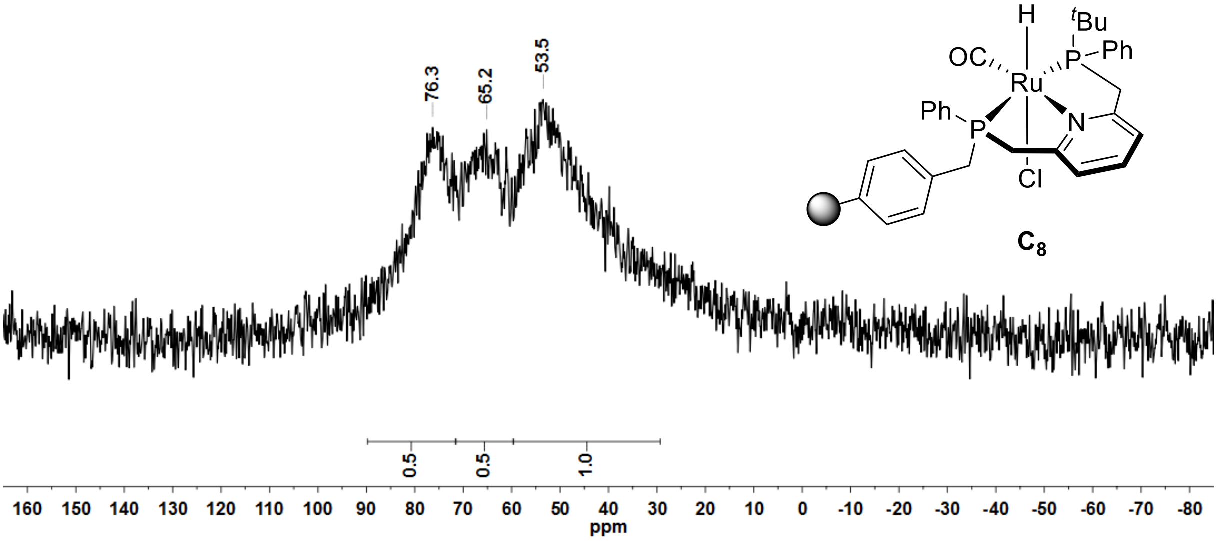

Figure SI 59 Gel-phase ^{31}P NMR of $\mathbf{C_8}$ (162 MHz, THF).

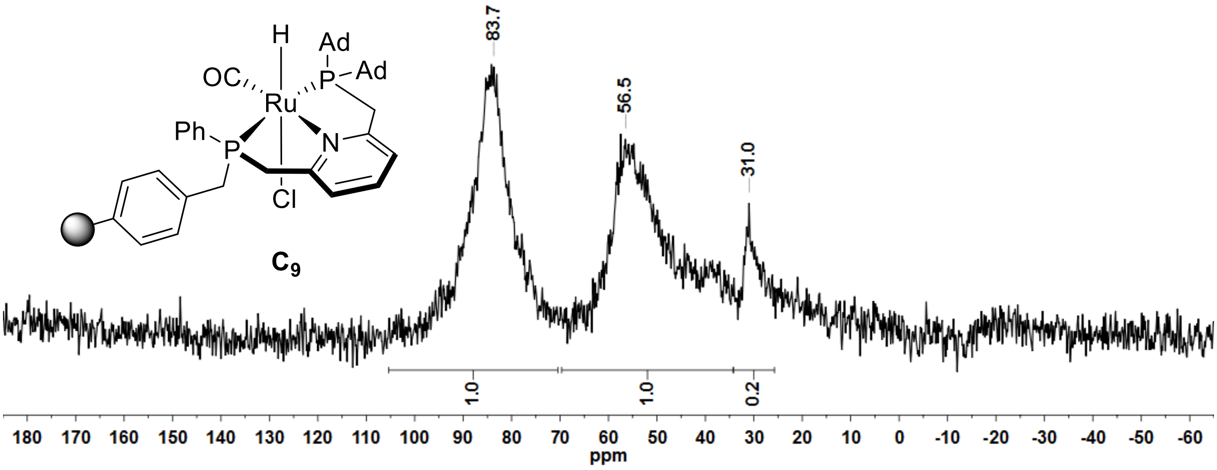

Figure SI 60 Gel-phase ^{31}P NMR of $\mathbf{C_9}$ and free **-***P*Ad$_2$ (162 MHz, THF).

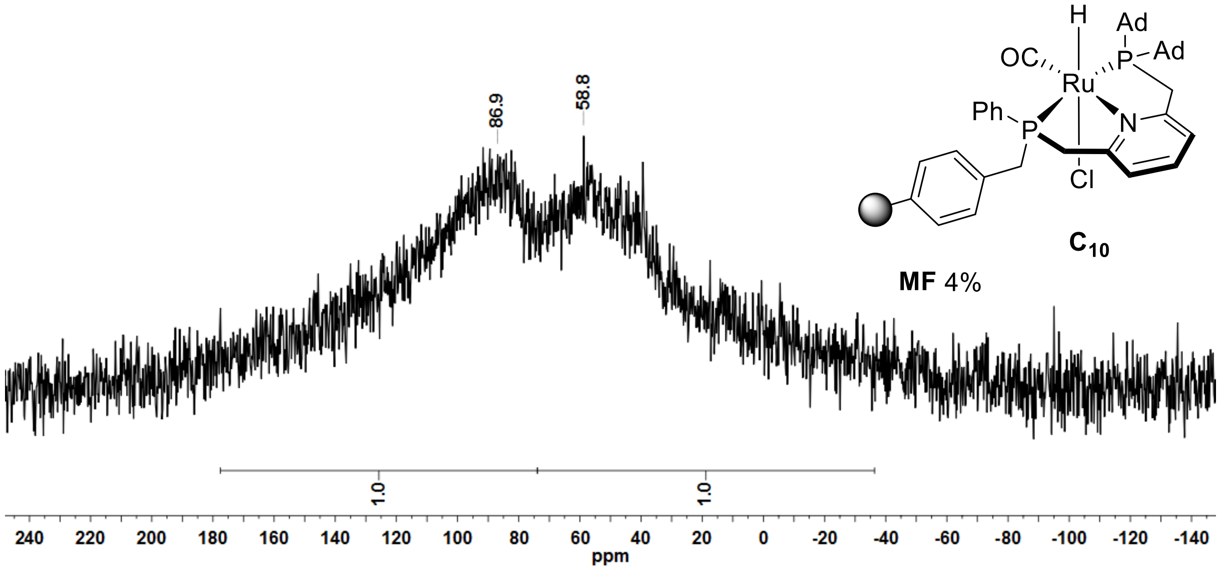

Figure SI 61 Gel-phase ^{31}P NMR of $\mathbf{C_{10}}$ (162 MHz, THF).

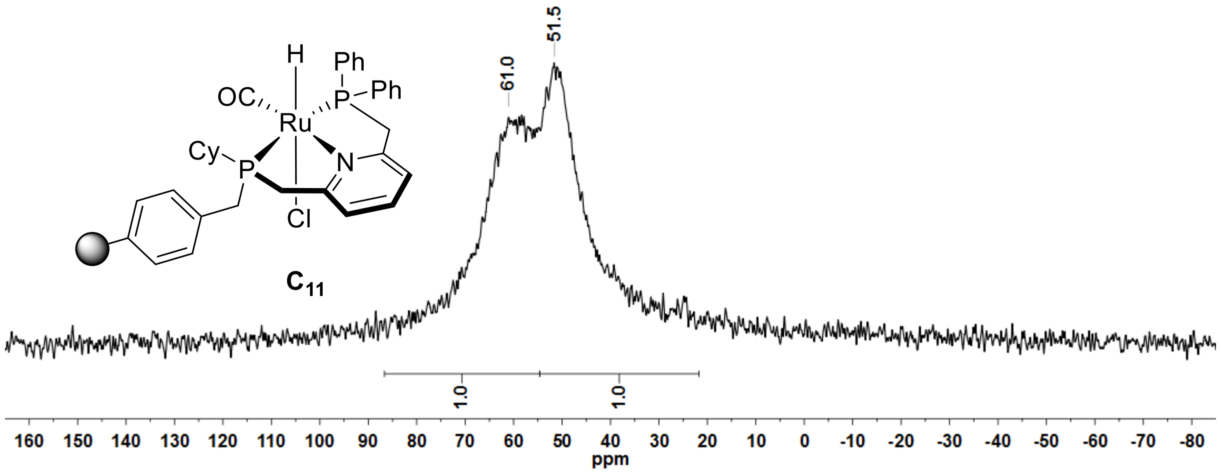

Figure SI 62 Gel-phase ^{31}P NMR of $\mathbf{C_{11}}$ (162 MHz, THF).

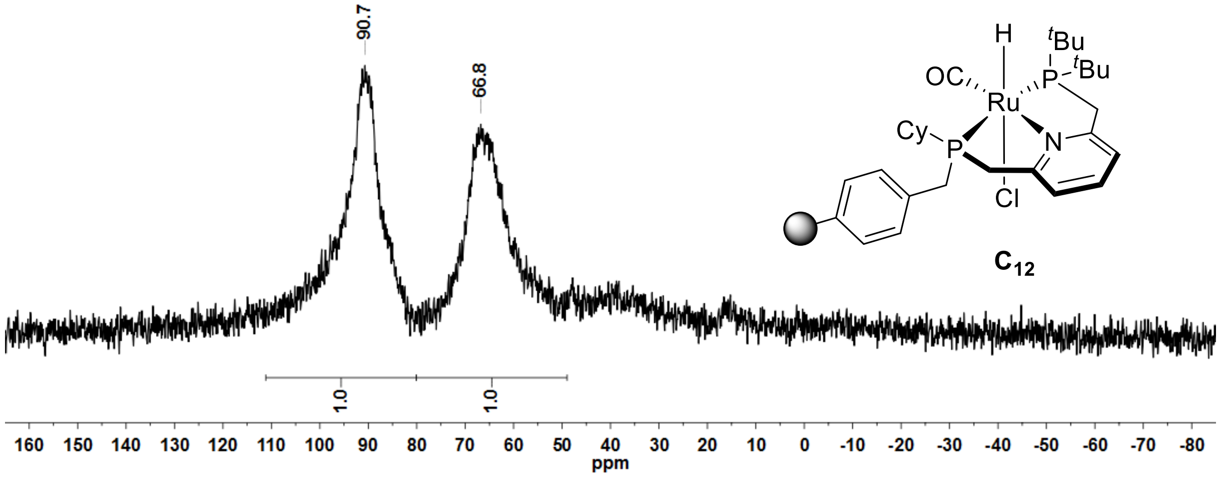

Figure SI 63 Gel-phase ^{31}P NMR of $\mathbf{C_{12}}$ (162 MHz, THF).

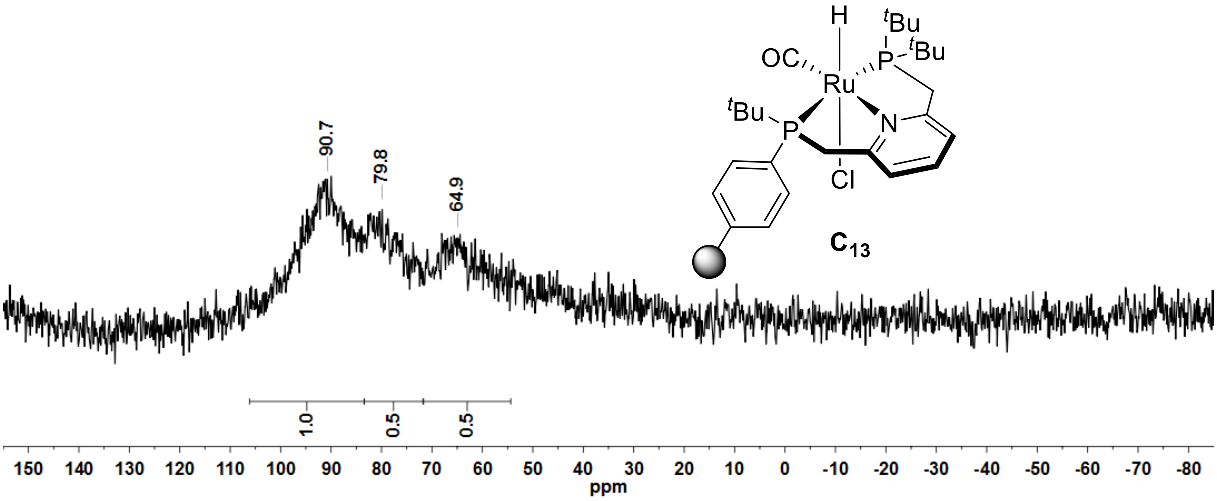

Figure SI 64 Gel-phase ^{31}P NMR of $\mathbf{C_{13}}$ (162 MHz, THF).

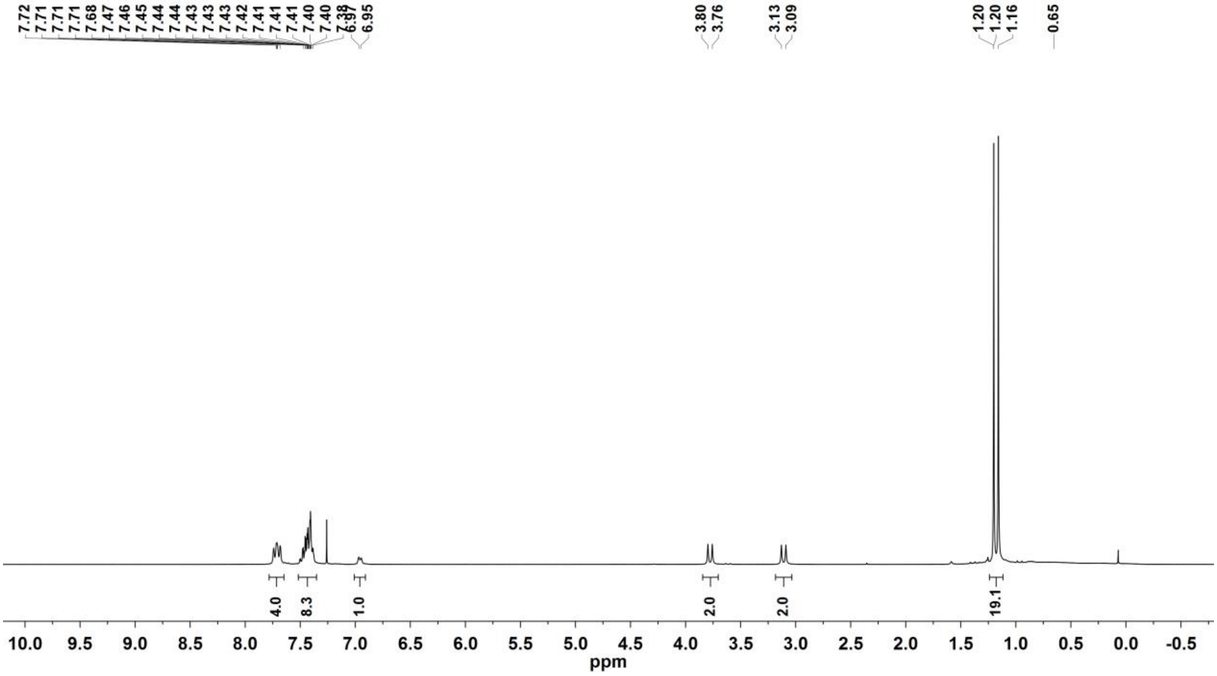

Figure SI 65 ^{1}H NMR spectrum of **4** (300 MHz, $CDCl_3$).

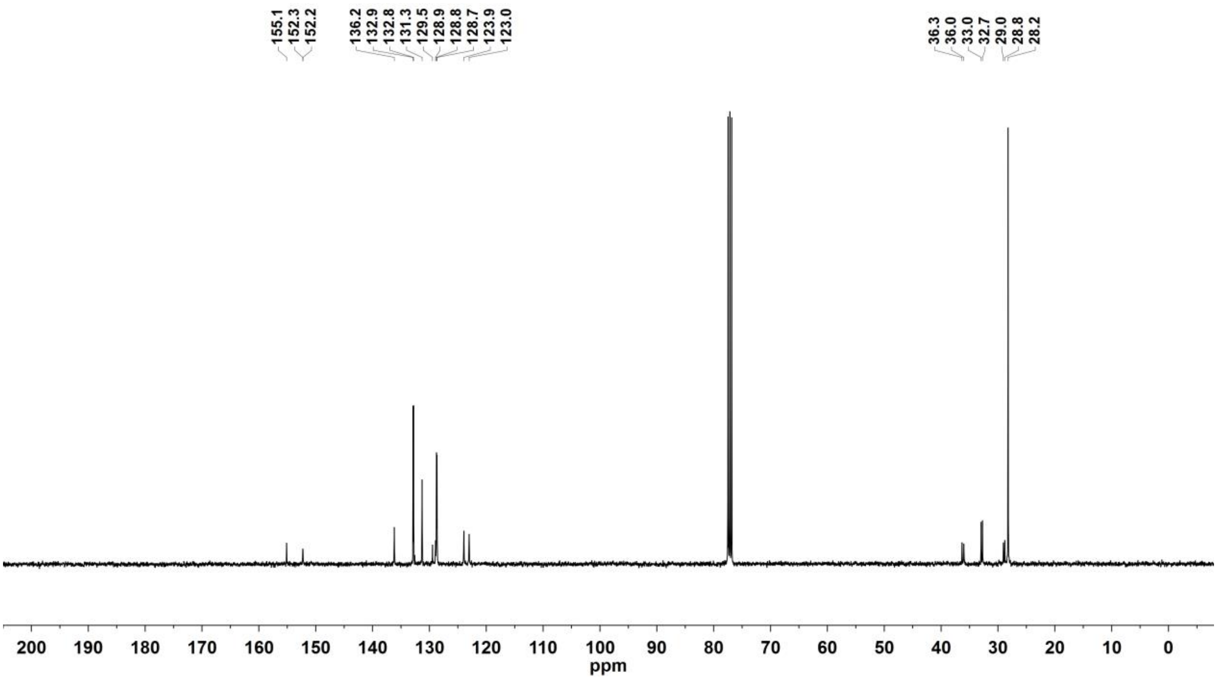

Figure SI 66 ^{13}C NMR of **4** (101 MHz, $CDCl_3$).

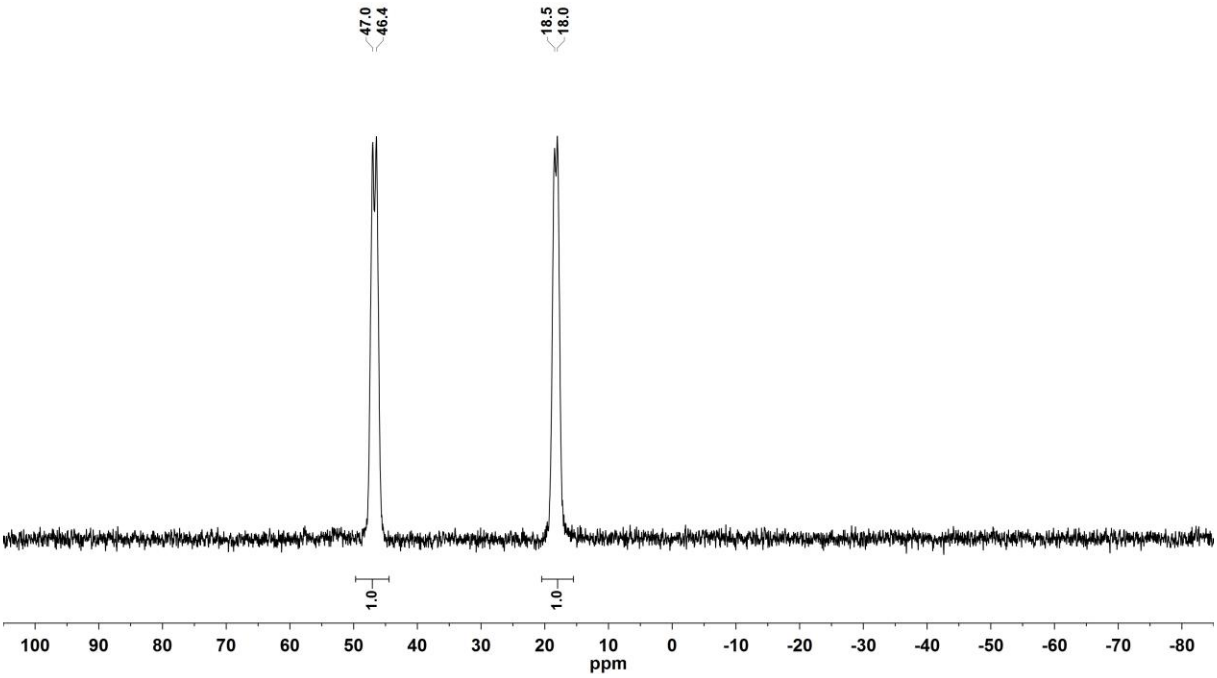

Figure SI 67 ^{31}P NMR of **4** (121 MHz, $CDCl_3$).

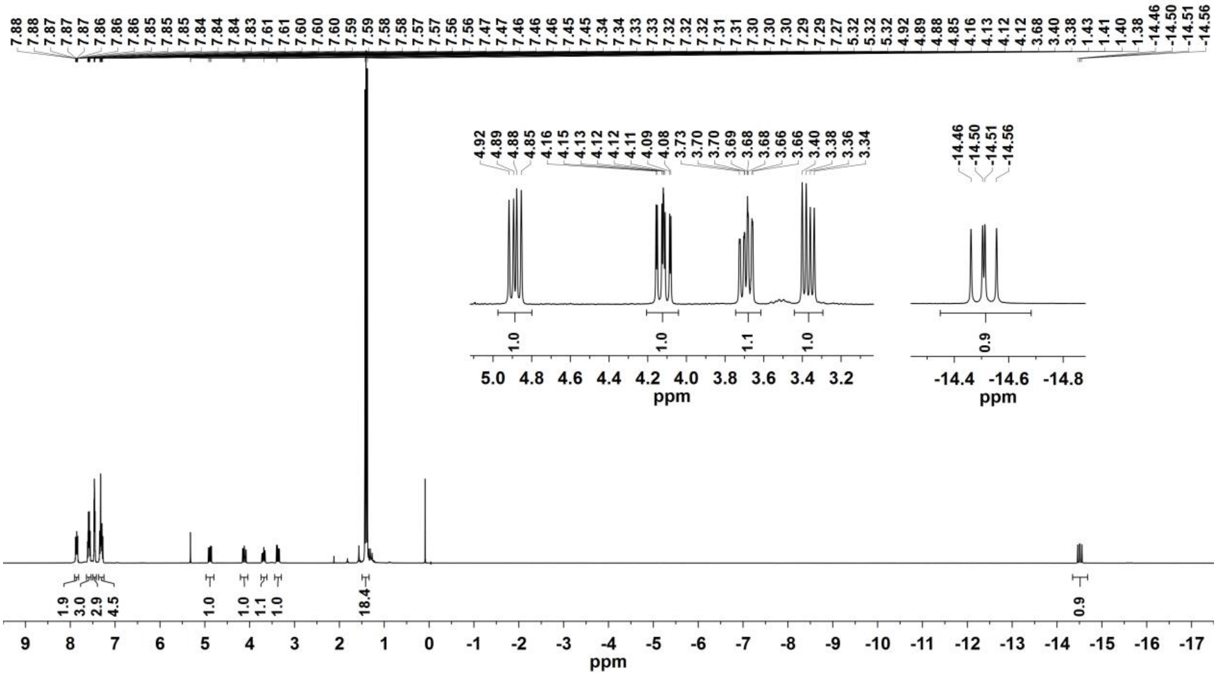

Figure SI 68 ^{1}H NMR of **5** (400 MHz, CD_2Cl_2).

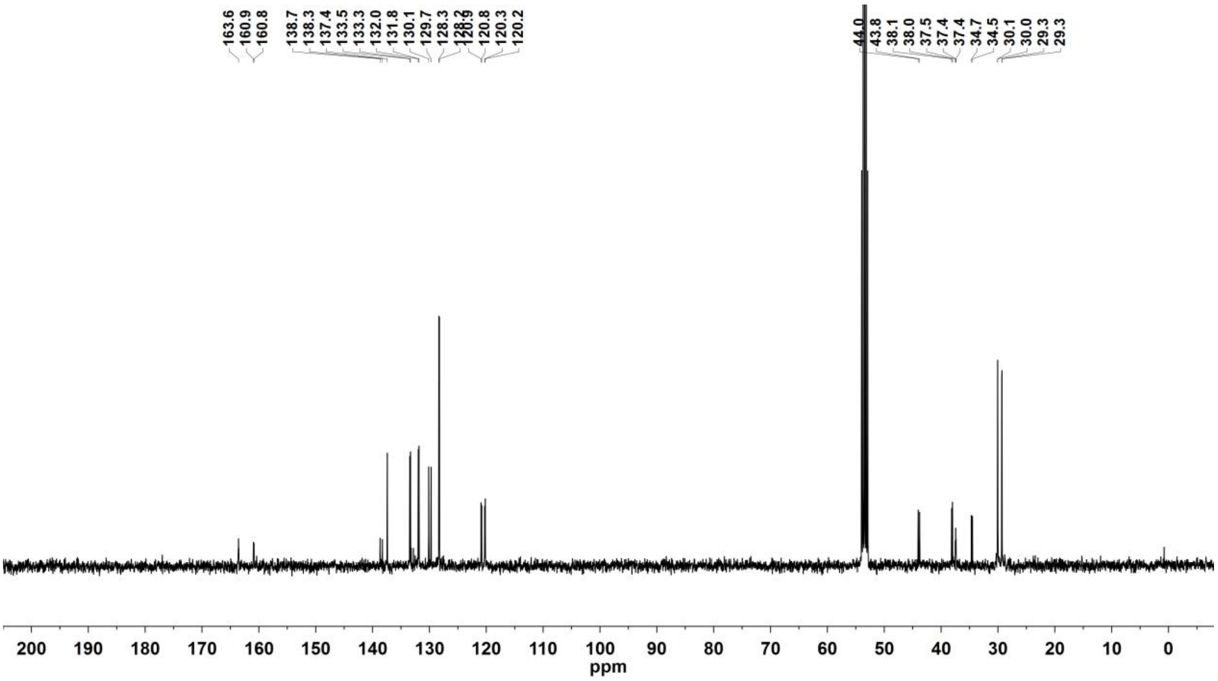

Figure SI 69 ^{13}C NMR of **5** (101 MHz, CD_2Cl_2).

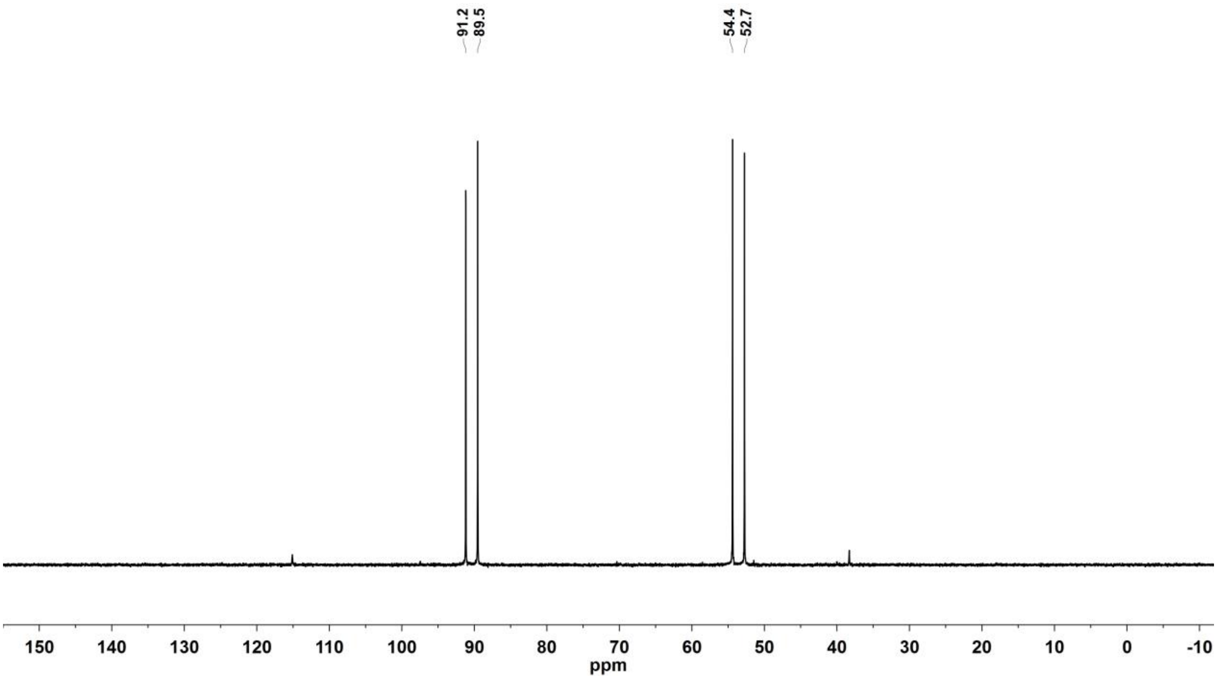

Figure SI 70 ^{31}P NMR of **5** (162 MHz, CD_2Cl_2).

IR Spectra

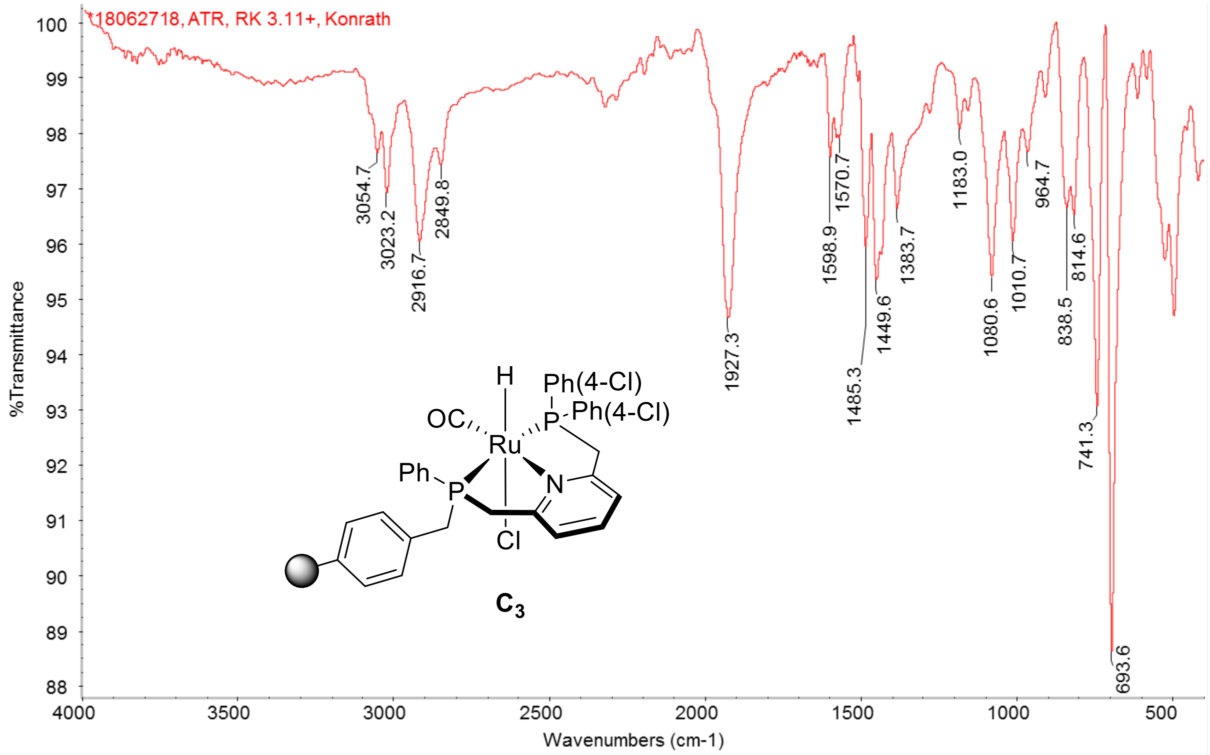

Figure SI 71 Representative IR spectrum of **C_3**.

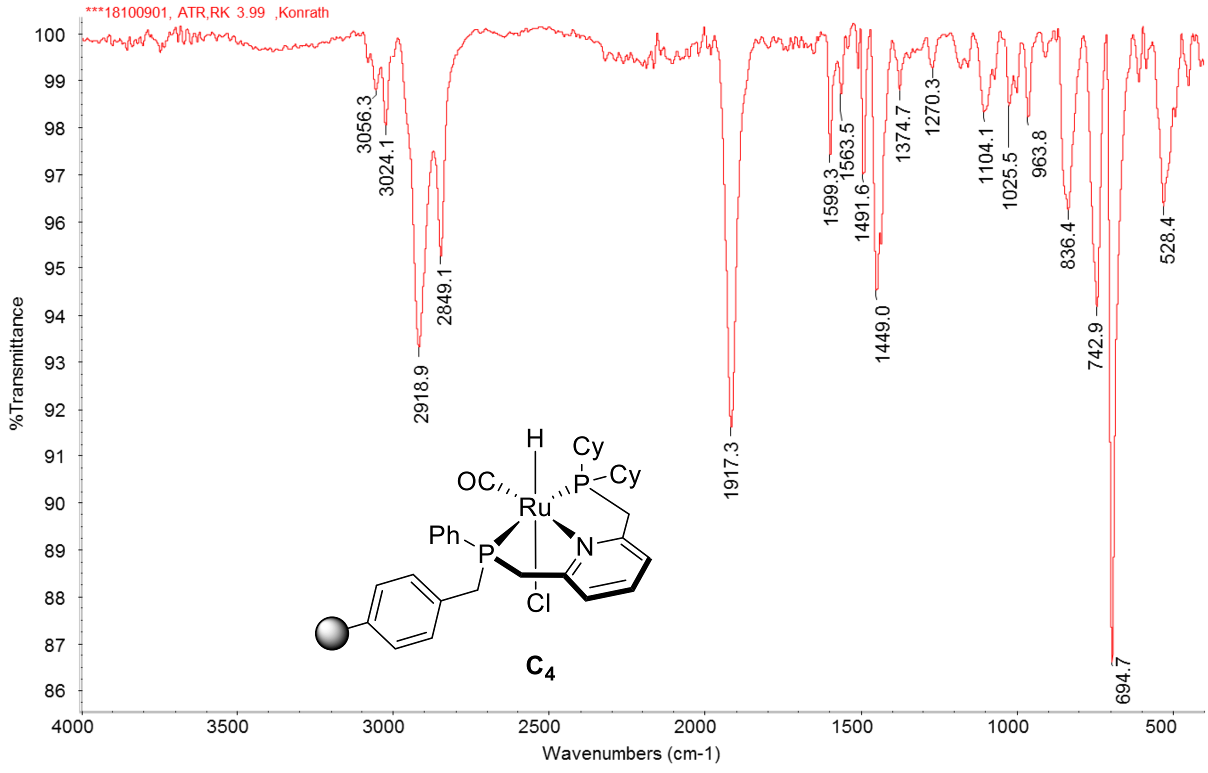

Figure SI 72 Representative IR spectrum of $\mathbf{C_4}$.

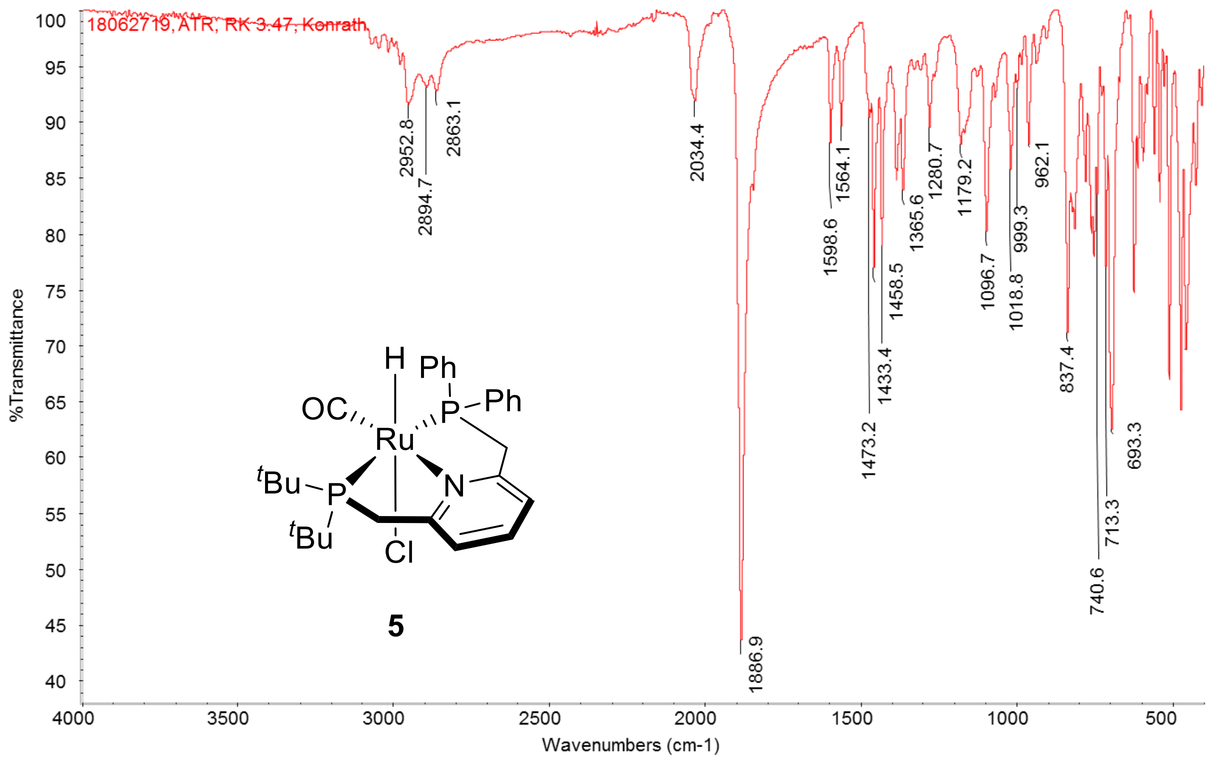

Figure SI 73 IR spectrum of **5**.

Representative GC-Traces of Ester Hydrogenation Experiments

Hydrogenation of Substrate $\mathbf{S_1}$

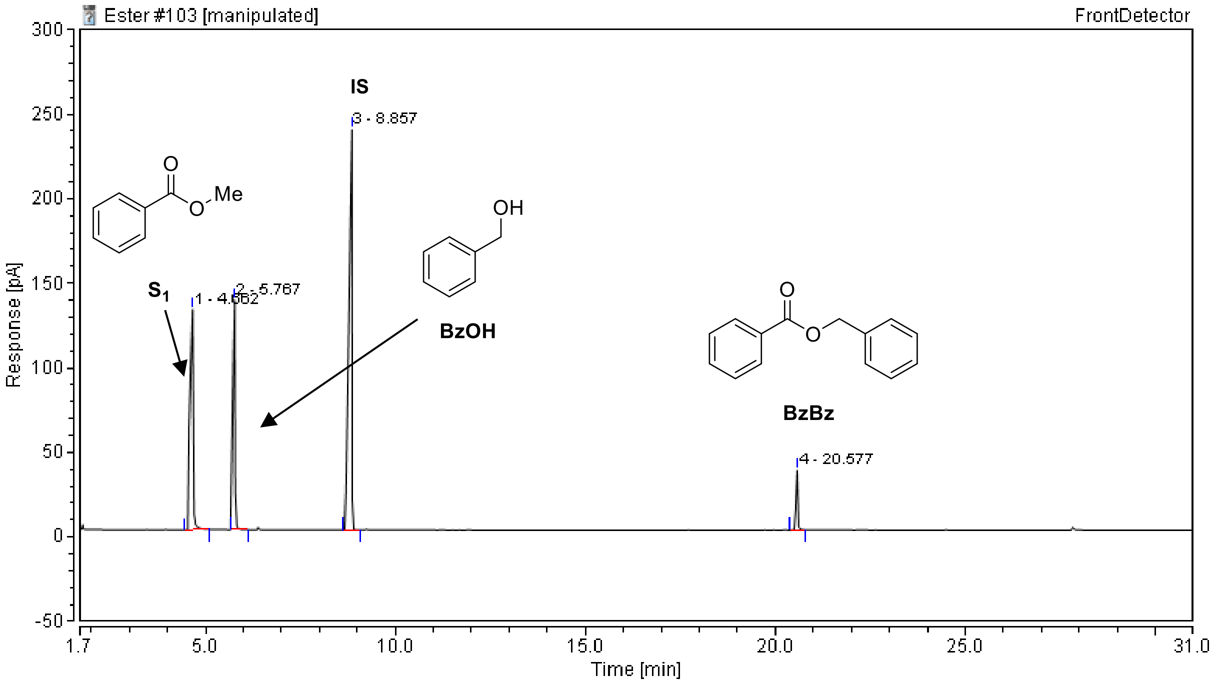

No.	Retention Time [min]	Area [pA·min]	Height [pA]	Relative Area [%]
1	4.662	11.736	130.266	26.33
2	5.767	8.277	135.874	18.57
3	8.857	22.712	236.764	50.96
4	20.577	1.840	35.306	4.13
Total:		44.564	538.210	100

Hydrogenation of Substrate **S**$_3$

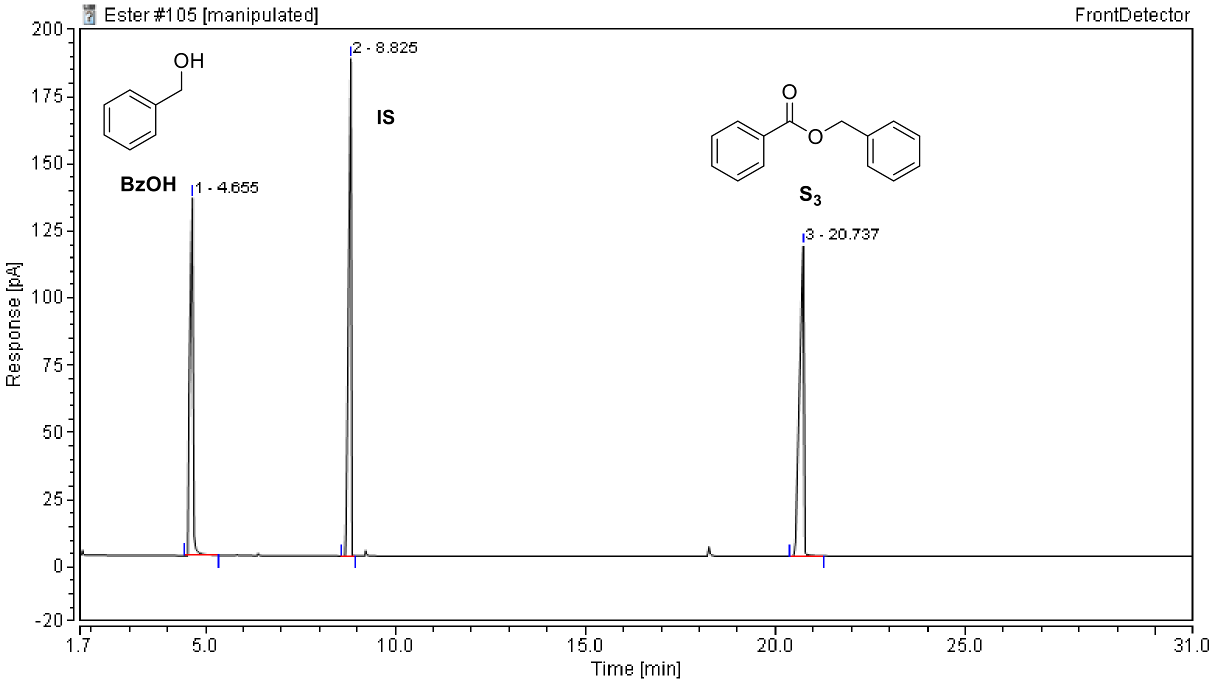

No.	Retention Time [min]	Area [pA·min]	Height [pA]	Relative Area [%]
1	4.655	11.039	133.010	28.21
2	8.825	14.066	185.011	35.95
3	20.737	14.023	115.352	35.84
Total:		39.128	433.373	100

Hydrogenation of Substrate **S_4**

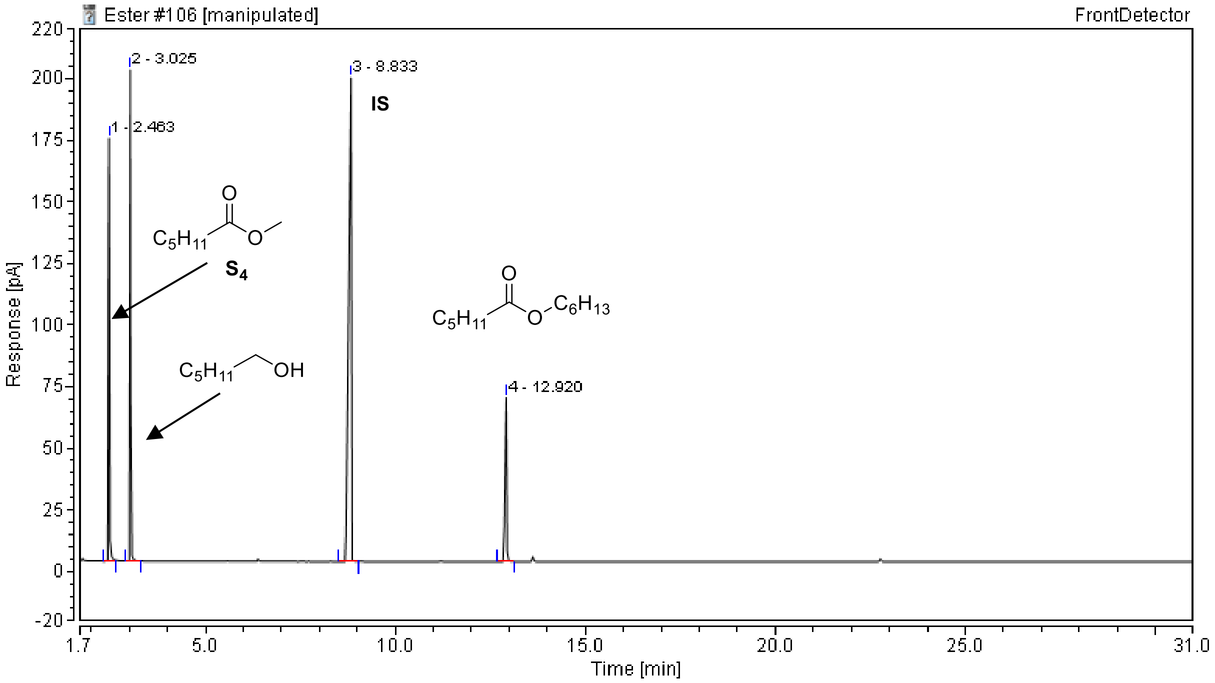

No.	Retention Time [min]	Area [pA·min]	Height [pA]	Relative Area [%]
1	2.463	4.256	171.483	15.13
2	3.025	4.764	199.279	16.93
3	8.833	15.755	196.251	56.01
4	12.920	3.354	66.660	11.92
Total:		28.129	633.674	100

Hydrogenation of Substrate **S_6**

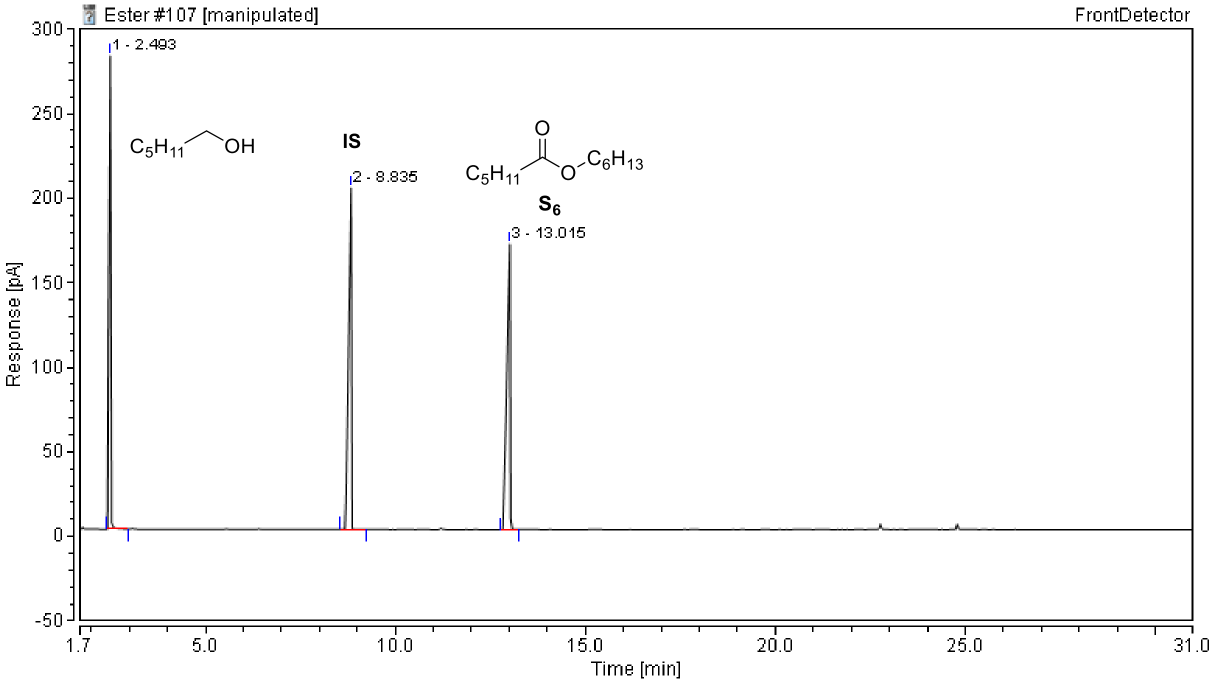

No.	Retention Time [min]	Area [pA·min]	Height [pA]	Relative Area [%]
1	2.493	11.685	280.130	25.90
2	8.835	16.813	201.990	37.27
3	13.015	16.616	168.896	36.83
Total:		45.114	651.016	100

Hydrogenation of Substrate **S$_9$**

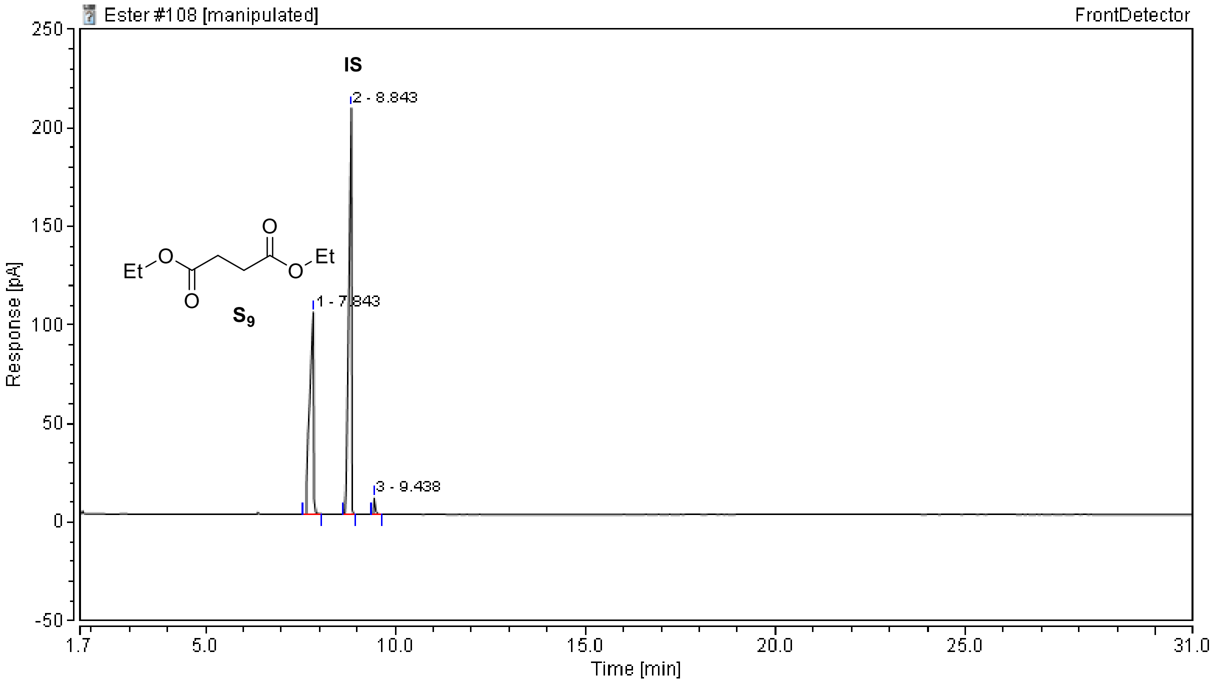

No.	Retention Time [min]	Area [pA·min]	Height [pA]	Relative Area [%]
1	7.843	12.477	102.353	100
Total:		12.477	316.733	100

X-Ray Crystallographic Data of 5

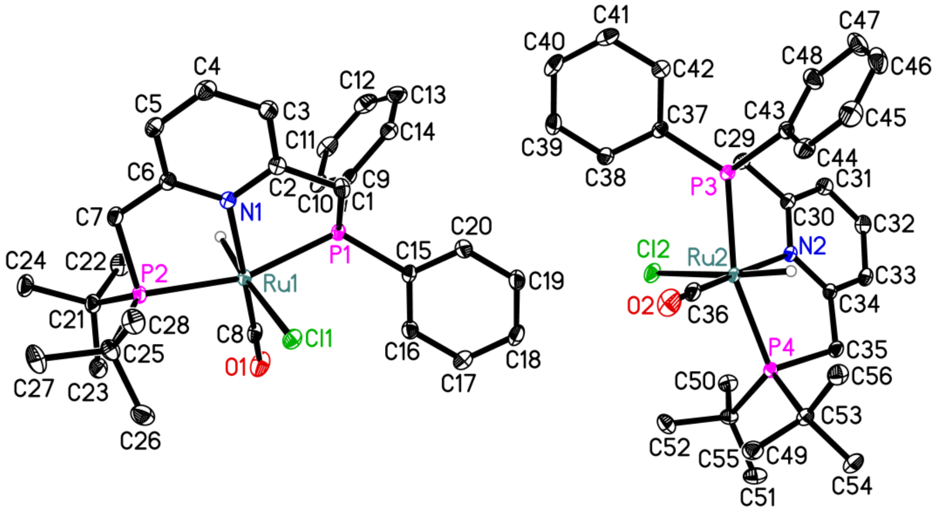

Figure SI 74 Molecular structure of **5** in the crystal. Displacement ellipsoids correspond to 30% probability. C-bound hydrogen atoms are omitted for clarity.

Data were collected on a Bruker Kappa APEX II Duo diffractometer. The structure was solved by direct methods (SHELXS-97)[5] and refined by full-matrix least-squares procedures on F^2 (SHELXL-2014).[6] XP (Bruker AXS) was used for graphical representations.

CCDC 1939716 contains the supplementary crystallographic data for this paper. These data are provided free of charge by The Cambridge Crystallographic Data Centre.

Empirical formula:	$C_{28}H_{36}ClNOP_2Ru$
Formula Weight:	601.04 g·mol^{-1}
Temperature:	150(2) K
Wavelength:	0.71073 Å
Crystal System:	monoclinic
Space group:	$P2_1/n$
Unit cell dimensions:	a = 16.732(3) Å, b = 11.269(2) Å, c = 29.356(5) Å
	α = 90 °, β = 100.462(3) °, γ = 90 °
Volume	5443.2(17) Å^3
Z	8
Density (calculated)	1.467 g/cm^3
Absorption coefficient	0.813 mm^{-1}
F(000)	2480
Crystal size	0.378 x 0.145 x 0.098 mm
Index ranges	-21<=h<=21, -14<=k<=14, -37<=l<=34
Reflexes collected	100155
Independent reflections	11889
Data/restraints/parameters	11889/2/633
Goodness-of-fit on F^2	1.046
Final R indices [I>2σ(I)]	R_1 = 0.0266, wR_2 = 0.0660
R indices (all data)	R_1 = 0.0340, wR_2 = 0.0707
Largest diff. peak and hole	1.059 and -0.312 e/Å^3

References

[1] a) F. J. L. Heutz, M. C. Samuels, P. C. J. Kamer, *Catal. Sci. Technol.* **2015**, *5*, 3296-3301; b) M. C. Samuels, F. J. L. Heutz, A. Grabulosa, P. C. J. Kamer, *Top. Catal.* **2016**, *59*, 1793-1799.

[2] P. E. Goudriaan, X. B. Jang, M. Kuil, R. Lemmens, P. W. N. M. v. Leeuwen, J. N. H. Reek, *Eur. J. Org. Chem.* **2008**, *2008*, 6079-6092.

[3] A. Panossian, H. Fernández-Pérez, D. Popa, A. Vidal-Ferran, *Tetrahedron: Asymmetry* **2010**, *21*, 2281-2288.

[4] M. Gargir, Y. Ben-David, G. Leitus, Y. Diskin-Posner, L. J. W. Shimon, D. Milstein, *Organometallics* **2012**, *31*, 6207-6214.

[5] G. M. Sheldrick, *Acta Cryst.* **2008**, *A64*, 112-122.

[6] G. M. Sheldrick, *Acta Cryst.* **2015**, *C71*, 3-8.
